# Integrating UPLC-Q-TOF-MS and Network Pharmacology to Explore the Potential Mechanisms of *Paeonia lactiflora* Pall. in the Treatment of Blood Stasis Syndrome

**DOI:** 10.3390/molecules29133019

**Published:** 2024-06-26

**Authors:** Mengzhen Ma, Qianqian Du, Suying Shi, Jiahui Lv, Wei Zhang, Dezhu Ge, Lihua Xing, Nianjun Yu

**Affiliations:** 1 School of Pharmacy, Anhui University of Chinese Medicine, Hefei 230012, China; mmz13733081879@163.com (M.M.); eryemore@163.com (Q.D.); ljhbir0716@sina.com (S.S.); ljh@stu.ahtcm.edu.cn (J.L.); aa242stus@sina.com (W.Z.); 2 MOE-Anhui Joint Collaborative Innovation Center for Quality Improvement of Anhui Genuine Chinese Medicinal Materials, Hefei 230012, China; 3 Anhui Province Key Laboratory of Research & Development of Chinese Medicine, Hefei 230012, China; 4 Anhui Jiren Pharmaceutical Co., Ltd., Bozhou 236800, China; dgh199704@163.com

**Keywords:** *Paeonia lactiflora* Pall, blood stasis syndrome, UPLC-Q-TOF-MS, network pharmacology, molecular docking

## Abstract

*Paeonia lactiflora* Pall. (PLP) is thought to promote blood circulation and remove blood stasis. This study used blood component analysis, network pharmacology, and molecular docking to predict the mechanism of PLP in the treatment of blood stasis syndrome (BSS). PLP was processed into *Paeoniae Radix* Alba (PRA) and *Paeoniae Radix* Rubra (PRR). PRA and PRR could significantly reduce whole blood viscosity (WBV) at 1/s shear rates and could increase the erythrocyte aggregation index (EAI), plasma viscosity (PV), and erythrocyte sedimentation rate (ESR) of rats with acute blood stasis. They prolonged the prothrombin time (PT), and PRR prolonged the activated partial thromboplastin time (APTT). PRA and PRR increased the thrombin time (TT) and decreased the fibrinogen (FBG) content. All the results were significant (*p* < 0.05). Ten components of Paeoniflorin, Albiflorin, Paeonin C, and others were identified in the plasma of rats using ultra-high-performance liquid chromatography–quadrupole time-of-flight mass spectrometry (UPLC-Q-TOF-MS). A protein–protein interaction network (PPI) analysis showed that AKT1, EGFR, SRC, MAPK14, NOS3, and KDR were key targets of PLP in the treatment of BSS, and the molecular docking results further verified this. This study indicated that PLP improves BSS in multiple ways and that the potential pharmacological mechanisms may be related to angiogenesis, vasoconstriction and relaxation, coagulation, and the migration and proliferation of vascular cells.

## 1. Introduction

*Paeonia lactiflora* Pall. (PLP) is a perennial herb of the genus Paeonia in the family Paeoniaceae. PLP has a long history of medicinal use that can be traced back to the prescriptions for Fifty-two Ailments in the Spring and Autumn period. Shen Nong’s Classic of Medicinal Herbs, the first pharmaceutical monograph in China, recorded that PLP has the functions of relieving pain and diuresis, promoting blood circulation, and removing blood stasis [1,2]. Over the cultural development of traditional Chinese medicine (TCM), the people’s understanding of many Chinese herbal medicines and the use of *Paeoniae Radix* Alba (PRA) and *Paeoniae Radix* Rubra (PRR) have undergone a dynamic process. PLP was not separated initially and gradually evolved into the processed products PRA and PRR based on the experiences of doctors across the ages. According to the 2020 edition of the Chinese Pharmacopoeia, PRA can be obtained by boiling PLP in water at 100 °C and removing the outer skin, then boiling and drying. PRR can be obtained by washing PLP and drying directly. PLP is the original plant of PRA and PRR [3]. Modern studies have shown that both PRA and PRR could promote blood circulation and remove blood stasis [4,5].

Blood stasis syndrome (BSS), which is called “Xueyu Zheng” in China, is an important pathological condition in TCM [6]. Modern medical research has shown that the pathological changes in BSS are related to abnormal hemorheology, including changes in blood viscosity, hematocrit (HCT), the erythrocyte sedimentation rate (ESR), the erythrocyte aggregation index (EAI), and the blood coagulation function [7]. Furthermore, it has been reported that BSS is closely related to thrombosis, inflammation, edema, and the immune response, which are caused by abnormal blood circulation and the viscous states of systemic or local tissues and organs. The continuous progression of BSS can induce a variety of diseases, including hypertension, coronary heart disease, cerebral infarction, inflammatory infection, tumors, and cancer [8]. Therefore, it is of great significance to explore the therapeutic effect and mechanism of PLP in BSS.

Network pharmacology can be applied to define diseases and the mechanisms of action of drugs from the standpoint of a biological network. It emphasizes analysis of the law of molecular correlations between drugs and therapeutic targets at the system level and within a biological network. Its research concept is consistent with the holistic theory of TCM and has been used widely in the discovery of drugs and active compounds of TCM, the interpretation of their overall mechanisms of action, and the analysis of the law of drug combination and prescription compatibility. It provides new ideas for the research of complex systems of TCM and provides new scientific and technological support for the rational clinical use of drugs and the research and development of new drugs [9,10,11]. Molecular docking uses computer technology to simulate the binding between ligands (proteins, DNA/RNA, and small molecules) and biological receptor protein macromolecules and then predicts their binding modes and affinities according to calculations of physical and chemical parameters. Its essence is the process of mutual recognition between two or more molecules, which involves spatial matching and energy matching between the molecules [12]. This combination can systematically predict the possible mechanism of PLP in the treatment of BSS.

In this study, an acute blood stasis model was established to verify the effects of PRA and PRR on BSS. Taking the components entering the blood as the research object, network pharmacology and molecular docking technology were used to construct drug and disease target networks, and the potential mechanism of action and key targets of PLP were predicted. From the perspective of UPLC-Q-TOF-MS determination of blood components, combined with network pharmacology and a molecular docking prediction mechanism, this paper provides a theoretical basis for further study of PLP in the treatment of BSS.

## 2. Results and Discussion

### 2.1. Evaluation of Model

**Figure 1:**
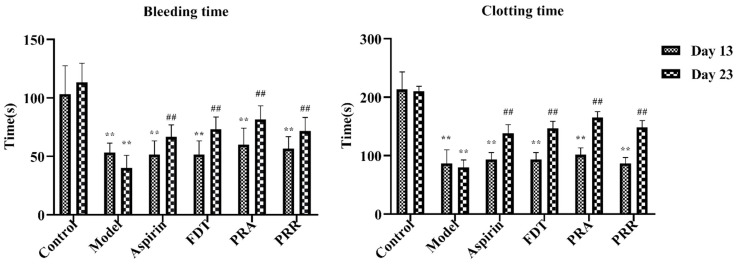
Bleeding and clotting times on day 13 and day 23. Fufang Danshen tablets (FDT); *Paeoniae Radix* Alba (PRA); *Paeoniae Radix* Rubra (PRR) (compared to the control group, ** *p* < 0.01; compared to the model group, ## *p* < 0.01).

To evaluate the success of the blood stasis model and the effectiveness of administration in each group, blood samples were taken from the tail veins of the rats in each group, and the bleeding and in vitro clotting times were measured. As shown in Figure 1, compared to the control group, the tail vein bleeding and in vitro clotting times of the rats in each group were significantly shortened, and there was a significant statistical difference on day 13 of the experiment, indicating that the blood stasis rat model was successfully replicated. In addition, as shown in Figure 1, compared to the model group, the bleeding and clotting times of the rats in each administration group increased significantly on day 23 of the experiment, indicating that all drugs were effective. Details of the raw data are shown in Table S1.

### 2.2. Hemorheology and Evaluation of Related Functional Parameters 

**Figure 2:**
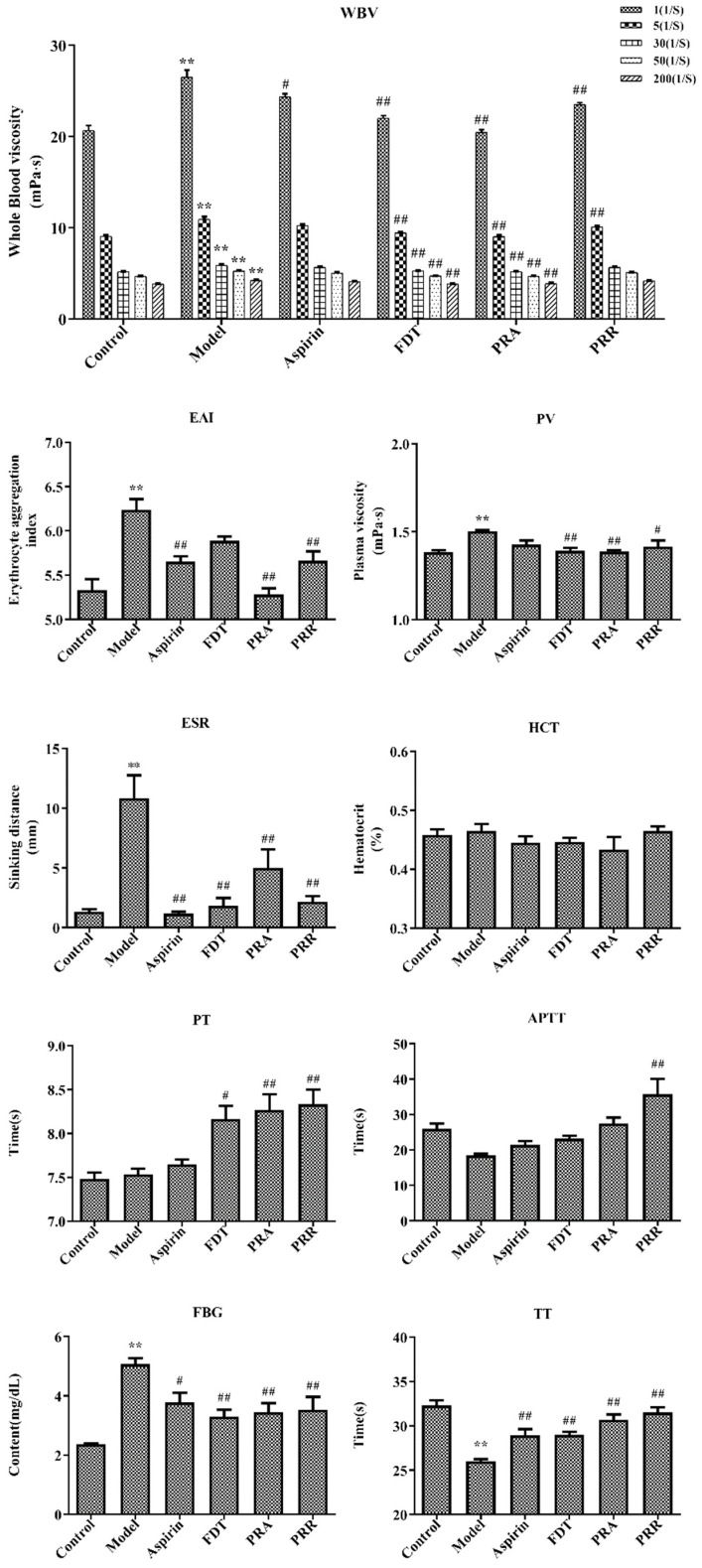
Hemorheology and related functional parameters. Whole blood viscosity (WBV); erythrocyte aggregation index (EAI); plasma viscosity (PV); erythrocyte sedimentation rate (ESR); hematocrit (HCT); prothrombin time (PT); activated partial thromboplastin time (APTT); fibrinogen (FBG); thrombin time (TT) (compared to the control group, ** *p* < 0.01; compared to the model group, # *p* < 0.05, ## *p* < 0.01).

Hemorheology and blood coagulation indexes are the main indexes for the diagnosis of BSS. Hemorheology reflects changes in blood fluidity, coagulation, and viscosity caused by changes in blood composition. With a decrease in blood viscosity and an improvement in blood fluidity, the coagulation time will be prolonged accordingly, which also reflects the activity of drugs that promote blood circulation and remove blood stasis [13]. In this study, a BSS model including viscosity, concentration, coagulation, and aggregation of hemorheology was replicated via subcutaneous injections of high-dose epinephrine hydrochloride and ice water stress in rats. The results of hemorheology indexes including whole blood viscosity (WBV), EAI, plasma viscosity (PV), ESR, and HCT and blood coagulation indexes including the prothrombin time (PT), the activated partial thromboplastin time (APTT), fibrinogen (FBG), and the thrombin time (TT) are shown in Figure 2. Details of the raw data are shown in Tables S2 and S3.

As shown in Figure 2, compared with the control group, the WBV, including at 1/s, 5/s, 30/s, 50/s, and 200/s shear rates, increased significantly (*p* < 0.01) in the model group. Compared to the model group, PRA could significantly reduce WBV at each shear rate (*p* < 0.01), and PRR could significantly reduce WBV at the 1/s and 5/s rates (*p* < 0.01). Compared to the control group, the EAI, PV, and ESR increased significantly in the model group, and the administration of PRA and PRR improved this upregulation significantly (*p* < 0.05). However, they had little effect on HCT, which indicated that the blood stasis model had little effect on HCT.

As shown in Figure 2, compared with the control group, there were no significant changes in the PT and APTT in the model group, indicating that the blood stasis model had little impact on the factors involved in the exogenous and endogenous pathways of coagulation. However, the PRA and PRR groups prolonged the PT significantly (*p* < 0.01), and PRR prolonged the APTT significantly (*p* < 0.01), indicating that PRA and PRR could prolong the coagulation time of the exogenous pathway, and PRR could prolong the coagulation time of the endogenous pathway. Also, PRA could prolong the APTT, though this effect was not significant (*p* > 0.05). Furthermore, compared to the control group, the TT in the model group decreased significantly, and the FBG content increased significantly (*p* < 0.01). Compared to the model group, PRA and PRR could increase the TT and decrease the FBG content significantly (*p* < 0.01). Prolonged TT is more common in the presence of decreased FBG levels or structural abnormalities.

The above results showed that both PRA and PRR could improve BSS.

### 2.3. UPLC-Q-TOF-MS Analysis

To accurately identify the different components of PRA and PRR, UPLC-Q-TOF-MS spectra were evaluated in positive and negative ion modes. The main peak compounds were identified using high-resolution mass spectrometry and the formula finder and master view functions of the Peak View2.2 mass spectrometry analysis software. Based on the retention time and mass spectrometric characteristics of the reference substance, as well as the information on MS primary mass spectrometry and MS/MS secondary mass spectrometry, the components were confirmed. In addition, according to reports from the relevant literature and the results of the in vitro component analysis, the components were further identified and confirmed by comparing their mass spectrometry information. 

**Figure 3:**
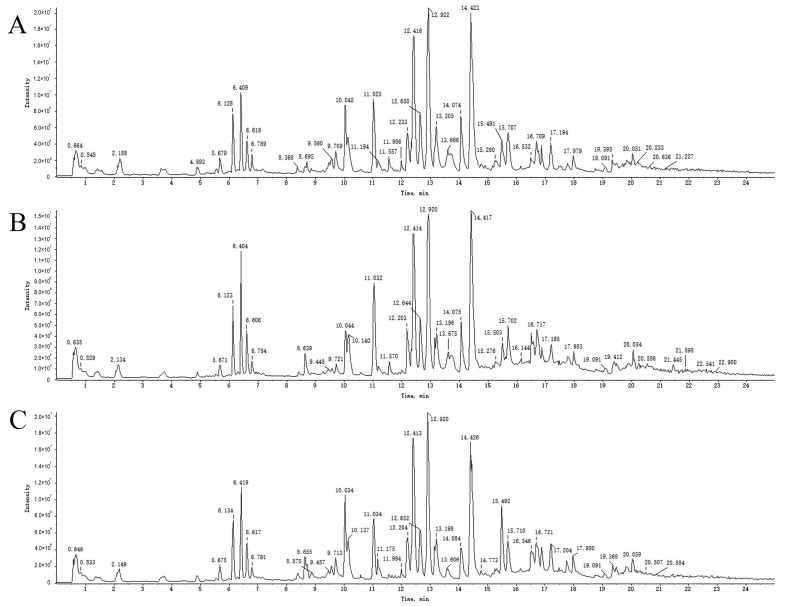
TICs of blank rat serum (**A**) and drug-containing PRA (**B**) and PRR (**C**) sera in positive ion mode.

**Figure 4:**
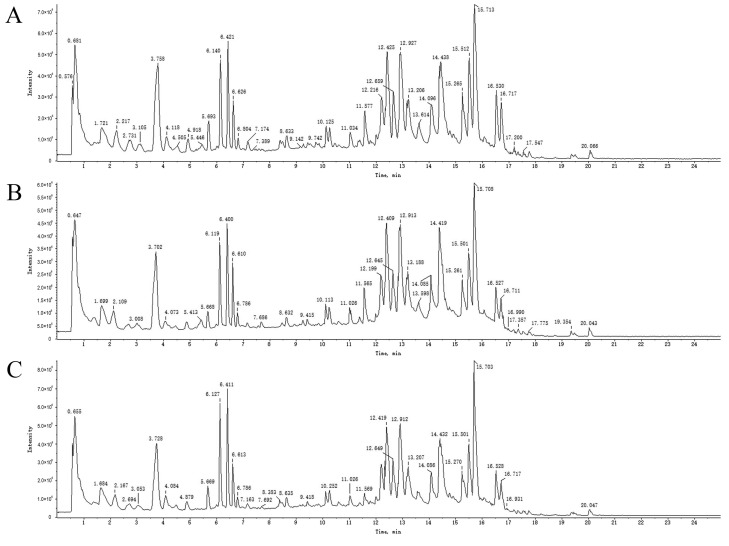
TICs of blank rat serum (**A**) and drug-containing PRA (**B**) and PRR (**C**) sera in negative ion mode.

An overall ion chromatogram in the positive ion mode is shown in Figure 3. In total, 55 compounds were identified. Moreover, based on the analysis of the data collected using the negative ion model, 39 compounds were identified. A total ion flow diagram is shown in Figure 4. The total ion chromatogram in the positive ion mode provided more compound information.

As shown in Table 1, 10 components of PRA and PRR were identified in the drug-containing serum. Guaiphenesin, albiflorin, paeonin C, paeonidaninol A, (+)-paeonilactone C, benzoic acid, and gallic acid were common components. Naringenin-7-*O*-glucoside and 5-hydroxyisovanillic acid were only identified in the PRA group, while paeoniflorin only existed in the PRR group. UPLC-Q-TOF-MS analysis revealed that the major constituents of PRA and PRR were monoterpene glycosides, polyphenols, and organic acids.

### 2.4. Analysis and Identification of Compounds in Drug-Containing Serum

**Figure 5:**
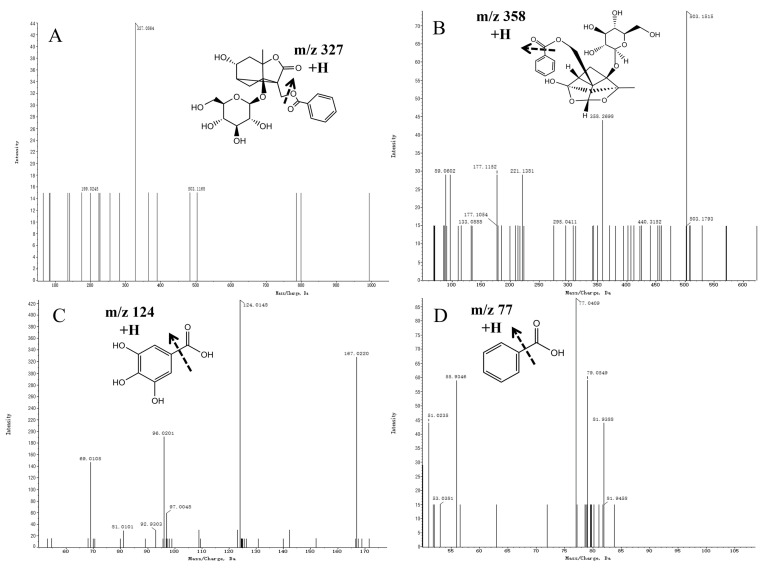
Mass spectra of precursor ions and product ions of Albiflorin (**A**), Paeoniflorin (**B**), Gallic acid (**C**), and Benzoic acid (**D**).

The mass spectrometric fragmentation patterns of paeoniflorin and its derivatives were similar. The basic skeleton fragment ions of pinane were characteristic fragment ions of paeoniflorin derivatives and produced fragment ions of different groups such as Glu and benzoic acid (HA). Compound **3** was inferred to be albiflorin based on ions generated with an *m/z* of 503.1510 [M + Na] + in the MS spectra and an *m/z* of 327.0864 [M + Na-CH_2_O-HA] + in the MS/MS spectra. Compound **10** was defined as paeoniflorin based on its ions with an m/z of 503.3051 [M + Na] + in the MS spectra and *m/z* values of 358 [M + Na-HA-Na] + and 221 [M + Na-HA-Glu] + in the MS/MS spectra. Referring to the standard cracking law and previous studies, compounds **3** and **10** were identified as albiflorin and paeoniflorin, respectively. Furthermore, the secondary mass spectrograms and main fragmentation rules of albiflorin and paeoniflorin are shown in Figure 5A, B.

Compound **8** was inferred to be gallic acid based on ions with an *m/z* of 169.0137 [M-H]- in the MS spectra and an *m/z* of 125.0303 [M-H-CHO_2_]- in the MS/MS spectra (Figure 5C). Compound **7** was inferred to be benzoic acid based on its ions with an *m/z* of 123.0436 [M + H] + in the MS spectra and an *m/z* of 77.0409 [M + H-CHO_2_] + in the MS/MS spectra (Figure 5D).

Due to a lack of reference standards, compounds **1**, **2**, **4**, **5**, **6**, and **9** were temporarily assigned before being subjected to accurate evaluations of their qualities and characteristics to diagnose fragment ions and identify them with references. The identification results and references of the compounds are listed in Table 1.

### 2.5. Target Screening of Components Entering the Blood

**Figure 6:**
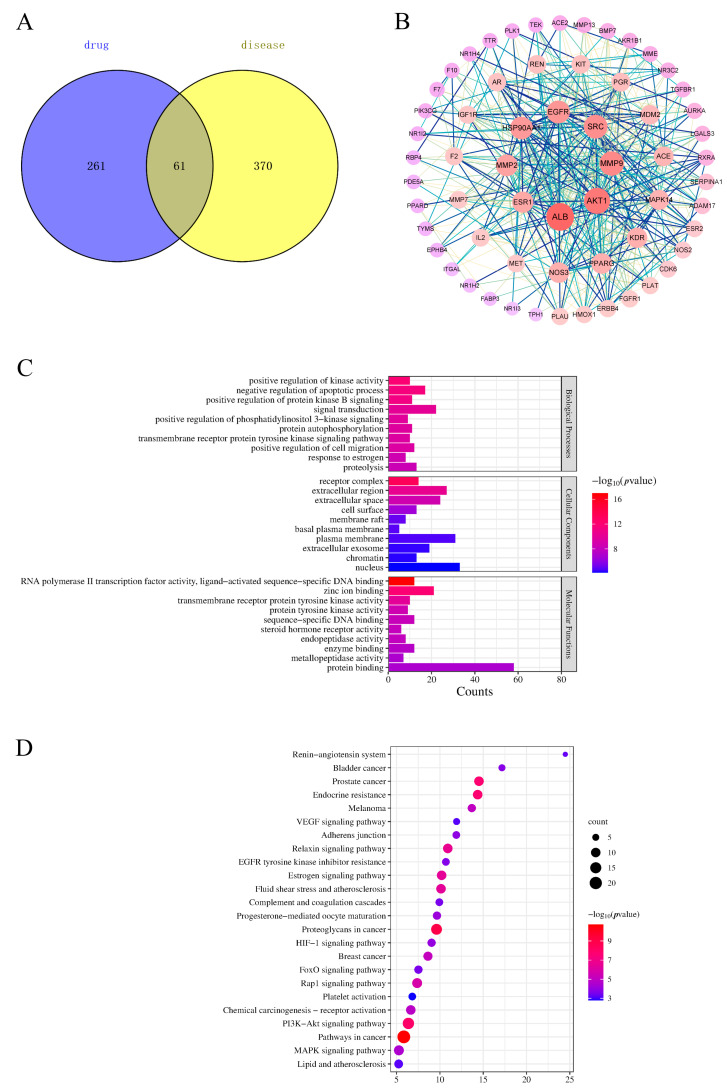
Venn diagram (**A**), protein–protein interaction (PPI) network (**B**), Gene Ontology (GO) (**C**), and Kyoto Encyclopedia of Genes and Genomes (KEGG) (**D**) analysis of PLP.

The predicted targets were screened, and 322 potential targets were obtained by removing duplicate values of the identified components. After aggregation and deduplication, 431 genes related to BSS were obtained from the GeneCard database. The disease targets intersected with the potential targets of PLP, and 61 targets were obtained (Figure 6A). Details of the raw data are shown in Tables S4 and S5.

### 2.6. Protein–Protein Interaction (PPI) Network Analysis

Sixty-one targets were input into the STRING database to establish a PPI network. In the network, redder nodes represented larger values (Figure 6B). A median more than twice the measured value was chosen as the basis for selection, and 10 core targets were obtained: Albumin (ALB), AKT serine/threonine Kinase 1 (AKT1), matrix metallopeptidase 9 (MMP9), Proto-oncogene tyrosine-protein kinase Src (SRC), Epidermal Growth Factor Receptor (EGFR), Heat Shock Protein 90 Alpha Family Class A Member 1 (HSP90AA1), Kinase Insert Domain Receptor (KDR), Estrogen Receptor 1 (ESR1), Nitric Oxide Synthase 3 (NOS3), and peroxisome proliferator-activated receptor gamma (PPARG).

Proteins encoded by ALB play a role in regulating plasma colloidal osmotic pressure and can be used as carrier proteins for a variety of endogenous molecules (including hormones, fatty acids, and metabolites) and exogenous drugs. Pathways associated with ALB include heme biosynthesis and responses to elevated platelet cytoplasmic Ca^2+^ [23,24]. AKT1 regulates a variety of cellular functions, including cell proliferation, inventory, metabolism, and angiogenesis [25,26]. The PI3K pathway, which is closely related to AKT1, mediates the effects of various growth factors, including platelet-derived growth factor (PDGF) and epidermal growth factor (EGF) [27,28]. MMP9, SRC, HSP90AA1, EGFR, and ESR1 are involved in cell cycle regulation and control a variety of bioactive signaling pathways [29,30,31,32,33,34]. NOS3 is related to the relaxation of vascular smooth muscle through the CGMP-mediated signal transduction pathway [35], and the NO produced by NOS3 in the vascular endothelium has the function of regulating vascular tension, cell proliferation, leukocyte adhesion, and platelet aggregation [36]. PPAR-γ, which is encoded by PPAGR, is associated with the pathologies of several diseases, including obesity, diabetes, atherosclerosis, and cancer [37]. KDR encodes two receptors for vascular endothelial growth factor (VEGF) that play an important role in the regulation of angiogenesis, vascular development, and vascular permeability [38,39]. These results show that the core targets of PLP play key roles in angiogenesis, coagulation, inflammation, and apoptosis.

### 2.7. Functional Enrichment Analysis and Pathway Enrichment Analysis

To better understand the pharmacological mechanism of PLP in hemorheological abnormalities, we performed a Gene Ontology (GO) enrichment and Kyoto Encyclopedia of Genes and Genomes (KEGG) pathway analysis based on 61 related targets and predicted the top 10 and top 24 KEGG pathways of biological processes (BPs), molecular functions (MFs), and cellular components (CCs). The functionally enriched GO results showed that a total of 194 GO terms were identified (*p* < 0.01), of which 135 were related to BPs (e.g., positive regulation of kinase activity, negative regulation of the apoptotic process, positive regulation of cell proliferation, angiotensin maturation, and angiogenesis), 40 terms were related to MFs (e.g., zinc ion binding, enzyme binding, transmembrane receptor protein tyrosine kinase activity, protein binding, and ATP binding), and 19 terms were related to CCs (e.g., receptor complexes, cell surface, basal plasma membrane, and chromatin) (Figure 6C). The results showed that the main mechanism by which PLP promotes blood circulation and removes blood stasis is to inhibit cell apoptosis, participate in the cellular response to VEGF stimulation, positively regulate the migration of vascular endothelial cells and the proliferation of vascular smooth muscle cells, and promote the remodeling and regeneration of blood vessels. PLP, on the other hand, is involved in angiotensin maturation and regulates the contraction and relaxation of blood vessels.

Next, we used the DAVID database to analyze the enrichment findings from the KEGG pathways. The KEGG pathways of the 61 targets above were analyzed using the DAVID database, and a total of 73 pathways were enriched, of which 36 were statistically significant. The main channels were pathways in cancer, proteoglycans in cancer, the PI3K-Akt signaling pathway, prostate cancer, endocrine resistance, the Relaxin signaling pathway, etc. The first 20 items were selected according to the FDR < 0.01 cutoff (Figure 6D). The results of the KEGG analysis showed that BSS-related targets were related to the PI3K-Akt signaling pathway, fluid shear stress and atherosclerosis, the Rap1 signaling pathway, the MAPK signaling pathway, the HIF-1 signaling pathway, adherens junctions, complement and coagulation cascades, the VEGF signaling pathway, etc. Details of the raw data are shown in Table S6.

Thrombosis consists of insoluble fibrin, deposited platelets, impaired white blood cells, and retained red blood cells, leading to the formation of BSS. The Rap1 signaling pathway and adherens junctions are involved in cell adhesion and lay the foundation for the formation and maintenance of blood vessels [40,41], and the PI3K-Akt signaling pathway also plays a role in promoting angiogenesis. Both the complement and coagulation systems limit pathogen infections through the innate immune mechanism, and an overactive complement may be involved in the formation of a thrombus [42,43]. When platelets play a hemostatic role, they quickly bind to damaged blood vessels, leading to thrombosis [44]. The VEGF signaling pathway is the most significant pathway enriched by PLP promoting blood circulation and removing blood stasis, which suggests that this pathway may be the key mechanism for PLP-induced effects on blood circulation and the removal of blood stasis. The VEGF signaling pathway plays key roles in vasculogenesis and angiogenesis. In neovascularization, VEGF is associated with the induction of gene expression; the regulation of vascular permeability; and the promotion of cell migration, proliferation, and survival. HIF-1 is also involved in the regulation of VEGF [45,46]. Studies have shown that VEGF is related to the extracellular signal-regulated kinase (ERK) 1/2 signaling pathway and that ERK1/2 is involved in the abnormal expression of VEGF [47,48]. BSS is associated with the ERK1/2-VEGF signaling pathway [49], and drugs can reduce the expression of VEGF by affecting the ERK1/2 pathway, resulting in an anti-blood stasis effect [50].

### 2.8. “Component–Target–Pathway” Network Analysis

**Figure 7:**
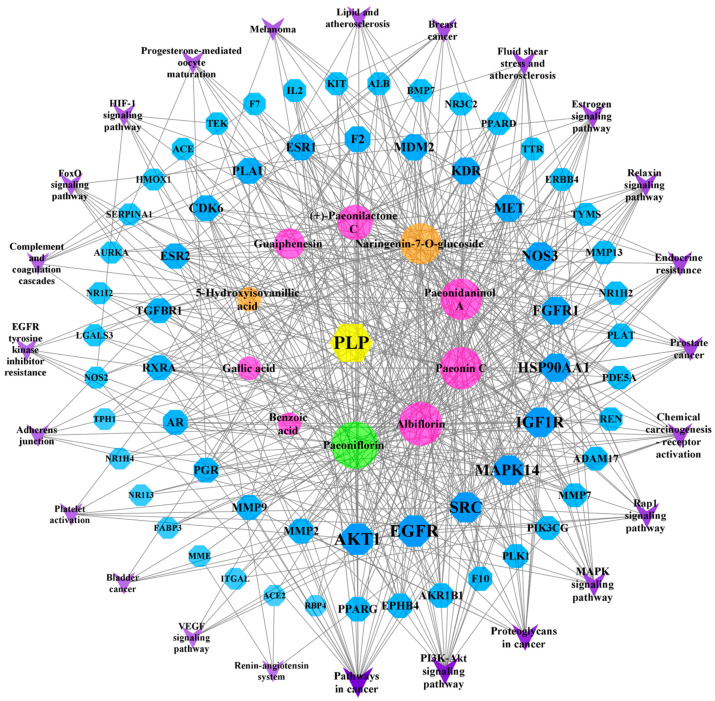
The “component–target–pathway” network.

The 10 components and 61 PLP targets in the treatment of BSS and the 24 possible pathways (FDR < 0.01) identified using the KEGG enrichment method were imported into Cytoscape software (version 3.8.2) to construct a network map (Figure 7). On the map, blue represents a target, purple represents a pathway, pink represents a common component, orange is unique to PRA, green is unique to PRR, and the lines indicate the interactions between these variables. Among the active compounds, Paeoniflorin, Albiflorin, Paeonin C, Paeonidaninol A, Naringenin-7-*O*-glucoside, (+)-Paeonilactone C, Guaiphenesin, 5-Hydroxyisovanillic acid, Gallic acid, and Benzoic acid interact with 57, 49, 47, 45, 43, 32, 22, 8, 7, and 5 targets, respectively. Through the “component–target–pathway” network, we can identify the processes through which the components exert their effects. For example, Paeoniflorin, Paeonin C, and Naringenin-7-*O*-glucoside can act on SERPINA1 and participate in complement and coagulation cascades. All 10 components can be involved in the VEGF signaling pathway through one or more targets, including AKT1, EGFR, SRC, MAPK14, NOS3, and KDR.

### 2.9. Molecular Docking Validation Analysis

The target network of the components was analyzed using Cytoscape software, and components with a degree value greater than the median were selected as the docking ligands. Three-dimensional (3D) files of each component were downloaded from the TCMSP and PubChem databases in the sdf/mol2 formats, and the sdf format was converted to the mol2 format using Openbabel 3.1.1 software.

**Figure 8:**
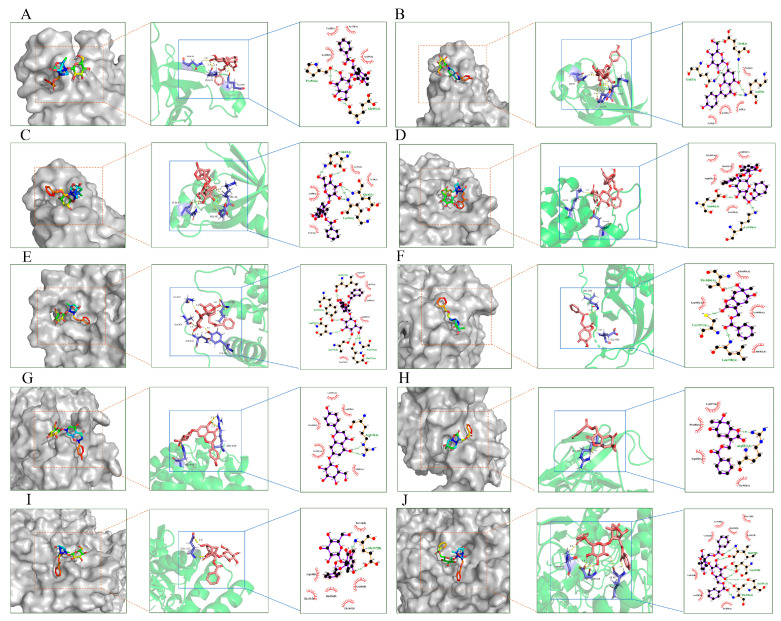
Molecular docking diagrams. AKT1–Albiflorin (**A**), AKT1–Naringenin-7-*O*-glucoside (**B**), ATK1–Paeoniflorin (**C**), EGFR–Paeonidaninol A (**D**), EGFR–Paeoniflorin (**E**), EGFR–(+)-Paeonilactone C (**F**), KDR–Naringenin-7-*O*-glucoside (**G**), KDR–(+)-Paeonilactone C (**H**), NOS3–Albiflorin (**I**), NOS3–PaeonidaninolA (**J**).

The core target was selected using the PPI as the target protein for molecular docking, and the 3D structure of the docking protein was downloaded from the PDB database, saved in the “pdb” format, and imported into PyMOL software (version 2.4.0) to remove water molecules and ligands. Finally, the structure was imported into AutoDockTools 1.5.7 software to hydrogenate, calculate the charge, and set the atomic type of the protein. The selected ligands underwent “Detect Root” and “Choose Torsions” operations, and the pretreated proteins and ligands were used to start the docking analysis after setting the docking range under “Grid” and choosing the minimum docking mode of binding energy. The exported file was converted from the “pdb” format to the “pdbqt” format using OpenBabel 3.1.1 software, and the results were visualized using PyMOL and LigPlot+ software (version 2.2.7). The results are shown in Figure 8.

The results show that Albiflorin with AKT1 and NOS3, Naringenin-7-*O*-glucoside with AKT1 and KDR, Paeoniflorin with AKT1 and EGFR, PaeonidaninolA with EGFR and NOS3, and (+)-Paeonilactone C with EGFR and KDR can bind more closely. The results show that Albiflorin, Paeoniflorin, PaeonidaninolA, (+)-Paeonilactone C, and Naringenin-7-*O*-glucoside, which were predicted for PLP, could play a key role in improving BSS. The mechanism is related to the key targets of AKT1, NOS3, EGFR, and KDR.

In this study, the effect of PLP on the promotion of blood circulation and the removal of blood stasis was verified. This study determined the components of PRA and PRR that enter the blood through the combination of UPLC-Q-TOF-MS technology. Based on the pharmacological analysis of the network, we found that Paeoniflorin, Albiflorin, Paeonin C, Paeonidaninol A, Naringenin-7-*O*-glucoside, (+)-Paeonilactone C, Guaiphenesin, 5-Hydroxyisovanillic acid, Gallic acid, and Benzoic acid are related to the targets ALB, AKT1, EGFR, NOS3, PPARG, VEGF, MMP9, and HAP90AA1. These targets activate the PI3K-Akt signaling pathway, the MAPK signaling pathway, adherens junctions, complement and coagulation cascades, and the VEGF signaling pathway and participate in BPs such as angiogenesis, vasoconstriction and relaxation, coagulation, and the migration and proliferation of vascular cells. They ultimately play a role in the treatment of BSS.

There are many studies on improving BSS using PRR [51], but the results of this study show that PRA can also improve BSS by regulating hemorheological and coagulation function indexes. The study of the serum pharmacochemistry of traditional Chinese medicine shows that the pharmacological effects of oral drugs must depend on the pharmacodynamic substances entering the circulation [52]. Therefore, results obtained by interpreting a mechanism of action using the components entering the blood are more objective and rigorous. In this study, the mechanism of PLP in the treatment of BSS was determined. PLP mainly plays a role by affecting the VEGF signaling pathway, and overexpression of the VEGF signaling pathway can cause diseases such as diabetic retinopathy [50] and macular degeneration [53]. Therefore, PLP may also have a positive effect on such diseases and can provide more treatment ideas.

Given the limitations of network pharmacology, it will be necessary to further verify potential active components, targets, and pathways related to PLP through experiments in the future.

## 3. Materials and Methods

### 3.1. Reagents and Materials

#### 3.1.1. Reagents

Gallic acid (DST190715-008, purity ≥ 98%), Catechin (DST190628-001, purity ≥ 98%), Albiflorin (DST190120-071, purity ≥ 98%), 1,2,3,4,6-pentagalloglucose (DST190517-001, purity ≥ 98%), and benzoic acid (DST190609-001, purity ≥ 98%) were obtained from Lemeitian Pharmaceutical Company (Chengdu, China). Paeoniflorin (X12ABC33672, purity ≥ 98%) and oxypaeoniflorin (P12N9S74762, purity ≥ 98%) were purchased from Shanghai Yuanye Biotechnology Co., Ltd. (Shanghai, China). Acetonitrile (chromatographically pure) was purchased from Oceanpak alexative Chemical Co., Ltd. (Gothenburg, Sweden); phosphoric acid (analytical-grade) and methanol (analytical-grade) were obtained from Sinopharm Chemical Reagent Co., Ltd. (Shanghai, China); and ultra-pure water and distilled water were provided by ultra-pure water devices in the laboratory. Fufang Danshen tablets (FDT) were purchased from Zhengzhou Ruilong Pharmaceutical Co., Ltd. (Zhengzhou, China); enteric-coated aspirin tablets were obtained from Chenxin Pharmaceutical Co., Ltd. (Jining, China); and epinephrine hydrochloride injections were purchased from Jilin Huamu Animal Health Products Co., Ltd. (Changchun, China). Sodium chloride, anhydrous ethanol, and chloral hydrate, all pure analytical-grade reagents, were purchased from Sinopharm Chemical Reagent Co., Ltd. (Shanghai, China). A sodium heparin anticoagulant and sodium citrate anticoagulant blood collection tubes were purchased from Shandong Osite Medical Equipment Co., Ltd. (Chengwu, China).

#### 3.1.2. Plant Materials

*Paeonia lactiflora* Pall. samples were collected in Bozhou in August 2019. All plant samples were identified by Professor Yu Nianjun from Anhui University of Chinese Medicine, referring to the *Flora of China* [54]. 

*Paeoniae Radix* Alba was obtained by boiling, peeling, and drying PLP. *Paeoniae Radix Rubra* was obtained by directly drying PLP, as per the 2020 edition of the Chinese Pharmacopoeia [3].

### 3.2. Animals and Treatment

SPF-grade 6–7-week-old male SD rats weighing 200–250 g were provided by Pizhou Dongfang Culture Co., Ltd. (Pizhou, China) (Laboratory Animal Quality Certificate number: No. 202014282, license number: SCXK (Su) 2017-0003). The rats were housed with a 12 h light/12 h dark cycle, a controlled room temperature between 21 and 23 °C, and free access to water. All experimental procedures were performed according to protocols approved by the Animal Care and Use Committee of Anhui University of Chinese Medicine. All animal experiments were performed according to institutional guidelines for ethical animal studies.

### 3.3. Herbal Preparation

First, 10.8 g of medicinal powder (filtered through a No. 4 sieve) was accurately weighed and placed in a 250 mL flask. Then, 108 mL of distilled water (10 times the volume) was added, and the solution was heated in a water bath at 100 °C in two phases: the first was for 1 h and the second was for 0.5 h. The solution was then cooled and centrifuged at 4 °C and 2000 r/min for 10 min, and the resulting supernatant was combined, evaporated to dryness in a water bath at 100 °C, dissolved in distilled water, and diluted to a volume of 100 mL to obtain a 0.108 g/mL solution based on the crude drug amount. One part of the prepared solution was used for analytical detection, and the other part was used for a gavage treatment. 

### 3.4. Experimental Model and Drug Administration

Thirty-six male SD rats were randomly divided into six groups (*n* = 6) as follows: a control group, a model group, an aspirin group (27 mg/kg), a Fufang Danshen tablet group (FDT, 2.7 g/kg), a PRA group (1.08 g/kg), and a PRR group (1.08 g/kg). The rats in each group received the corresponding drugs via intragastric administration, while those in the control group received normal saline once a day. Prophylactic administration was carried out from day 1 to day 3, and the drugs were administered from day 14 to day 23. Blood samples were taken on days 13 and 23 from the tail veins of the rats in each group to detect the bleeding and clotting times.

Except for the control group, the rats in the other five groups were subcutaneously injected with epinephrine hydrochloride (0.4 mg/kg) twice a day from day 4 to day 23, while the rats in the control group were subcutaneously injected with the same amount of normal saline twice a day. The injections occurred at intervals of 4 h and 2 h after the first subcutaneous injection, followed by an ice water bath at 0 °C for 5 min. Then, the back hair was dried. After modeling on day 23, all rats were weighed after fasting for 12 h, and a gavage was administered. After administering the gavage for 1.5 h, 2.5% pentobarbital sodium (1 mL/kg) anesthetic was injected intraperitoneally, and blood was taken from the abdominal aorta. The blood was placed in a centrifuge tube containing an anticoagulant and centrifuged to collect plasma. All plasma samples in a group were combined into one sample and analyzed [55].

### 3.5. Hemorheological and Coagulation Parameters

Blood from the abdominal aortas of the experimental rats was placed in a sodium heparin anticoagulation tube and a sodium citrate anticoagulation tube, respectively, to detect hemorheological indexes such as WBV, EAI, PV, ESR, and HCT and coagulation function indexes such as PT, APTT, FBG, and TT [56].

### 3.6. Detection and Identification of Blood Components

#### 3.6.1. Preparation of Medicated and Blank Sera

Blood was drawn from the abdominal aortas of the rats and centrifuged at 10,000 rpm for 10 min at 4 °C, and the supernatant was obtained. Methanol was added, and the mixture was centrifuged again at 10,000 rpm for 10 min at 4 °C. The supernatant was then dried under nitrogen stream, and the residue was re-suspended with 400 μL of 75% methanol. The samples were centrifuged at 10,000 rpm for 10 min at 4 °C, and the supernatant was collected and filtered through a syringe filter (0.22 µm). A volume of 10 µL was injected into a UPLC-Q-TOF-MS system for analysis [57].

#### 3.6.2. Chromatographic Conditions

The chromatographic system used was a Shimadzu Nexera UHPLC LC-30A ultra-high-performance liquid chromatography system. Chromatographic separation was conducted on a Waters UPLC BEH C18 column (1.7 μm × 2.1 × 100 mm). The injection volume was 5 μL. The mobile phase consisted of mobile phase A (0.1% formic acid aqueous solution) and mobile phase B (0.1% formic acid–acetonitrile solution). The gradient elution procedure for the analysis was as follows: ~0–3.5 min, 5% B→15% B; ~3.5–6.0 min, 15% B→30% B; ~6.0–6.5 min, 30% B→30% B; ~6.5–12.0 min, 30% B→70% B; ~12.0–12.5 min, 70% B→70% B; ~12.5–18.0 min, 70% B→100% B; and ~18.0–25.0 min, 100% B→100% B [58].

#### 3.6.3. MS Conditions

Mass spectrometry analysis was conducted on an AB5600 + TripleTOF mass spectrometer (workstation Analyst TF1.7.1, AB Sciex), and the IDA function was used to collect first- and second-stage mass spectrometry data. In each data acquisition cycle, ions with an intensity greater than 100 were selected for the corresponding secondary mass spectrometry data acquisition. The optimal conditions of the mass spectrum parameters were set as follows: decluttering voltage (DP): 100 eV (−100 eV), first-order bombardment energy: 10 eV (−10 eV), second-order bombardment energy (CE): 40 eV (−40 eV), collision energy difference (CES): 20 V, 15 secondary spectrum acquisition intervals of 50 ms, atomization pressure (GS1): 55 Psi, auxiliary pressure: 55 Psi, curtain gas: 35 Psi, temperature: 550 °C, and spray voltage: 5500 V (positive ion mode) or −4000 V (negative ion mode) [58].

#### 3.6.4. Data Processing and Compound Identification

The collected data (MS and MS/MS data collected in the IDA mode) were imported into Progenesis QI software (version 2.0), and the corresponding loading programs were selected (POS: +H, + NH_4_, + Na, and + H-H_2_O; NEG: -H, -H-H_2_O, and + FA-H). The time range was set to 0.5–25 min. The molecular weight and fragment mass deviations were set to 5 ppm and 10 ppm, respectively. Peak matching, alignment, and extraction were based on retention time and mass number, and the matrix data contained each retention time and mass number. The corresponding peak intensities were obtained. Next, the secondary mass spectrometry database (AB Sciex commercial database: TCM_2.0_MSMSLibrary, Allinone, NIST) was queried to match the collected MS/MS data and to obtain information on the cracking laws of various compounds.

Finally, Scifinder, the “Collection of original plant chemical components of traditional Chinese medicine”, and other related documents and books were queried using the Latin name as an index word to obtain relevant chemical composition information, including the name, molecular formula, exact mass number, and other related information. The SDF composition was established using Progenesis SDF Studio software (version 1.0), and the MS and MS/MS fragments were analyzed using Progenesis QI software combined with the SDF database. The matching results were analyzed according to previous information on the cracking laws of various compounds to confirm the names of the compounds to be verified. Components in the library with similarity values lower than 80 (maximum 100) were not identified [59,60].

### 3.7. Network Pharmacology Analysis

#### 3.7.1. Active Components and Targets

Based on the results of a UPLC-Q-TOF-MS analysis of the serum samples, the metabolic components in the serum were selected to predict the biological targets. First, the structures of the target components were obtained from the National Library of Medicine (https://pubchem.ncbi.nlm.nih.gov (accessed on 30 January 2023)) and J-GLOBAL (https://jglobal.jst.go.jp/en (accessed on 30 January 2023)), downloaded in “Sdf” format, and uploaded to the PharmMapper database [61,62,63] (http://www.lilab-ecust.cn/pharmmapper/ (accessed on 30 January 2023)) to predict the targets. Targets with scores greater than 0.5 and gene annotations were determined using the UniProt database (https://www.uniprot.org/) (accessed on 30 January 2023). Genes related to BSS-related diseases were searched in the GeneCard (https://www.genecards.org/ (accessed on 30 January 2023)) and OMIM databases (https://www.omim.org/ (accessed on 30 January 2023)). The intersections of the drug and disease targets were selected as the PLP targets for BSS [64,65,66].

#### 3.7.2. Construction of Protein–Protein Interaction Networks

The common targets of PRA-PRR and BSS were imported into the STRING database (https://string-db.org/ (accessed on 30 January 2023)). Species were limited to “Homo Sapiens”, and the interactions were identified. The above data were imported into Cytoscape 3.9.1 software to build a target PPI network diagram. In addition, the Molecular Complex Detection (MCODE) plugin in Cytoscape version 3.8.2 was used to screen the modules in the PPI network in Cytoscape (degree cutoff = 2, node score cutoff = 0.2, k-core = 2, and max. depth = 100) [67,68].

#### 3.7.3. Gene Ontology and Kyoto Encyclopedia of Genes and Genomes Analyses

Potential targets for the treatment of BSS were entered into the DAVID database (https://david.ncifcrf.gov/ (accessed on 30 January 2023)) and GO and KEGG pathways for enrichment analyses [69]. GO analysis was then used to annotate gene function via three modules: BPs, MFs, and CCs. We also used the Bioinformatics (http://www.bioinformatics.com.cn/ (accessed on 30 January 2023)) platform to create a bar graph to illustrate the results of the GO enrichment analysis and a bubble chart to describe the results of the KEGG enrichment analysis [70,71].

#### 3.7.4. Construction of the ‘Component–Target–Pathway’ Network

Using Cytoscape software, a “component–target–pathway” network was created. It could visually reflect the relationships between drugs and diseases [68].

#### 3.7.5. Molecular Docking

The 3D structures of the active components were downloaded from PubChem, and the 3D structures of the candidate targets of BSS were downloaded from the Protein Data Bank (https://www.rcsb.org/ (accessed on 30 January 2023)). The structures of the targets were modified by removing ligands, water, and hydrogen by importing them into PyMOL 2.4.0 software. Using AutoDock 4.0 software, candidate pharmacodynamic components and candidate targets were used for docking studies after preprocessing, and the docking results were visualized using PyMol and LigPlot+ v.2.2.7 [72,73].

### 3.8. Statistical Analysis

Data are expressed as the mean ± SEM from at least three independent experiments. One-way analysis of variance (ANOVA) followed by Tukey’s post hoc test was performed to analyze the differences among multiple groups using GraphPad Prism (version 8; GraphPad Software Inc., San Diego, CA, USA). Student *t*-test was used to analyze the difference between the two groups. A *p*-value < 0.05 was considered statistically significant.

## 4. Conclusions

In this study, we demonstrated that PLP is effective in the treatment of BSS and that its potential pharmacological mechanisms may be related to angiogenesis, vasodilation, and platelet activation and aggregation. This study laid a foundation for further research on PLP to promote blood circulation and remove blood stasis.

## Figures and Tables

**Table 1:** Identification of blood components after oral administration of PRA and PRR extracts.

No.	t_R_ (min)	Formula	Error (ppm)	Ion Mode	*m/z*	Fragment Ions (*m/z*)	PRA	PRR	Identification	References
1	5.98	C_21_H_22_O_10_	−2.72	M + H, M + Na	434.1918	375.1155, 104.1070, 86.0974	+	−	Naringenin-7-*O*-glucoside	[14]
2	2.26	C_10_H_14_O_4_	−0.62	M + H-H_2_O, M + Na	181.0857	152.0656, 135.0864, 77.0362	+	+	Guaiphenesin	[15]
3	3.91	C_23_H_28_O_11_	−0.39	M + H, M + Na	503.1152	327.0864	+	+	Albiflorin	[16,17]
4	2.68	C_23_H_28_O_12_	−1.22	M + Na	519.1511	281.0583	+	+	Paeonin C	[18,19]
5	8.05	C_30_H_32_O_12_	0.28	M + Na	607.3274	607.3274	+	+	Paeonidaninol A	[20]
6	3.93	C_17_H_18_O_6_	1.01	M + H-H_2_O, M + H	319.1076	111.0189, 209.0936, 93.0094	+	+	(+)-Paeonilactone C	[14]
7	5.63	C_7_H_6_O_2_	−4.09	M + H	123.0428	81.9458, 79.0622, 77.0409, 51.0235, 50.0160	+	+	Benzoic acid	[21]
8	1.00	C_7_H_6_O_5_	−3.18	M-H	169.0137	124.0148, 97.0048, 69.0105	+	+	Gallic acid	[16,21]
9	2.03	C_8_H_8_O_5_	−2.46	M-H	183.0049	123.0103, 109.0167, 95.0118	+	−	5-Hydroxyisovanillic acid	[22]
10	5.61	C_23_H_28_O_11_	−1.62	M + H-H_2_O, M + Na	503.3051	440.3152, 358.2698, 295.0411, 221.1381, 177.1152, 133.0888, 89.0602	−	+	Paeoniflorin	[16,17]

Note: The "−" and "+" in the PRA and PRR columns of the table represent the presence or absence of the corre-sponding ingredient.

## Data Availability

The data presented in this study are available on request from the corresponding author.

## Supplementary Materials

The following supporting information can be downloaded at: https://www.mdpi.com/article/10.3390/molecules29133019/s1, Table S1. Bleeding and clotting times on days 13 and 23; Table S2. Whole blood viscosity (WBV); Table S3. Hemorheology and blood coagulation indexes; Table S4. The targets of the blood components derived from the PharmMapper Database; Table S5. The disease targets obtained from Genecards and OMIM databases; Table S6. The functional and pathway enrichment analysis results.
