# Supplementary material for: Integrating UPLC-Q-TOF-MS and Network Pharmacology to Explore the Potential Mechanisms of Paeonia lactiflora Pall. in the Treatment of Blood Stasis Syndrome
Source: Molecules. 2024 Jun 26;29(13):3019. doi: 10.3390/molecules29133019 (PMC11243510; doi:10.3390/molecules29133019)
Supplement: Supplementary file 1 [file molecules-29-03019-s001.zip › Table S4 The targets of the blood components derived from the PharmMapper Database.pdf]

Table S4 The targets of the blood components derived from the PharmMapper Database

Component	Norm Fit	Target name	Uniplot（Entry Name)	GENE SYMBOL
Benzoic acid	0.8977	Carbonic anhydrase 2	CAH2_HUMAN	CA2
Benzoic acid	0.8732	Cholinesterase	CHLE_HUMAN	BCHE
Benzoic acid	0.7884	Cell division protein kinase 2	P24941	CDK2
Benzoic acid	0.7638	Aldose reductase	ALDR_HUMAN	AKR1B1
Benzoic acid	0.7042	Glycine amidinotransferase, mitochondrial	GATM_HUMAN	GATM
Benzoic acid	0.6112	Branched-chain-amino-acid aminotransferase, mitochondrial	O15382	BCAT2
Benzoic acid	0.6025	Nitric oxide synthase, endothelial	NOS3_HUMAN	NOS3
Benzoic acid	0.5973	Cyclin-A2	CCNA2_HUMAN	CCNA2
Benzoic acid	0.5671	Prothrombin	THRB_HUMAN	F2
Benzoic acid	0.5423	Serine/threonine-protein kinase Chk1	CHK1_HUMAN	CHEK1
Benzoic acid	0.5411	Arginase-1	ARGI1_HUMAN	ARG1
Benzoic acid	0.5297	Angiotensin-converting enzyme	ACE_HUMAN	ACE
Benzoic acid	0.5285	Mitogen-activated protein kinase 14	Q16539	MAPK14
Benzoic acid	0.5243	Cytochrome P450 2C9	CP2C9_HUMAN	CYP2C9
Gallic acid	0.9493	Probable ATP-dependent RNA helicase DDX6	DDX6_HUMAN	DDX6
Gallic acid	0.9037	Proto-oncogene serine/threonine-protein kinase Pim-1	PIM1_HUMAN	PIM1
Gallic acid	0.8227	Heat shock cognate 71 kDa protein	P11142	HSPA8
Gallic acid	0.8194	cAMP-specific 3,5-cyclic phosphodiesterase 4B	PDE4B_HUMAN	PDE4B
Gallic acid	0.7746	Inositol monophosphatase	IMPA1_HUMAN	IMPA1
Gallic acid	0.7465	Neutrophil gelatinase-associated lipocalin	NGAL_HUMAN	LCN2
Gallic acid	0.738	Beta-secretase 1	BACE1_HUMAN	BACE1
Gallic acid	0.7325	Phenylalanine-4-hydroxylase	PH4H_HUMAN	PAH
Gallic acid	0.7299	Carbonic anhydrase 1	CAH1_HUMAN	CA1
Gallic acid	0.7255	Purine nucleoside phosphorylase	PNPH_HUMAN	PNP
Gallic acid	0.7158	NAD-dependent malic enzyme, mitochondrial	MAOM_HUMAN	ME2
Gallic acid	0.7028	Angiogenin	ANGI_HUMAN	ANG
Gallic acid	0.6783	Glycogen phosphorylase, liver form	P06737	PYGL
Gallic acid	0.6748	Dihydrofolate reductase	DYR_HUMAN	DHFR
Gallic acid	0.6672	Complement factor B	CFAB_HUMAN	CFB
Gallic acid	0.6646	cAMP-specific 3,5-cyclic phosphodiesterase 4D	PDE4D_HUMAN	PDE4D
Gallic acid	0.6623	Cell division protein kinase 2	P24941	CDK2
Gallic acid	0.6072	Tyrosine-protein phosphatase non-receptor type 1	PTN1_HUMAN	PTPN1
Gallic acid	0.5969	Carboxypeptidase B	P15086	CPB1
Gallic acid	0.5931	Kinesin heavy chain	P33176	KIF5B
Gallic acid	0.5928	Serum amyloid P-component	P02743	APCS
Gallic acid	0.5893	Galactokinase	GALK1_HUMAN	GALK1
Gallic acid	0.5864	Hexokinase-1	HXK1_HUMAN	HK1
Gallic acid	0.5838	Cyclin-A2	CCNA2_HUMAN	CCNA2
Gallic acid	0.5827	Urokinase-type plasminogen activator	UROK_HUMAN	PLAU

Gallic acid	0.5813	Pancreatic alpha-amylase	AMYP_HUMAN	AMY2A
Gallic acid	0.5811	Ribonuclease 4	P34096	RNASE4
Gallic acid	0.5736	Heat shock protein HSP 90-alpha	HS90A_HUMAN	HSP90AA1
Gallic acid	0.5706	Activated CDC42 kinase 1	ACK1_HUMAN	TNK2
Gallic acid	0.5693	Dipeptidyl peptidase 4	DPP4_HUMAN	DPP4
Gallic acid	0.5659	Ornithine carbamoyltransferase, mitochondrial	P00480	OTC
Gallic acid	0.5632	Interferon-stimulated gene 20 kDa protein	ISG20_HUMAN	ISG20
Gallic acid	0.5603	Cytidine deaminase	CDD_HUMAN	CDA
Gallic acid	0.557	Inosine-5-monophosphate dehydrogenase 2	IMDH2_HUMAN	IMPDH2
Gallic acid	0.556	Complement component C8 gamma chain	P07360	C8G
Gallic acid	0.5497	Carbonyl reductase [NADPH] 1	CBR1_HUMAN	CBR1
Gallic acid	0.5495	Copper transport protein ATOX1	O00244	ATOX1
Gallic acid	0.548	Adenosylhomocysteinase	SAHH_HUMAN	AHCY
Gallic acid	0.5451	Transforming protein RhoA	P61586	RHOA
Gallic acid	0.5434	Matrilysin	MMP7_HUMAN	MMP7
Gallic acid	0.542	Galactosylgalactosylxylosylprotein 3-beta-glucuronosyltransferase 1	B3GA1_HUMAN	B3GAT1
Gallic acid	0.5405	Mineralocorticoid receptor	MCR_HUMAN	NR3C2
Gallic acid	0.5353	Glycogen synthase kinase-3 beta	GSK3B_HUMAN	GSK3B
Gallic acid	0.5252	L-lactate dehydrogenase B chain	P07195	LDHB
Gallic acid	0.5252	Ephrin type-B receptor 4	EPHB4_HUMAN	EPHB4
Gallic acid	0.5227	Proto-oncogene tyrosine-protein kinase Src	SRC_HUMAN	SRC
Gallic acid	0.5088	Cell division protein kinase 6	CDK6_HUMAN	CDK6
Albiflorin	0.9973	Transthyretin	TTHY_HUMAN	TTR
Albiflorin	0.9969	Glutathione S-transferase P	GSTP1_HUMAN	GSTP1
Albiflorin	0.996	Ephrin type-B receptor 4	EPHB4_HUMAN	EPHB4
Albiflorin	0.9911	Mitogen-activated protein kinase 1	MK01_HUMAN	MAPK1
Albiflorin	0.984	Carbonic anhydrase 2	CAH2_HUMAN	CA2
Albiflorin	0.9837	Mitogen-activated protein kinase 8	MK08_HUMAN	MAPK8
Albiflorin	0.9836	Proto-oncogene serine/threonine-protein kinase Pim-1	P11309	PIM1
Albiflorin	0.9836	Aldose reductase	ALDR_HUMAN	AKR1B1
Albiflorin	0.9831	Proto-oncogene tyrosine-protein kinase Src	SRC_HUMAN	SRC
Albiflorin	0.9826	Reticulon-4 receptor	RTN4R_HUMAN	RTN4R
Albiflorin	0.9805	ADP-ribose pyrophosphatase, mitochondrial	Q9BW91	NUDT9
Albiflorin	0.9796	Mitogen-activated protein kinase 10	MK10_HUMAN	MAPK10
Albiflorin	0.9769	Progesterone receptor	PRGR_HUMAN	PGR
Albiflorin	0.9745	Caspase-7	CASP7_HUMAN	CASP7
Albiflorin	0.9731	Peroxisome proliferator-activated receptor gamma	PPARG_HUMAN	PPARG
Albiflorin	0.9724	Epidermal growth factor receptor	EGFR_HUMAN	EGFR
Albiflorin	0.9724	Purine nucleoside phosphorylase	PNPH_HUMAN	PNP
Albiflorin	0.9724	Methionine aminopeptidase 1	AMPM1_HUMAN	METAP1
Albiflorin	0.9696	Cathepsin S	CATS_HUMAN	CTSS

Albiflorin	0.9672	Heat shock protein HSP 90-alpha	HS90A_HUMAN	HSP90AA1
Albiflorin	0.964	Cholinesterase	CHLE_HUMAN	BCHE
Albiflorin	0.9633	Macrophage migration inhibitory factor	MIF_HUMAN	MIF
Albiflorin	0.9619	Estrogen receptor	ESR1_HUMAN	ESR1
Albiflorin	0.9581	Dihydrofolate reductase	DYR_HUMAN	DHFR
Albiflorin	0.9563	Mitogen-activated protein kinase 14	Q16539	MAPK14
Albiflorin	0.9554	Peptidyl-prolyl cis-trans isomerase FKBP1A	FKB1A_HUMAN	FKBP1A
Albiflorin	0.9541	Bile salt sulfotransferase	Q06520	SULT2A1
Albiflorin	0.9534	Cathepsin D	CATD_HUMAN	CTSD
Albiflorin	0.9527	cAMP-specific 3,5-cyclic phosphodiesterase 4D	PDE4D_HUMAN	PDE4D
Albiflorin	0.9525	Caspase-3	CASP3_HUMAN	CASP3
Albiflorin	0.9522	cAMP-specific 3,5-cyclic phosphodiesterase 4B	PDE4B_HUMAN	PDE4B
Albiflorin	0.9518	NAD(P)H dehydrogenase [quinone] 1	NQO1_HUMAN	NQO1
Albiflorin	0.9516	Peptidyl-prolyl cis-trans isomerase A	P62937	PPIA
Albiflorin	0.9508	Serine/threonine-protein kinase Chk1	CHK1_HUMAN	CHEK1
Albiflorin	0.9493	Complement factor D	CFAD_HUMAN	CFD
Albiflorin	0.9479	Probable ATP-dependent RNA helicase DDX6	DDX6_HUMAN	DDX6
Albiflorin	0.9453	TGF-beta receptor type-2	TGFR2_HUMAN	TGFBR2
Albiflorin	0.9439	Triggering receptor expressed on myeloid cells 1	Q9NP99	TREM1
Albiflorin	0.9428	Beta-secretase 1	BACE1_HUMAN	BACE1
Albiflorin	0.9347	TGF-beta receptor type-1	TGFR1_HUMAN	TGFBR1
Albiflorin	0.9311	Vascular endothelial growth factor receptor 2	VGFR2_HUMAN	KDR
Albiflorin	0.9307	Cyclin-A2	CCNA2_HUMAN	CCNA2
Albiflorin	0.9282	Inositol monophosphatase	IMPA1_HUMAN	IMPA1
Albiflorin	0.9251	Peroxisome proliferator-activated receptor delta	Q03181	PPARD
Albiflorin	0.9248	Seprase	SEPR_HUMAN	FAP
Albiflorin	0.9246	Cyclin-dependent kinase 5 activator 1	CD5R1_HUMAN	CDK5R1
Albiflorin	0.9245	Carbonic anhydrase 1	CAH1_HUMAN	CA1
Albiflorin	0.9213	MAP kinase-activated protein kinase 2	P49137	MAPKAPK2
Albiflorin	0.9181	Basic fibroblast growth factor receptor 1	FGFR1_HUMAN	FGFR1
Albiflorin	0.9129	Carbonic anhydrase 12	CAH12_HUMAN	CA12
Albiflorin	0.91	3-phosphoinositide-dependent protein kinase 1	PDPK1_HUMAN	PDPK1
Albiflorin	0.9078	Insulin-like growth factor 1 receptor	P08069	IGF1R
Albiflorin	0.9009	Estrogen-related receptor gamma	P62508	ESRRG
Albiflorin	0.8884	Oxysterols receptor LXR-beta	NR1H2_HUMAN	NR1H2
Albiflorin	0.8848	S-methyl-5-thioadenosine phosphorylase	Q13126	MTAP
Albiflorin	0.879	Sex hormone-binding globulin	SHBG_HUMAN	SHBG
Albiflorin	0.8779	Stromelysin-1	MMP3_HUMAN	MMP3
Albiflorin	0.8744	Collagenase 3	MMP13_HUMAN	MMP13
Albiflorin	0.8705	Retinoic acid receptor RXR-alpha	RXRA_HUMAN	RXRA
Albiflorin	0.8637	Ribosyldihydronicotinamide dehydrogenase [quinone]	P16083	NQO2
Albiflorin	0.862	Chymase	CMA1_HUMAN	CMA1

Albiflorin	0.8562	Phosphatidylinositol-4,5-bisphosphate 3-kinase catalytic subunit gamma isoform	PK3CG_HUMAN	PIK3CG
Albiflorin	0.8556	Sorbitol dehydrogenase	DHSO_HUMAN	SORD
Albiflorin	0.8555	Heat shock cognate 71 kDa protein	P11142	HSPA8
Albiflorin	0.8542	Amine oxidase [flavin-containing] B	AOFB_HUMAN	MAOB
Albiflorin	0.8495	Complement factor B	CFAB_HUMAN	CFB
Albiflorin	0.8409	Cell division protein kinase 2	P24941	CDK2
Albiflorin	0.8327	Annexin A5	ANXA5_HUMAN	ANXA5
Albiflorin	0.818	Prothrombin	THRB_HUMAN	F2
Albiflorin	0.8153	Cytochrome P450 2C9	CP2C9_HUMAN	CYP2C9
Albiflorin	0.8114	Androgen receptor	ANDR_HUMAN	AR
Albiflorin	0.7991	Galectin-7	LEG7_HUMAN	LGALS7
Albiflorin	0.7915	Angiogenin	ANGI_HUMAN	ANG
Albiflorin	0.7855	Neutrophil collagenase	MMP8_HUMAN	MMP8
Albiflorin	0.7832	Kinesin-like protein KIF11	KIF11_HUMAN	KIF11
Albiflorin	0.7803	Superoxide dismutase [Mn], mitochondrial	P04179	SOD2
Albiflorin	0.7777	Epoxide hydrolase 2	HYES_HUMAN	EPHX2
Albiflorin	0.7759	Coagulation factor X	P00742	F10
Albiflorin	0.7751	Hexokinase-1	HXK1_HUMAN	HK1
Albiflorin	0.7746	Estradiol 17-beta-dehydrogenase 1	P14061	HSD17B1
Albiflorin	0.7733	Troponin C, slow skeletal and cardiac muscles	P63316	TNNC1
Albiflorin	0.7695	Corticosteroid 11-beta-dehydrogenase isozyme 1	DHI1_HUMAN	HSD11B1
Albiflorin	0.7687	Phospholipase A2, membrane associated	P14555	PLA2G2A
Albiflorin	0.7674	Poly [ADP-ribose] polymerase 1	P09874	PARP1
Albiflorin	0.7672	Mineralocorticoid receptor	MCR_HUMAN	NR3C2
Albiflorin	0.7669	Fibroblast growth factor receptor 2	P21802	FGFR2
Albiflorin	0.7656	Serum albumin	ALBU_HUMAN	ALB
Albiflorin	0.7649	Cathepsin K	CATK_HUMAN	CTSK
Albiflorin	0.7645	Carbonyl reductase [NADPH] 1	CBR1_HUMAN	CBR1
Albiflorin	0.7644	Calmodulin	CALM_HUMAN	CALM
Albiflorin	0.764	cGMP-inhibited 3,5-cyclic phosphodiesterase B	PDE3B_HUMAN	PDE3B
Albiflorin	0.763	Tyrosine-protein kinase SYK	KSYK_HUMAN	SYK
Albiflorin	0.7629	Proto-oncogene tyrosine-protein kinase LCK	LCK_HUMAN	LCK
Albiflorin	0.7616	E3 ubiquitin-protein ligase Mdm2	MDM2_HUMAN	MDM2
Albiflorin	0.761	Aldo-keto reductase family 1 member C1	Q04828	AKR1C1
Albiflorin	0.7609	Glycogen synthase kinase-3 beta	P49841	GSK3B
Albiflorin	0.7587	Dipeptidyl peptidase 4	DPP4_HUMAN	DPP4
Albiflorin	0.7556	Serine/threonine-protein kinase PAK 7	PAK7_HUMAN	PAK7
Albiflorin	0.7528	Death-associated protein kinase 1	DAPK1_HUMAN	DAPK1
Albiflorin	0.7507	Glycogen phosphorylase, liver form	P06737	PYGL
Albiflorin	0.7505	Catenin alpha-1	CTNA1_HUMAN	CTNNA1
Albiflorin	0.75	cAMP-dependent protein kinase catalytic subunit alpha	P00517	PRKACA
Albiflorin	0.7472	Casein kinase II subunit alpha	P68400	CSNK2A1

Albiflorin	0.7457	Nuclear receptor ROR-alpha	RORA_HUMAN	RORA
Albiflorin	0.7438	3-hydroxy-3-methylglutaryl-coenzyme A reductase	HMDH_HUMAN	HMGCR
Albiflorin	0.7411	Alcohol dehydrogenase 1B	ADH1B_HUMAN	ADH1B
Albiflorin	0.7398	Deoxycytidine kinase	DCK_HUMAN	DCK
Albiflorin	0.7379	Liver carboxylesterase 1	EST1_HUMAN	CES1
Albiflorin	0.7376	Activated CDC42 kinase 1	ACK1_HUMAN	TNK2
Albiflorin	0.7364	Alcohol dehydrogenase 1C	P00326	ADH1C
Albiflorin	0.7355	cGMP-specific 3,5-cyclic phosphodiesterase	PDE5A_HUMAN	PDE5A
Albiflorin	0.7349	Group 10 secretory phospholipase A2	O15496	PLA2G10
Albiflorin	0.732	Renin	RENI_HUMAN	REN
Albiflorin	0.7314	Cathepsin B	CATB_HUMAN	CTSB
Albiflorin	0.7287	Phenylalanine-4-hydroxylase	PH4H_HUMAN	PAH
Albiflorin	0.7285	Cell division protein kinase 6	CDK6_HUMAN	CDK6
Albiflorin	0.7279	Tyrosyl-tRNA synthetase, cytoplasmic	P54577	YARS1
Albiflorin	0.7268	Macrophage metalloelastase	MMP12_HUMAN	MMP12
Albiflorin	0.7262	Estrogen receptor beta	Q62986	ESR2
Albiflorin	0.7245	Ig lambda chain V-II region MGC	P01709	IGLV2-8
Albiflorin	0.7233	C-1-tetrahydrofolate synthase, cytoplasmic	P11586	MTHFD1
Albiflorin	0.7213	Pyridoxine-5-phosphate oxidase	Q9NVS9	PNPO
Albiflorin	0.7198	Ras-related C3 botulinum toxin substrate 2	P15153	RAC2
Albiflorin	0.7173	Urokinase-type plasminogen activator	UROK_HUMAN	PLAU
Albiflorin	0.7154	Tyrosine-protein phosphatase non-receptor type 1	PTN1_HUMAN	PTPN1
Albiflorin	0.7133	Histone deacetylase 8	Q9BY41	HDAC8
Albiflorin	0.7124	Bone morphogenetic protein 7	BMP7_HUMAN	BMP7
Albiflorin	0.7114	Casein kinase I isoform gamma-2	KC1G2_HUMAN	CSNK1G2
Albiflorin	0.7038	Protein farnesyltransferase/geranylgeranyltransferase type-1 subunit alpha	FNTA_HUMAN	FNTA
Albiflorin	0.7029	Tyrosine-protein phosphatase non-receptor type 11	PTN11_HUMAN	PTPN11
Albiflorin	0.702	Aldo-keto reductase family 1 member C2	AK1C2_HUMAN	AKR1C2
Albiflorin	0.7007	Aldo-keto reductase family 1 member C3	AK1C3_HUMAN	AKR1C3
Albiflorin	0.6943	Hepatocyte growth factor receptor	P08581	MET
Albiflorin	0.6943	Carnitine O-acetyltransferase	CACP_HUMAN	CRAT
Albiflorin	0.6927	SEC14-like protein 2	O76054	SEC14L2
Albiflorin	0.6901	Chitotriosidase-1	Q13231	CHIT1
Albiflorin	0.6896	[Pyruvate dehydrogenase [lipoamide]] kinase isozyme 2, mitochondrial	PDK2_HUMAN	PDK2
Albiflorin	0.6896	Wiskott-Aldrich syndrome protein	WASP_HUMAN	WAS
Albiflorin	0.6848	Coagulation factor VII	FA7_HUMAN	F7
Albiflorin	0.6837	Zinc-alpha-2-glycoprotein	ZA2G_HUMAN	AZGP1
Albiflorin	0.6792	Complement C1r subcomponent	P00736	C1R
Albiflorin	0.6774	Methionine aminopeptidase 2	AMPM2_HUMAN	METAP2
Albiflorin	0.6773	Adenosylhomocysteinase	SAHH_HUMAN	AHCY
Albiflorin	0.6737	Branched-chain-amino-acid aminotransferase, mitochondrial	O15382	BCAT2
Albiflorin	0.671	Lanosterol synthase	ERG7_HUMAN	LSS

Albiflorin	0.6674	Galectin-2	LEG2_HUMAN	LGALS2
Albiflorin	0.6634	BAG family molecular chaperone regulator 1	BAG1_HUMAN	BAG1
Albiflorin	0.6592	Histo-blood group ABO system transferase	P16442	ABO
Albiflorin	0.654	Glucosamine-6-phosphate isomerase	P46926	GNPDA1
Albiflorin	0.6513	Ganglioside GM2 activator	P17900	GM2A
Albiflorin	0.6506	Tyrosine-protein kinase HCK	HCK_HUMAN	HCK
Albiflorin	0.6495	Pancreatic alpha-amylase	AMYP_HUMAN	AMY2A
Albiflorin	0.6494	Galactosylgalactosylxylosylprotein 3-beta-glucuronosyltransferase 1	B3GA1_HUMAN	B3GAT1
Albiflorin	0.6468	Lithostathine-1-alpha	P05451	REG1A
Albiflorin	0.6466	Leukocyte elastase	ELNE_HUMAN	ELANE
Albiflorin	0.6433	Oxysterols receptor LXR-alpha	NR1H3_HUMAN	NR1H3
Albiflorin	0.6415	Serine/threonine-protein kinase PLK1	P53350	PLK1
Albiflorin	0.6412	Dihydroorotate dehydrogenase, mitochondrial	PYRD_HUMAN	DHODH
Albiflorin	0.641	Thymidylate synthase	TYSY_HUMAN	TYMS
Albiflorin	0.6408	GTP-binding protein Rheb	Q15382	RHEB
Albiflorin	0.6407	Thyroid hormone receptor beta	P10828	THRB
Albiflorin	0.6319	Tyrosine-protein kinase ITK/TSK	Q08881	ITK
Albiflorin	0.6311	Coagulation factor XI	FA11_HUMAN	F11
Albiflorin	0.6308	RAC-alpha serine/threonine-protein kinase	AKT1_HUMAN	AKT1
Albiflorin	0.6258	Glutathione S-transferase A1	P08263	GSTA1
Albiflorin	0.6241	Nuclear receptor subfamily 1 group I member 2	NR1I2_HUMAN	NR1I2
Albiflorin	0.6239	Fatty acid-binding protein, adipocyte	FABP4_HUMAN	FABP4
Albiflorin	0.6234	Serum amyloid P-component	P02743	APCS
Albiflorin	0.6226	Peroxisome proliferator-activated receptor alpha	PPARA_HUMAN	PPARA
Albiflorin	0.6179	Baculoviral IAP repeat-containing protein 7	Q96CA5	BIRC7
Albiflorin	0.6168	Cytidine deaminase	CDD_HUMAN	CDA
Albiflorin	0.6149	Interleukin-2	IL2_HUMAN	IL2
Albiflorin	0.6147	L-serine dehydratase	P20132	SDS
Albiflorin	0.6143	Trafficking protein particle complex subunit 3	O43617	TRAPPC3
Albiflorin	0.6142	Tyrosine-protein kinase JAK3	JAK3_HUMAN	JAK3
Albiflorin	0.6141	Alcohol dehydrogenase class-3	ADHX_HUMAN	ADH5
Albiflorin	0.6108	Flavin reductase	P30043	BLVRB
Albiflorin	0.6099	ADP-ribosyl cyclase 2	BST1_HUMAN	BST1
Albiflorin	0.6097	ADAM 17	ADA17_HUMAN	ADAM17
Albiflorin	0.6066	Fructose-bisphosphate aldolase A	ALDOA_HUMAN	ALDOA
Albiflorin	0.6055	S-adenosylmethionine decarboxylase proenzyme	P17707	AMD1
Albiflorin	0.6034	Receptor tyrosine-protein kinase erbB-4	ERBB4_HUMAN	ERBB4
Albiflorin	0.6019	Adenosine kinase	P55263	ADK
Albiflorin	0.5963	Ornithine carbamoyltransferase, mitochondrial	P00480	OTC
Albiflorin	0.5959	Lactoylglutathione lyase	LGUL_HUMAN	GLO1
Albiflorin	0.595	Endoplasmic reticulum mannosyl-oligosaccharide 1,2-alpha-mannosidase	Q9UKM7	MAN1B1
Albiflorin	0.5914	C-C motif chemokine 5	CCL5_HUMAN	CCL5

Albiflorin	0.5907	Leukotriene A-4 hydrolase	LKHA4_HUMAN	LTA4H
Albiflorin	0.5898	Cathepsin G	CATG_HUMAN	CTSG
Albiflorin	0.5885	B transferase	Q9NY01	ABO
Albiflorin	0.5881	Angiotensin-converting enzyme	ACE_HUMAN	ACE
Albiflorin	0.5857	NAD-dependent malic enzyme, mitochondrial	MAOM_HUMAN	ME2
Albiflorin	0.5856	T-cell surface glycoprotein CD1a	CD1A_HUMAN	CD1A
Albiflorin	0.5849	Cyclin-T1	CCNT1_HUMAN	CCNT1
Albiflorin	0.583	Glucokinase	HXK4_HUMAN	GCK
Albiflorin	0.5829	Serine hydroxymethyltransferase, cytosolic	GLYC_HUMAN	SHMT1
Albiflorin	0.5826	Interferon-stimulated gene 20 kDa protein	ISG20_HUMAN	ISG20
Albiflorin	0.5826	Copper transport protein ATOX1	O00244	ATOX1
Albiflorin	0.5823	Galactokinase	GALK1_HUMAN	GALK1
Albiflorin	0.5811	Uridine-cytidine kinase 2	Q9BZX2	UCK2
Albiflorin	0.5809	Inosine-5-monophosphate dehydrogenase 2	IMDH2_HUMAN	IMPDH2
Albiflorin	0.5765	Aldehyde dehydrogenase, mitochondrial	ALDH2_HUMAN	ALDH2
Albiflorin	0.5762	Tyrosine-protein kinase CSK	CSK_HUMAN	CSK
Albiflorin	0.5755	Beta-hexosaminidase subunit beta	P07686	HEXB
Albiflorin	0.5749	Rho GTPase-activating protein 1	Q07960	ARHGAP1
Albiflorin	0.5742	Neuronal calcium sensor 1	NCS1_HUMAN	NCS1
Albiflorin	0.5735	Tyrosine-protein kinase JAK2	JAK2_HUMAN	JAK2
Albiflorin	0.5723	Prostatic acid phosphatase	PPAP_HUMAN	ACP3
Albiflorin	0.5693	Transforming protein RhoA	P61586	RHOA
Albiflorin	0.5681	Beta-hexosaminidase beta chain	P07686	HEXB
Albiflorin	0.5678	Phosphoenolpyruvate carboxykinase, cytosolic [GTP]	P35558	PCK1
Albiflorin	0.5652	Galectin-3	LEG3_HUMAN	LGALS3
Albiflorin	0.5646	Matrilysin	MMP7_HUMAN	MMP7
Albiflorin	0.5645	Protein-glutamine gamma-glutamyltransferase E	TGM3_HUMAN	TGM3
Albiflorin	0.5645	L-lactate dehydrogenase B chain	P07195	LDHB
Albiflorin	0.5641	Ribonuclease 4	P34096	RNASE4
Albiflorin	0.5627	Thyroid hormone receptor beta-2	P37243	THRB
Albiflorin	0.5624	Fatty acid-binding protein, brain	FABP7_HUMAN	FABP7
Albiflorin	0.5603	Fatty acid-binding protein, heart	FABPH_HUMAN	FABP3
Albiflorin	0.5579	Cytochrome P450 2C8	CP2C8_HUMAN	CYP2C8
Albiflorin	0.5568	Gastrotropin	FABP6_HUMAN	FABP6
Albiflorin	0.5556	Nitric oxide synthase, endothelial	NOS3_HUMAN	NOS3
Albiflorin	0.555	Retinoic acid receptor gamma	RARG_HUMAN	RARG
Albiflorin	0.5542	Baculoviral IAP repeat-containing protein 4	P98170	XIAP
Albiflorin	0.5541	Ras-related protein Rab-5A	RAB5A_HUMAN	RAB5A
Albiflorin	0.5533	Thyroid hormone receptor alpha	P10827	THRA
Albiflorin	0.5532	72 kDa type IV collagenase	MMP2_HUMAN	MMP2
Albiflorin	0.5503	Tissue-type plasminogen activator	TPA_HUMAN	PLAT
Albiflorin	0.5502	Dipeptidase 1	DPEP1_HUMAN	DPEP1

Albiflorin	0.5484	Cathepsin F	Q9UBX1	CTSF
Albiflorin	0.5478	Disintegrin and metalloproteinase domain-containing protein 17	P78536	ADAM17
Albiflorin	0.5477	Sulfotransferase family cytosolic 2B member 1	O00204	SULT2B1
Albiflorin	0.5463	Insulin-like growth factor IA	P01343	IGF1
Albiflorin	0.5453	Fatty acid-binding protein, epidermal	FABP5_HUMAN	FABP5
Albiflorin	0.5448	Tyrosine-protein kinase ZAP-70	ZAP70_HUMAN	ZAP70
Albiflorin	0.5433	Thymidine phosphorylase	TYPH_HUMAN	TYMP
Albiflorin	0.5411	UDP-glucose 4-epimerase	GALE_HUMAN	GALE
Albiflorin	0.5408	Proto-oncogene tyrosine-protein kinase ABL1	P00519	ABL1
Albiflorin	0.5395	Glucose-6-phosphate isomerase	G6PI_HUMAN	GPI
Albiflorin	0.5395	Phenylethanolamine N-methyltransferase	PNMT_HUMAN	PNMT
Albiflorin	0.5364	Arginase-2, mitochondrial	ARGI2_HUMAN	ARG2
Albiflorin	0.5341	Matrix metalloproteinase-9	MMP9_HUMAN	MMP9
Albiflorin	0.5336	3 histone mRNA exonuclease 1	Q8IV48	ERI1
Albiflorin	0.5324	Protein kinase C theta type	KPCT_HUMAN	PRKCQ
Albiflorin	0.5317	Hepatocyte nuclear factor 4-gamma	Q14541	HNF4G
Albiflorin	0.528	RAC-beta serine/threonine-protein kinase	AKT2_HUMAN	AKT2
Albiflorin	0.5245	Protein S100-A9	S10A9_HUMAN	S100A9
Albiflorin	0.5229	Estrogen sulfotransferase	ST1E1_HUMAN	SULT1E1
Albiflorin	0.5216	cAMP-dependent protein kinase, alpha-catalytic subunit	P00517	PRKACA
Albiflorin	0.5203	Bis(5-adenosyl)-triphosphatase	P49789	FHIT
Albiflorin	0.5197	Pyruvate dehydrogenase E1 component subunit beta, mitochondrial	P11177	PDHB
Albiflorin	0.5193	Histone-lysine N-methyltransferase SETD7	SETD7_HUMAN	SETD7
Albiflorin	0.5151	Cellular retinoic acid-binding protein 2	P29373	CRABP2
Albiflorin	0.5148	Medium-chain specific acyl-CoA dehydrogenase, mitochondrial	ACADM_HUMAN	ACADM
Albiflorin	0.5135	Ras-related protein Rab-11A	RB11A_HUMAN	RAB11A
Albiflorin	0.513	Nuclear receptor subfamily 1 group I member 3	NR1I3_HUMAN	NR1I3
Albiflorin	0.5119	Dual specificity mitogen-activated protein kinase kinase 1	MP2K1_HUMAN	MAP2K1
Albiflorin	0.5107	Eosinophil cationic protein	P12724	RNASE3
Albiflorin	0.5095	Uridine 5-monophosphate synthase	P11172	UMPS
Albiflorin	0.5059	Transforming growth factor beta-2	TGFB2_HUMAN	TGFB2
Albiflorin	0.5029	Protein-arginine deiminase type-4	PADI4_HUMAN	PADI4
Paeonidaninol A	0.9905	Reticulon-4 receptor	RTN4R_HUMAN	RTN4R
Paeonidaninol A	0.9853	Mitogen-activated protein kinase 1	MK01_HUMAN	MAPK1
Paeonidaninol A	0.9843	Glutathione S-transferase P	GSTP1_HUMAN	GSTP1
Paeonidaninol A	0.9791	Cholinesterase	CHLE_HUMAN	BCHE
Paeonidaninol A	0.9754	Probable ATP-dependent RNA helicase DDX6	DDX6_HUMAN	DDX6
Paeonidaninol A	0.9744	cAMP-specific 3,5-cyclic phosphodiesterase 4B	PDE4B_HUMAN	PDE4B
Paeonidaninol A	0.9729	Cathepsin L2	CATL2_HUMAN	CTSV
Paeonidaninol A	0.9729	Ephrin type-B receptor 4	EPHB4_HUMAN	EPHB4
Paeonidaninol A	0.9725	Triggering receptor expressed on myeloid cells 1	Q9NP99	TREM1

Paeonidaninol A	0.9717	Methionine aminopeptidase 1	AMPM1_HUMAN	METAP1
Paeonidaninol A	0.97	Transthyretin	TTHY_HUMAN	TTR
Paeonidaninol A	0.9699	Cathepsin S	CATS_HUMAN	CTSS
Paeonidaninol A	0.9687	Peptidyl-prolyl cis-trans isomerase A	P62937	PPIA
Paeonidaninol A	0.966	Cathepsin D	CATD_HUMAN	CTSD
Paeonidaninol A	0.9613	Angiogenin	ANGI_HUMAN	ANG
Paeonidaninol A	0.9608	Oxysterols receptor LXR-beta	NR1H2_HUMAN	NR1H2
Paeonidaninol A	0.9592	Carbonic anhydrase 12	CAH12_HUMAN	CA12
Paeonidaninol A	0.9549	Carbonic anhydrase 2	CAH2_HUMAN	CA2
Paeonidaninol A	0.9541	Proto-oncogene serine/threonine-protein kinase Pim-1	PIM1_HUMAN	PIM1
Paeonidaninol A	0.9526	Peptidyl-prolyl cis-trans isomerase FKBP1A	FKB1A_HUMAN	FKBP1A
Paeonidaninol A	0.95	ADP-ribose pyrophosphatase, mitochondrial	Q9BW91	NUDT9
Paeonidaninol A	0.9468	Stromelysin-1	MMP3_HUMAN	MMP3
Paeonidaninol A	0.9465	Estrogen receptor	ESR1_HUMAN	ESR1
Paeonidaninol A	0.9449	TGF-beta receptor type-2	TGFR2_HUMAN	TGFBR2
Paeonidaninol A	0.9448	Mitogen-activated protein kinase 10	MK10_HUMAN	MAPK10
Paeonidaninol A	0.9426	Purine nucleoside phosphorylase	PNPH_HUMAN	PNP
Paeonidaninol A	0.9416	Progesterone receptor	PRGR_HUMAN	PGR
Paeonidaninol A	0.9401	Cyclin-dependent kinase 5 activator 1	CD5R1_HUMAN	CDK5R1
Paeonidaninol A	0.9384	Mitogen-activated protein kinase 8	MK08_HUMAN	MAPK8
Paeonidaninol A	0.932	Seprase	SEPR_HUMAN	FAP
Paeonidaninol A	0.9318	Peroxisome proliferator-activated receptor gamma	PPARG_HUMAN	PPARG
Paeonidaninol A	0.9306	Caspase-3	CASP3_HUMAN	CASP3
Paeonidaninol A	0.9175	Complement factor B	CFAB_HUMAN	CFB
Paeonidaninol A	0.9145	Epidermal growth factor receptor	EGFR_HUMAN	EGFR
Paeonidaninol A	0.9144	Cyclin-A2	CCNA2_HUMAN	CCNA2
Paeonidaninol A	0.9075	Serine/threonine-protein kinase Chk1	CHK1_HUMAN	CHEK1
Paeonidaninol A	0.8857	Carbonic anhydrase 1	CAH1_HUMAN	CA1
Paeonidaninol A	0.8826	TGF-beta receptor type-1	TGFR1_HUMAN	TGFBR1
Paeonidaninol A	0.8789	Prothrombin	THRB_HUMAN	F2
Paeonidaninol A	0.8484	Cell division protein kinase 2	P24941	CDK2
Paeonidaninol A	0.8285	Tyrosine-protein kinase HCK	HCK_HUMAN	HCK
Paeonidaninol A	0.827	Glycogen phosphorylase, liver form	P06737	PYGL
Paeonidaninol A	0.8241	Leukotriene A-4 hydrolase	LKHA4_HUMAN	LTA4H
Paeonidaninol A	0.8054	Death-associated protein kinase 1	DAPK1_HUMAN	DAPK1
Paeonidaninol A	0.7992	MAP kinase-activated protein kinase 2	P49137	MAPKAPK2
Paeonidaninol A	0.7978	S-methyl-5-thioadenosine phosphorylase	Q13126	MTAP
Paeonidaninol A	0.7976	Aldose reductase	ALDR_HUMAN	AKR1B1
Paeonidaninol A	0.7947	Liver carboxylesterase 1	EST1_HUMAN	CES1
Paeonidaninol A	0.7662	Chitotriosidase-1	Q13231	CHIT1
Paeonidaninol A	0.7544	Mitogen-activated protein kinase 14	Q16539	MAPK14
Paeonidaninol A	0.7502	Macrophage metalloelastase	MMP12_HUMAN	MMP12

Paeonidaninol A	0.75	Complement factor D	CFAD_HUMAN	CFD
Paeonidaninol A	0.7456	Serine/threonine-protein kinase PAK 7	PAK7_HUMAN	PAK7
Paeonidaninol A	0.7455	Bone morphogenetic protein 7	BMP7_HUMAN	BMP7
Paeonidaninol A	0.7451	Peroxisome proliferator-activated receptor delta	Q03181	PPARD
Paeonidaninol A	0.7448	Estrogen receptor beta	Q62986	ESR2
Paeonidaninol A	0.744	Kinesin-like protein KIF11	KIF11_HUMAN	KIF11
Paeonidaninol A	0.7439	Proto-oncogene tyrosine-protein kinase Src	SRC_HUMAN	SRC
Paeonidaninol A	0.742	Bile salt sulfotransferase	Q06520	SULT2A1
Paeonidaninol A	0.7414	Zinc-alpha-2-glycoprotein	ZA2G_HUMAN	AZGP1
Paeonidaninol A	0.7392	Chymase	CMA1_HUMAN	CMA1
Paeonidaninol A	0.7378	Macrophage migration inhibitory factor	MIF_HUMAN	MIF
Paeonidaninol A	0.7375	Amine oxidase [flavin-containing] B	AOFB_HUMAN	MAOB
Paeonidaninol A	0.7363	Serum amyloid P-component	P02743	APCS
Paeonidaninol A	0.7341	C-1-tetrahydrofolate synthase, cytoplasmic	P11586	MTHFD1
Paeonidaninol A	0.7322	Tyrosine-protein phosphatase non-receptor type 1	PTN1_HUMAN	PTPN1
Paeonidaninol A	0.7319	Protein farnesyltransferase/geranylgeranyltransferase type-1 subunit alpha	FNTA_HUMAN	FNTA
Paeonidaninol A	0.7312	Annexin A5	ANXA5_HUMAN	ANXA5
Paeonidaninol A	0.7305	Coagulation factor X	FA10_HUMAN	F10
Paeonidaninol A	0.7299	Phenylalanine-4-hydroxylase	PH4H_HUMAN	PAH
Paeonidaninol A	0.7294	Casein kinase II subunit alpha	P68400	CSNK2A1
Paeonidaninol A	0.7271	Pyridoxine-5-phosphate oxidase	Q9NVS9	PNPO
Paeonidaninol A	0.727	Caspase-7	CASP7_HUMAN	CASP7
Paeonidaninol A	0.7257	Heat shock protein HSP 90-alpha	HS90A_HUMAN	HSP90AA1
Paeonidaninol A	0.7245	Adenosylhomocysteinase	SAHH_HUMAN	AHCY
Paeonidaninol A	0.7219	Lanosterol synthase	ERG7_HUMAN	LSS
Paeonidaninol A	0.7218	Ig lambda chain V-II region MGC	P01709	IGLV2-8
Paeonidaninol A	0.7217	cGMP-specific 3,5-cyclic phosphodiesterase	PDE5A_HUMAN	PDE5A
Paeonidaninol A	0.7208	Inositol monophosphatase	IMPA1_HUMAN	IMPA1
Paeonidaninol A	0.7163	Androgen receptor	ANDR_HUMAN	AR
Paeonidaninol A	0.7152	Group 10 secretory phospholipase A2	O15496	PLA2G10
Paeonidaninol A	0.7151	cAMP-specific 3,5-cyclic phosphodiesterase 4D	PDE4D_HUMAN	PDE4D
Paeonidaninol A	0.7146	Galectin-7	LEG7_HUMAN	LGALS7
Paeonidaninol A	0.7138	Cathepsin B	CATB_HUMAN	CTSB
Paeonidaninol A	0.713	Tyrosine-protein kinase SYK	KSYK_HUMAN	SYK
Paeonidaninol A	0.712	Sex hormone-binding globulin	SHBG_HUMAN	SHBG
Paeonidaninol A	0.7061	NAD(P)H dehydrogenase [quinone] 1	NQO1_HUMAN	NQO1
Paeonidaninol A	0.7055	Urokinase-type plasminogen activator	UROK_HUMAN	PLAU
Paeonidaninol A	0.7046	Complement C1r subcomponent	P00736	C1R
Paeonidaninol A	0.7029	Insulin-like growth factor 1 receptor	P08069	IGF1R
Paeonidaninol A	0.6987	Heat shock cognate 71 kDa protein	P11142	HSPA8
Paeonidaninol A	0.6984	Collagenase 3	MMP13_HUMAN	MMP13
Paeonidaninol A	0.6958	Estrogen-related receptor gamma	P62508	ESRRG

Paeonidaninol A	0.6953	Thymidine phosphorylase	TYPH_HUMAN	TYMP
Paeonidaninol A	0.695	3-phosphoinositide-dependent protein kinase 1	PDPK1_HUMAN	PDPK1
Paeonidaninol A	0.694	Retinoic acid receptor RXR-alpha	RXRA_HUMAN	RXRA
Paeonidaninol A	0.6936	Superoxide dismutase [Mn], mitochondrial	P04179	SOD2
Paeonidaninol A	0.6915	Glycogen synthase kinase-3 beta	GSK3B_HUMAN	GSK3B
Paeonidaninol A	0.6913	Casein kinase I isoform gamma-2	KC1G2_HUMAN	CSNK1G2
Paeonidaninol A	0.6883	Renin	RENI_HUMAN	REN
Paeonidaninol A	0.6881	Sorbitol dehydrogenase	DHSO_HUMAN	SORD
Paeonidaninol A	0.6878	cAMP-dependent protein kinase catalytic subunit alpha	P00517	PRKACA
Paeonidaninol A	0.6814	Hexokinase-1	HXK1_HUMAN	HK1
Paeonidaninol A	0.6799	Interferon-stimulated gene 20 kDa protein	ISG20_HUMAN	ISG20
Paeonidaninol A	0.6791	Vascular endothelial growth factor receptor 2	VGFR2_HUMAN	KDR
Paeonidaninol A	0.6779	Serine/threonine-protein kinase PLK1	P53350	PLK1
Paeonidaninol A	0.6757	Dipeptidyl peptidase 4	DPP4_HUMAN	DPP4
Paeonidaninol A	0.6754	Neutrophil collagenase	MMP8_HUMAN	MMP8
Paeonidaninol A	0.6674	Dihydrofolate reductase	DYR_HUMAN	DHFR
Paeonidaninol A	0.6652	Branched-chain-amino-acid aminotransferase, mitochondrial	O15382	BCAT2
Paeonidaninol A	0.6643	cGMP-inhibited 3,5-cyclic phosphodiesterase B	PDE3B_HUMAN	PDE3B
Paeonidaninol A	0.6638	BAG family molecular chaperone regulator 1	BAG1_HUMAN	BAG1
Paeonidaninol A	0.6593	Beta-secretase 1	BACE1_HUMAN	BACE1
Paeonidaninol A	0.6585	Cytochrome P450 2C9	CP2C9_HUMAN	CYP2C9
Paeonidaninol A	0.6577	Thymidylate synthase	TYSY_HUMAN	TYMS
Paeonidaninol A	0.6553	S-adenosylmethionine decarboxylase proenzyme	DCAM_HUMAN	AMD1
Paeonidaninol A	0.6498	Carnitine O-acetyltransferase	CACP_HUMAN	CRAT
Paeonidaninol A	0.6484	Galectin-2	LEG2_HUMAN	LGALS2
Paeonidaninol A	0.6467	Serum albumin	ALBU_HUMAN	ALB
Paeonidaninol A	0.6459	Glutathione reductase, mitochondrial	P00390	GSR
Paeonidaninol A	0.6452	Eosinophil cationic protein	P12724	RNASE3
Paeonidaninol A	0.6389	Phosphoenolpyruvate carboxykinase, cytosolic [GTP]	P35558	PCK1
Paeonidaninol A	0.6363	Nuclear receptor ROR-alpha	RORA_HUMAN	RORA
Paeonidaninol A	0.636	Cytidine deaminase	CDD_HUMAN	CDA
Paeonidaninol A	0.6351	Alcohol dehydrogenase 1B	ADH1B_HUMAN	ADH1B
Paeonidaninol A	0.6326	Protein-arginine deiminase type-4	PADI4_HUMAN	PADI4
Paeonidaninol A	0.6283	Disintegrin and metalloproteinase domain-containing protein 17	P78536	ADAM17
Paeonidaninol A	0.6181	Histo-blood group ABO system transferase	P16442	ABO
Paeonidaninol A	0.6142	Proto-oncogene tyrosine-protein kinase LCK	LCK_HUMAN	LCK
Paeonidaninol A	0.6068	[Pyruvate dehydrogenase [lipoamide]] kinase isozyme 2, mitochondrial	PDK2_HUMAN	PDK2
Paeonidaninol A	0.6053	Galactosylgalactosylxylosylprotein 3-beta-glucuronosyltransferase 1	B3GA1_HUMAN	B3GAT1
Paeonidaninol A	0.6039	Cathepsin K	CATK_HUMAN	CTSK
Paeonidaninol A	0.6024	GTP-binding protein Rheb	Q15382	RHEB
Paeonidaninol A	0.6018	ADP-ribosyl cyclase 2	BST1_HUMAN	BST1
Paeonidaninol A	0.5988	Epoxide hydrolase 2	HYES_HUMAN	EPHX2

Paeonidaninol A	0.598	Tyrosine-protein kinase JAK3	JAK3_HUMAN	JAK3
Paeonidaninol A	0.598	RAC-alpha serine/threonine-protein kinase	AKT1_HUMAN	AKT1
Paeonidaninol A	0.597	Glucosamine-6-phosphate isomerase	P46926	GNPDA1
Paeonidaninol A	0.5961	Phosphatidylinositol-4,5-bisphosphate 3-kinase catalytic subunit gamma isoform	PK3CG_HUMAN	PIK3CG
Paeonidaninol A	0.5961	Calmodulin	CALM_HUMAN	CALM
Paeonidaninol A	0.5951	Endoplasmic reticulum mannosyl-oligosaccharide 1,2-alpha-mannosidase	Q9UKM7	MAN1B1
Paeonidaninol A	0.5947	Estradiol 17-beta-dehydrogenase 1	P14061	HSD17B1
Paeonidaninol A	0.5939	Suppressor of tumorigenicity protein 14	ST14_HUMAN	ST14
Paeonidaninol A	0.5935	Basic fibroblast growth factor receptor 1	FGFR1_HUMAN	FGFR1
Paeonidaninol A	0.5926	Tyrosine-protein phosphatase non-receptor type 11	PTN11_HUMAN	PTPN11
Paeonidaninol A	0.5921	SEC14-like protein 2	O76054	SEC14L2
Paeonidaninol A	0.592	Inosine-5-monophosphate dehydrogenase 2	IMDH2_HUMAN	IMPDH2
Paeonidaninol A	0.5891	Matrilysin	MMP7_HUMAN	MMP7
Paeonidaninol A	0.5874	Ribonuclease 4	P34096	RNASE4
Paeonidaninol A	0.5872	Transforming protein RhoA	P61586	RHOA
Paeonidaninol A	0.5869	Corticosteroid 11-beta-dehydrogenase isozyme 1	P28845	HSD11B1
Paeonidaninol A	0.5863	Ras-related C3 botulinum toxin substrate 2	P15153	RAC2
Paeonidaninol A	0.5859	Pancreatic alpha-amylase	AMYP_HUMAN	AMY2A
Paeonidaninol A	0.5851	Cathepsin G	CATG_HUMAN	CTSG
Paeonidaninol A	0.585	Tyrosine-protein kinase ITK/TSK	Q08881	ITK
Paeonidaninol A	0.5849	Fibroblast growth factor receptor 2	P21802	FGFR2
Paeonidaninol A	0.5843	Complement component C8 gamma chain	P07360	C8G
Paeonidaninol A	0.5841	Carbonyl reductase [NADPH] 1	CBR1_HUMAN	CBR1
Paeonidaninol A	0.5833	Insulin-like growth factor IA	P01343	IGF1
Paeonidaninol A	0.5827	Tyrosyl-tRNA synthetase, cytoplasmic	P54577	YARS1
Paeonidaninol A	0.582	Deoxycytidine kinase	DCK_HUMAN	DCK
Paeonidaninol A	0.5796	Adenosine kinase	ADK_HUMAN	ADK
Paeonidaninol A	0.5792	Tissue-type plasminogen activator	TPA_HUMAN	PLAT
Paeonidaninol A	0.5787	Hepatocyte growth factor receptor	MET_HUMAN	MET
Paeonidaninol A	0.5779	Ornithine carbamoyltransferase, mitochondrial	P00480	OTC
Paeonidaninol A	0.5772	Neuronal calcium sensor 1	NCS1_HUMAN	NCS1
Paeonidaninol A	0.5768	Wiskott-Aldrich syndrome protein	WASP_HUMAN	WAS
Paeonidaninol A	0.5762	Copper transport protein ATOX1	O00244	ATOX1
Paeonidaninol A	0.5747	Mineralocorticoid receptor	MCR_HUMAN	NR3C2
Paeonidaninol A	0.5744	Cell division protein kinase 6	CDK6_HUMAN	CDK6
Paeonidaninol A	0.5744	3-hydroxy-3-methylglutaryl-coenzyme A reductase	HMDH_HUMAN	HMGCR
Paeonidaninol A	0.5743	Aldo-keto reductase family 1 member C2	AK1C2_HUMAN	AKR1C2
Paeonidaninol A	0.5742	Bis(5-adenosyl)-triphosphatase	P49789	FHIT
Paeonidaninol A	0.5734	Poly [ADP-ribose] polymerase 1	P09874	PARP1
Paeonidaninol A	0.5731	Alcohol dehydrogenase 1C	P00326	ADH1C
Paeonidaninol A	0.5731	Histone deacetylase 8	Q9BY41	HDAC8
Paeonidaninol A	0.5721	Troponin C, slow skeletal and cardiac muscles	P63316	TNNC1

Paeonidaninol A	0.5678	NAD-dependent malic enzyme, mitochondrial	MAOM_HUMAN	ME2
Paeonidaninol A	0.5676	Prostatic acid phosphatase	PPAP_HUMAN	ACP3
Paeonidaninol A	0.5652	Lithostathine-1-alpha	P05451	REG1A
Paeonidaninol A	0.5647	Phospholipase A2, membrane associated	P14555	PLA2G2A
Paeonidaninol A	0.5615	Serine protease hepsin	P05981	HPN
Paeonidaninol A	0.5606	Leukocyte elastase	ELNE_HUMAN	ELANE
Paeonidaninol A	0.5601	Aldehyde dehydrogenase, mitochondrial	ALDH2_HUMAN	ALDH2
Paeonidaninol A	0.554	L-lactate dehydrogenase B chain	P07195	LDHB
Paeonidaninol A	0.554	Activated CDC42 kinase 1	ACK1_HUMAN	TNK2
Paeonidaninol A	0.5505	Beta-hexosaminidase subunit beta	P07686	HEXB
Paeonidaninol A	0.5479	Aldo-keto reductase family 1 member C3	AK1C3_HUMAN	AKR1C3
Paeonidaninol A	0.546	Angiotensin-converting enzyme 2	ACE2_HUMAN	ACE2
Paeonidaninol A	0.5445	Lactoylglutathione lyase	LGUL_HUMAN	GLO1
Paeonidaninol A	0.5445	Phenylethanolamine N-methyltransferase	PNMT_HUMAN	PNMT
Paeonidaninol A	0.5402	C-C motif chemokine 5	CCL5_HUMAN	CCL5
Paeonidaninol A	0.5381	Nuclear receptor subfamily 1 group I member 2	NR1I2_HUMAN	NR1I2
Paeonidaninol A	0.5379	Uridine-cytidine kinase 2	Q9BZX2	UCK2
Paeonidaninol A	0.5374	Ras-related protein Rab-11A	RB11A_HUMAN	RAB11A
Paeonidaninol A	0.5368	Dihydroorotate dehydrogenase, mitochondrial	PYRD_HUMAN	DHODH
Paeonidaninol A	0.5361	E3 ubiquitin-protein ligase Mdm2	MDM2_HUMAN	MDM2
Paeonidaninol A	0.5359	Ribosyldihydronicotinamide dehydrogenase [quinone]	NQO2_HUMAN	NQO2
Paeonidaninol A	0.5345	Beta-hexosaminidase beta chain	P07686	HEXB
Paeonidaninol A	0.5338	Flavin reductase	P30043	BLVRB
Paeonidaninol A	0.5334	Alcohol dehydrogenase class-3	ADHX_HUMAN	ADH5
Paeonidaninol A	0.5305	Glutathione S-transferase theta-2	GSTT2_HUMAN	GSTT2BGSTT2
Paeonidaninol A	0.5303	Methionine aminopeptidase 2	AMPM2_HUMAN	METAP2
Paeonidaninol A	0.5301	Nitric oxide synthase, endothelial	NOS3_HUMAN	NOS3
Paeonidaninol A	0.528	Catenin alpha-1	CTNA1_HUMAN	CTNNA1
Paeonidaninol A	0.5259	Angiotensin-converting enzyme	ACE_HUMAN	ACE
Paeonin C	0.5258	Bile acid receptor	NR1H4_HUMAN	NR1H4
Paeonidaninol A	0.5252	Dual specificity protein kinase CLK1	CLK1_HUMAN	CLK1
Paeonidaninol A	0.5236	Bactericidal permeability-increasing protein	P17213	BPI
Paeonidaninol A	0.5212	Interleukin-2	IL2_HUMAN	IL2
Paeonidaninol A	0.5182	Fatty acid-binding protein, adipocyte	FABP4_HUMAN	FABP4
Paeonidaninol A	0.5175	Thymidylate kinase	KTHY_HUMAN	DTYMK
Paeonidaninol A	0.5165	Protein-glutamine gamma-glutamyltransferase E	TGM3_HUMAN	TGM3
Paeonidaninol A	0.5125	Glucose-6-phosphate isomerase	G6PI_HUMAN	GPI
Paeonidaninol A	0.5123	Receptor tyrosine-protein kinase erbB-4	ERBB4_HUMAN	ERBB4
Paeonidaninol A	0.5104	Uridine 5-monophosphate synthase	P11172	UMPS
Paeonidaninol A	0.5093	Inosine-5-monophosphate dehydrogenase 1	IMDH1_HUMAN	IMPDH1
Paeonidaninol A	0.5092	Farnesyl pyrophosphate synthetase	FPPS_HUMAN	FDPS
Paeonidaninol A	0.5078	Arginase-2, mitochondrial	ARGI2_HUMAN	ARG2

Paeonidaninol A	0.5055	Cystathionine beta-synthase	P35520	CBS
Paeonidaninol A	0.5035	UDP-glucose 4-epimerase	GALE_HUMAN	GALE
Paeonidaninol A	0.5018	Fatty acid-binding protein, epidermal	FABP5_HUMAN	FABP5
Paeonin C	1	Bone morphogenetic protein 2	BMP2_HUMAN	BMP2
Paeonin C	0.9955	Transthyretin	TTHY_HUMAN	TTR
Paeonin C	0.9925	Carbonic anhydrase 2	P00918	CA2
Paeonin C	0.99	ADAM 17	ADA17_HUMAN	ADAM17
Paeonin C	0.99	Aldo-keto reductase family 1 member C2	AK1C2_HUMAN	AKR1C2
Paeonin C	0.9855	Proto-oncogene serine/threonine-protein kinase Pim-1	PIM1_HUMAN	PIM1
Paeonin C	0.9846	Estrogen receptor	ESR1_HUMAN	ESR1
Paeonin C	0.9751	Progesterone receptor	PRGR_HUMAN	PGR
Paeonin C	0.9736	Androgen receptor	ANDR_HUMAN	AR
Paeonin C	0.9725	cAMP-specific 3,5-cyclic phosphodiesterase 4B	PDE4B_HUMAN	PDE4B
Paeonin C	0.9688	cAMP-specific 3,5-cyclic phosphodiesterase 4D	PDE4D_HUMAN	PDE4D
Paeonin C	0.967	Carbonic anhydrase 1	CAH1_HUMAN	CA1
Paeonin C	0.966	Beta-secretase 1	BACE1_HUMAN	BACE1
Paeonin C	0.9605	Reticulon-4 receptor	RTN4R_HUMAN	RTN4R
Paeonin C	0.9573	Collagenase 3	MMP13_HUMAN	MMP13
Paeonin C	0.9533	Bile salt sulfotransferase	Q06520	SULT2A1
Paeonin C	0.9517	Complement factor D	CFAD_HUMAN	CFD
Paeonin C	0.9442	Peptidyl-prolyl cis-trans isomerase A	P62937	PPIA
Paeonin C	0.9404	Serine/threonine-protein kinase Chk1	CHK1_HUMAN	CHEK1
Paeonin C	0.9383	TGF-beta receptor type-2	TGFR2_HUMAN	TGFBR2
Paeonin C	0.9363	Cholinesterase	CHLE_HUMAN	BCHE
Paeonin C	0.9343	Heat shock protein HSP 90-alpha	HS90A_HUMAN	HSP90AA1
Paeonin C	0.9325	S-methyl-5-thioadenosine phosphorylase	Q13126	MTAP
Paeonin C	0.9322	Sex hormone-binding globulin	SHBG_HUMAN	SHBG
Paeonin C	0.9277	Cell division protein kinase 2	P24941	CDK2
Paeonin C	0.9221	Aldo-keto reductase family 1 member C3	AK1C3_HUMAN	AKR1C3
Paeonin C	0.9172	Estradiol 17-beta-dehydrogenase 1	P14061	HSD17B1
Paeonin C	0.9097	Catenin alpha-1	CTNA1_HUMAN	CTNNA1
Paeonin C	0.8987	Group 10 secretory phospholipase A2	O15496	PLA2G10
Paeonin C	0.8983	Corticosteroid 11-beta-dehydrogenase isozyme 1	DHI1_HUMAN	HSD11B1
Paeonin C	0.8981	Putative ATP-dependent Clp protease proteolytic subunit, mitochondrial	CLPP_HUMAN	CLPP
Paeonin C	0.8967	Mineralocorticoid receptor	MCR_HUMAN	NR3C2
Paeonin C	0.8909	Nuclear receptor ROR-alpha	RORA_HUMAN	RORA
Paeonin C	0.8803	Mitogen-activated protein kinase 14	Q16539	MAPK14
Paeonin C	0.8792	TGF-beta receptor type-1	TGFR1_HUMAN	TGFBR1
Paeonin C	0.8771	Wiskott-Aldrich syndrome protein	WASP_HUMAN	WAS
Paeonin C	0.8771	Estradiol 17-beta-dehydrogenase 11	DHB11_HUMAN	HSD17B11
Paeonin C	0.8695	Prothrombin	THRB_HUMAN	F2

Paeonin C	0.8643	Triggering receptor expressed on myeloid cells 1	Q9NP99	TREM1
Paeonin C	0.8603	Carbonic anhydrase 12	CAH12_HUMAN	CA12
Paeonin C	0.8474	Serum albumin	ALBU_HUMAN	ALB
Paeonin C	0.847	Basic fibroblast growth factor receptor 1	FGFR1_HUMAN	FGFR1
Paeonin C	0.8456	Troponin C, slow skeletal and cardiac muscles	P63316	TNNC1
Paeonin C	0.8416	Methionine aminopeptidase 1	AMPM1_HUMAN	METAP1
Paeonin C	0.8307	3-phosphoinositide-dependent protein kinase 1	PDPK1_HUMAN	PDPK1
Paeonin C	0.8289	Coagulation factor X	FA10_HUMAN	F10
Paeonin C	0.8266	Glycogen synthase kinase-3 beta	P49841	GSK3B
Paeonin C	0.8262	Phosphatidylinositol-4,5-bisphosphate 3-kinase catalytic subunit gamma isoform	PK3CG_HUMAN	PIK3CG
Paeonin C	0.8249	Ribosyldihydronicotinamide dehydrogenase [quinone]	P16083	NQO2
Paeonin C	0.8181	Cathepsin D	CATD_HUMAN	CTSD
Paeonin C	0.8126	Chymase	CMA1_HUMAN	CMA1
Paeonin C	0.8106	Annexin A5	ANXA5_HUMAN	ANXA5
Paeonin C	0.8071	NAD(P)H dehydrogenase [quinone] 1	NQO1_HUMAN	NQO1
Paeonin C	0.8027	SEC14-like protein 2	O76054	SEC14L2
Paeonin C	0.7985	Renin	RENI_HUMAN	REN
Paeonin C	0.7963	Oxysterols receptor LXR-alpha	NR1H3_HUMAN	NR1H3
Paeonin C	0.793	Methionine aminopeptidase 2	AMPM2_HUMAN	METAP2
Paeonin C	0.7918	Thymidylate synthase	TYSY_HUMAN	TYMS
Paeonin C	0.7906	Estrogen-related receptor gamma	P62508	ESRRG
Paeonin C	0.7892	Hepatocyte growth factor receptor	P08581	MET
Paeonin C	0.7863	Liver carboxylesterase 1	EST1_HUMAN	CES1
Paeonin C	0.7795	Stromelysin-1	MMP3_HUMAN	MMP3
Paeonin C	0.7794	Ephrin type-B receptor 4	EPHB4_HUMAN	EPHB4
Paeonin C	0.7783	Neutrophil collagenase	MMP8_HUMAN	MMP8
Paeonin C	0.7752	Tyrosyl-tRNA synthetase, cytoplasmic	P54577	YARS1
Paeonin C	0.7751	Retinoic acid receptor RXR-alpha	RXRA_HUMAN	RXRA
Paeonin C	0.7736	Aldose reductase	ALDR_HUMAN	AKR1B1
Paeonin C	0.7734	Serine/threonine-protein kinase 6	O14965	AURKA
Paeonin C	0.7728	Proto-oncogene tyrosine-protein kinase LCK	LCK_HUMAN	LCK
Paeonin C	0.7697	Superoxide dismutase [Mn], mitochondrial	P04179	SOD2
Paeonin C	0.7678	Vitamin D-binding protein	VTDB_HUMAN	GC
Paeonin C	0.7674	Epidermal growth factor receptor	EGFR_HUMAN	EGFR
Paeonin C	0.7623	Tyrosine-protein phosphatase non-receptor type 1	PTN1_HUMAN	PTPN1
Paeonin C	0.7602	Estrogen receptor beta	ESR2_HUMAN	ESR2
Paeonin C	0.7582	Histone deacetylase 8	HDAC8_HUMAN	HDAC8
Paeonin C	0.7567	cGMP-specific 3,5-cyclic phosphodiesterase	PDE5A_HUMAN	PDE5A
Paeonin C	0.7563	Cytochrome P450 2C9	CP2C9_HUMAN	CYP2C9
Paeonin C	0.7539	Glutaminyl-peptide cyclotransferase	QPCT_HUMAN	QPCT
Paeonin C	0.7533	Macrophage metalloelastase	MMP12_HUMAN	MMP12
Paeonin C	0.751	Epoxide hydrolase 2	HYES_HUMAN	EPHX2

Paeonin C	0.7491	Peroxisome proliferator-activated receptor alpha	PPARA_HUMAN	PPARA
Paeonin C	0.7489	Aldo-keto reductase family 1 member C1	Q04828	AKR1C1
Paeonin C	0.7473	Alcohol dehydrogenase 1C	P00326	ADH1C
Paeonin C	0.7472	Mitogen-activated protein kinase 1	MK01_HUMAN	MAPK1
Paeonin C	0.7431	Serum amyloid P-component	P02743	APCS
Paeonin C	0.743	cGMP-inhibited 3,5-cyclic phosphodiesterase B	PDE3B_HUMAN	PDE3B
Paeonin C	0.7429	Adenosine kinase	P55263	ADK
Paeonin C	0.7421	Kinesin-like protein KIF11	KIF11_HUMAN	KIF11
Paeonin C	0.7391	Macrophage migration inhibitory factor	MIF_HUMAN	MIF
Paeonin C	0.7333	Peroxisome proliferator-activated receptor gamma	PPARG_HUMAN	PPARG
Paeonin C	0.7292	Cathepsin B	CATB_HUMAN	CTSB
Paeonin C	0.7291	Caspase-3	CASP3_HUMAN	CASP3
Paeonin C	0.7272	Sorbitol dehydrogenase	DHSO_HUMAN	SORD
Paeonin C	0.7254	Glutathione reductase, mitochondrial	P00390	GSR
Paeonin C	0.7243	Amine oxidase [flavin-containing] B	AOFB_HUMAN	MAOB
Paeonin C	0.7234	Cyclin-A2	CCNA2_HUMAN	CCNA2
Paeonin C	0.7228	Peptidyl-prolyl cis-trans isomerase FKBP1A	FKB1A_HUMAN	FKBP1A
Paeonin C	0.718	Purine nucleoside phosphorylase	PNPH_HUMAN	PNP
Paeonin C	0.7139	Dihydroorotate dehydrogenase, mitochondrial	PYRD_HUMAN	DHODH
Paeonin C	0.7095	Oxysterols receptor LXR-beta	NR1H2_HUMAN	NR1H2
Paeonin C	0.7086	cAMP-dependent protein kinase catalytic subunit alpha	P00517	PRKACA
Paeonin C	0.7079	Cathepsin K	CATK_HUMAN	CTSK
Paeonin C	0.7077	Dihydrofolate reductase	DYR_HUMAN	DHFR
Paeonin C	0.7076	Protein farnesyltransferase/geranylgeranyltransferase type-1 subunit alpha	FNTA_HUMAN	FNTA
Paeonin C	0.7066	Glutathione S-transferase A1	P08263	GSTA1
Paeonin C	0.7053	Vascular endothelial growth factor receptor 2	VGFR2_HUMAN	KDR
Paeonin C	0.7041	Caspase-7	CASP7_HUMAN	CASP7
Paeonin C	0.7008	Bone morphogenetic protein 7	BMP7_HUMAN	BMP7
Paeonin C	0.693	Proto-oncogene tyrosine-protein kinase Src	SRC_HUMAN	SRC
Paeonin C	0.6927	Insulin-like growth factor 1 receptor	P08069	IGF1R
Paeonin C	0.6906	Angiogenin	ANGI_HUMAN	ANG
Paeonin C	0.69	Carbonyl reductase [NADPH] 1	CBR1_HUMAN	CBR1
Paeonin C	0.6873	Fatty acid-binding protein, heart	FABPH_HUMAN	FABP3
Paeonin C	0.6871	Leukocyte elastase	ELNE_HUMAN	ELANE
Paeonin C	0.6862	Galectin-7	LEG7_HUMAN	LGALS7
Paeonin C	0.6848	72 kDa type IV collagenase	MMP2_HUMAN	MMP2
Paeonin C	0.6814	Glutathione S-transferase P	GSTP1_HUMAN	GSTP1
Paeonin C	0.68	Alpha-tocopherol transfer protein	P49638	TTPA
Paeonin C	0.6795	Complement C1r subcomponent	P00736	C1R
Paeonin C	0.6794	Tyrosine-protein kinase JAK2	JAK2_HUMAN	JAK2
Paeonin C	0.6784	Complement factor B	CFAB_HUMAN	CFB
Paeonin C	0.678	Bile acid receptor	NR1H4_HUMAN	NR1H4

Paeonin C	0.6759	Tyrosine-protein kinase JAK3	JAK3_HUMAN	JAK3
Paeonin C	0.6722	Poly [ADP-ribose] polymerase 1	P09874	PARP1
Paeonin C	0.6667	Tyrosine-protein kinase ITK/TSK	Q08881	ITK
Paeonin C	0.6667	Alcohol dehydrogenase class-3	ADHX_HUMAN	ADH5
Paeonin C	0.6649	Tyrosine-protein kinase HCK	HCK_HUMAN	HCK
Paeonin C	0.6644	Phenylalanine-4-hydroxylase	PH4H_HUMAN	PAH
Paeonin C	0.6633	Thyroid hormone receptor beta	P10828	THRB
Paeonin C	0.6632	Glycogen phosphorylase, liver form	P06737	PYGL
Paeonin C	0.6624	Coagulation factor VII	FA7_HUMAN	F7
Paeonin C	0.662	Serine/threonine-protein kinase PAK 7	PAK7_HUMAN	PAK7
Paeonin C	0.66	Fatty acid-binding protein, adipocyte	FABP4_HUMAN	FABP4
Paeonin C	0.6566	Deoxycytidine kinase	DCK_HUMAN	DCK
Paeonin C	0.6551	S-adenosylmethionine decarboxylase proenzyme	P17707	AMD1
Paeonin C	0.655	Alpha-1-antitrypsin	A1AT_HUMAN	SERPINA1
Paeonin C	0.6548	Integrin alpha-L	ITAL_HUMAN	ITGAL
Paeonin C	0.6539	Histo-blood group ABO system transferase	P16442	ABO
Paeonin C	0.6493	Gastrotropin	FABP6_HUMAN	FABP6
Paeonin C	0.6473	Cathepsin F	Q9UBX1	CTSF
Paeonin C	0.6462	Retinol-binding protein 4	RET4_HUMAN	RBP4
Paeonin C	0.6455	Tyrosine-protein kinase ZAP-70	ZAP70_HUMAN	ZAP70
Paeonin C	0.6446	Branched-chain-amino-acid aminotransferase, mitochondrial	O15382	BCAT2
Paeonin C	0.6436	MAP kinase-activated protein kinase 2	P49137	MAPKAPK2
Paeonin C	0.6413	Protein-arginine deiminase type-4	PADI4_HUMAN	PADI4
Paeonin C	0.6407	Protein S100-A9	S10A9_HUMAN	S100A9
Paeonin C	0.6378	[Pyruvate dehydrogenase [lipoamide]] kinase isozyme 2, mitochondrial	PDK2_HUMAN	PDK2
Paeonin C	0.6353	Mitogen-activated protein kinase 8	MK08_HUMAN	MAPK8
Paeonin C	0.6346	Mitogen-activated protein kinase 10	MK10_HUMAN	MAPK10
Paeonin C	0.628	Nuclear receptor subfamily 1 group I member 2	NR1I2_HUMAN	NR1I2
Paeonin C	0.623	Protein-glutamine gamma-glutamyltransferase E	TGM3_HUMAN	TGM3
Paeonin C	0.6219	E3 ubiquitin-protein ligase Mdm2	MDM2_HUMAN	MDM2
Paeonin C	0.6186	BAG family molecular chaperone regulator 1	BAG1_HUMAN	BAG1
Paeonin C	0.6181	Inositol monophosphatase	IMPA1_HUMAN	IMPA1
Paeonin C	0.6147	Sulfotransferase family cytosolic 2B member 1	O00204	SULT2B1
Paeonin C	0.6116	Dual specificity protein phosphatase 6	DUS6_HUMAN	DUSP6
Paeonin C	0.6101	Fatty acid-binding protein, brain	FABP7_HUMAN	FABP7
Paeonin C	0.6083	Zinc-alpha-2-glycoprotein	ZA2G_HUMAN	AZGP1
Paeonin C	0.6057	Baculoviral IAP repeat-containing protein 4	P98170	XIAP
Paeonin C	0.6049	Phospholipase A2, membrane associated	P14555	PLA2G2A
Paeonin C	0.6035	Hexokinase-1	HXK1_HUMAN	HK1
Paeonin C	0.6027	Serine/threonine-protein phosphatase 5	P53041	PPP5C
Paeonin C	0.6016	Cytochrome P450 2C8	CP2C8_HUMAN	CYP2C8
Paeonin C	0.5998	Nitric oxide synthase, endothelial	NOS3_HUMAN	NOS3

Paeonin C	0.5976	Tyrosine-protein kinase CSK	CSK_HUMAN	CSK
Paeonin C	0.5936	Histamine N-methyltransferase	P50135	HNMT
Paeonin C	0.5914	Interferon-stimulated gene 20 kDa protein	ISG20_HUMAN	ISG20
Paeonin C	0.5904	Serine hydroxymethyltransferase, cytosolic	GLYC_HUMAN	SHMT1
Paeonin C	0.59	C-1-tetrahydrofolate synthase, cytoplasmic	P11586	MTHFD1
Paeonin C	0.5862	Adenosylhomocysteinase	SAHH_HUMAN	AHCY
Paeonin C	0.5851	3-hydroxy-3-methylglutaryl-coenzyme A reductase	HMDH_HUMAN	HMGCR
Paeonin C	0.5818	Betaine--homocysteine S-methyltransferase 1	Q93088	BHMT
Paeonin C	0.5808	Vitamin D3 receptor	VDR_HUMAN	VDR
Paeonin C	0.5804	Retinoic acid receptor alpha	RARA_HUMAN	RARA
Paeonin C	0.5796	Retinoic acid receptor gamma	RARG_HUMAN	RARG
Paeonin C	0.5772	Cathepsin S	CATS_HUMAN	CTSS
Paeonin C	0.575	Ferrochelatase, mitochondrial	P22830	FECH
Paeonin C	0.5748	Urokinase-type plasminogen activator	UROK_HUMAN	PLAU
Paeonin C	0.5746	Protein kinase C theta type	KPCT_HUMAN	PRKCQ
Paeonin C	0.5745	Interleukin-2	IL2_HUMAN	IL2
Paeonin C	0.5733	Cytidine deaminase	CDD_HUMAN	CDA
Paeonin C	0.5731	Pancreatic alpha-amylase	AMYP_HUMAN	AMY2A
Paeonin C	0.5729	Trafficking protein particle complex subunit 3	O43617	TRAPPC3
Paeonin C	0.5721	Neprilysin	NEP_HUMAN	MME
Paeonin C	0.5714	Thyroid hormone receptor alpha	P10827	THRA
Paeonin C	0.5701	Neuronal calcium sensor 1	NCS1_HUMAN	NCS1
Paeonin C	0.5675	Thymidine phosphorylase	TYPH_HUMAN	TYMP
Paeonin C	0.5665	Proto-oncogene tyrosine-protein kinase ABL1	P00519	ABL1
Paeonin C	0.5662	Cyclin-dependent kinase 5 activator 1	CD5R1_HUMAN	CDK5R1
Paeonin C	0.566	Tyrosine-protein kinase SYK	KSYK_HUMAN	SYK
Paeonin C	0.5658	Dipeptidase 1	DPEP1_HUMAN	DPEP1
Paeonin C	0.5635	Cell division protein kinase 6	CDK6_HUMAN	CDK6
Paeonin C	0.5615	Activated CDC42 kinase 1	ACK1_HUMAN	TNK2
Paeonin C	0.5611	Glucocorticoid receptor	GCR_HUMAN	NR3C1
Paeonin C	0.5607	Matrilysin	MMP7_HUMAN	MMP7
Paeonin C	0.5579	cAMP-dependent protein kinase, alpha-catalytic subunit	P00517	PRKACA
Paeonin C	0.5578	Insulin-like growth factor IA	P01343	IGF1
Paeonin C	0.5573	T-cell surface glycoprotein CD1a	CD1A_HUMAN	CD1A
Paeonin C	0.5558	Tyrosine-protein phosphatase non-receptor type 11	PTN11_HUMAN	PTPN11
Paeonin C	0.5551	Cyclin-T1	CCNT1_HUMAN	CCNT1
Paeonin C	0.5487	Cathepsin G	CATG_HUMAN	CTSG
Paeonin C	0.5462	Transforming growth factor beta-2	TGFB2_HUMAN	TGFB2
Paeonin C	0.5455	Copper transport protein ATOX1	O00244	ATOX1
Paeonin C	0.5448	Placenta growth factor	PLGF_HUMAN	PGF
Paeonin C	0.5444	Dipeptidyl peptidase 4	DPP4_HUMAN	DPP4
Paeonin C	0.544	Fatty acid-binding protein, epidermal	FABP5_HUMAN	FABP5

Paeonin C	0.5394	Phenylethanolamine N-methyltransferase	PNMT_HUMAN	PNMT
Paeonin C	0.5387	Heat shock cognate 71 kDa protein	P11142	HSPA8
Paeonin C	0.537	Transforming protein RhoA	P61586	RHOA
Paeonin C	0.536	Lanosterol synthase	ERG7_HUMAN	LSS
Paeonin C	0.5352	Phosphoenolpyruvate carboxykinase, cytosolic [GTP]	P35558	PCK1
Paeonin C	0.5346	Dual specificity mitogen-activated protein kinase kinase 1	MP2K1_HUMAN	MAP2K1
Paeonin C	0.5272	Beta-hexosaminidase beta chain	P07686	HEXB
Paeonin C	0.5259	Ras-related C3 botulinum toxin substrate 2	P15153	RAC2
Paeonin C	0.5218	Cellular retinoic acid-binding protein 2	P29373	CRABP2
Paeonin C	0.521	Casein kinase II subunit alpha	P68400	CSNK2A1
Paeonin C	0.5198	L-lactate dehydrogenase B chain	P07195	LDHB
Paeonin C	0.5197	Serine protease hepsin	P05981	HPN
Paeonin C	0.5194	Endoplasmic reticulum mannosyl-oligosaccharide 1,2-alpha-mannosidase	Q9UKM7	MAN1B1
Paeonin C	0.5192	Leukotriene A-4 hydrolase	LKHA4_HUMAN	LTA4H
Paeonin C	0.5174	Baculoviral IAP repeat-containing protein 7	Q96CA5	BIRC7
Paeonin C	0.5151	Serine/threonine-protein kinase PLK1	P53350	PLK1
Paeonin C	0.5099	Histone-lysine N-methyltransferase SETD7	SETD7_HUMAN	SETD7
Paeonin C	0.5095	Medium-chain specific acyl-CoA dehydrogenase, mitochondrial	ACADM_HUMAN	ACADM
Paeonin C	0.5064	ADP-ribosyl cyclase 2	BST1_HUMAN	BST1
Paeonin C	0.506	Glucose-6-phosphate isomerase	G6PI_HUMAN	GPI
Paeonin C	0.5045	Ig lambda chain V-II region MGC	P01709	IGLV2-8
Paeonin C	0.5038	Hydroxyacyl-coenzyme A dehydrogenase, mitochondrial	Q16836	HADH
Paeonin C	0.5035	Fibroblast growth factor receptor 2	P21802	FGFR2
Paeonin C	0.5	Alcohol dehydrogenase 1B	ADH1B_HUMAN	ADH1B
5-Hydroxyisovanillic acid	0.9613	Probable ATP-dependent RNA helicase DDX6	DDX6_HUMAN	DDX6
5-Hydroxyisovanillic acid	0.889	Carbonic anhydrase 2	CAH2_HUMAN	CA2
5-Hydroxyisovanillic acid	0.8741	Proto-oncogene serine/threonine-protein kinase Pim-1	PIM1_HUMAN	PIM1
5-Hydroxyisovanillic acid	0.8553	Cholinesterase	CHLE_HUMAN	BCHE
5-Hydroxyisovanillic acid	0.8253	Heat shock cognate 71 kDa protein	P11142	HSPA8
5-Hydroxyisovanillic acid	0.8013	Cell division protein kinase 2	P24941	CDK2
5-Hydroxyisovanillic acid	0.7458	Neutrophil gelatinase-associated lipocalin	NGAL_HUMAN	LCN2
5-Hydroxyisovanillic acid	0.7447	cAMP-specific 3,5-cyclic phosphodiesterase 4D	PDE4D_HUMAN	PDE4D
5-Hydroxyisovanillic acid	0.7058	Galectin-7	LEG7_HUMAN	LGALS7
5-Hydroxyisovanillic acid	0.7047	Angiogenin	ANGI_HUMAN	ANG

5-Hydroxyisovanillic acid	0.7008	Kinesin heavy chain	P33176	KIF5B
5-Hydroxyisovanillic acid	0.6756	Dihydrofolate reductase	DYR_HUMAN	DHFR
5-Hydroxyisovanillic acid	0.6676	Complement factor B	CFAB_HUMAN	CFB
5-Hydroxyisovanillic acid	0.6671	Serum amyloid P-component	P02743	APCS
5-Hydroxyisovanillic acid	0.6472	Nitric oxide synthase, endothelial	NOS3_HUMAN	NOS3
5-Hydroxyisovanillic acid	0.6362	Branched-chain-amino-acid aminotransferase, mitochondrial	O15382	BCAT2
5-Hydroxyisovanillic acid	0.5926	Serine/threonine-protein kinase Chk1	CHK1_HUMAN	CHEK1
5-Hydroxyisovanillic acid	0.5903	Ribonuclease 4	P34096	RNASE4
5-Hydroxyisovanillic acid	0.5895	NAD-dependent malic enzyme, mitochondrial	MAOM_HUMAN	ME2
5-Hydroxyisovanillic acid	0.5886	Pyridoxine-5-phosphate oxidase	Q9NVS9	PNPO
5-Hydroxyisovanillic acid	0.5865	Purine nucleoside phosphorylase	PNPH_HUMAN	PNP
5-Hydroxyisovanillic acid	0.5843	Thymidine phosphorylase	TYPH_HUMAN	TYMP
5-Hydroxyisovanillic acid	0.5793	Interferon-stimulated gene 20 kDa protein	ISG20_HUMAN	ISG20
5-Hydroxyisovanillic acid	0.5777	Pyruvate dehydrogenase E1 component subunit beta, mitochondrial	P11177	PDHB
5-Hydroxyisovanillic acid	0.5759	Heat shock protein HSP 90-alpha	HS90A_HUMAN	HSP90AA1
5-Hydroxyisovanillic acid	0.5754	Urokinase-type plasminogen activator	UROK_HUMAN	PLAU
5-Hydroxyisovanillic acid	0.5723	Cyclin-A2	CCNA2_HUMAN	CCNA2
5-Hydroxyisovanillic acid	0.5718	Carboxypeptidase B	P15086	CPB1
5-Hydroxyisovanillic acid	0.5692	Mitogen-activated protein kinase 8	P45983	MAPK8
5-Hydroxyisovanillic acid	0.5679	Transforming protein RhoA	P61586	RHOA
5-Hydroxyisovanillic acid	0.567	Hexokinase-1	HXK1_HUMAN	HK1
5-Hydroxyisovanillic acid	0.5666	Inosine-5-monophosphate dehydrogenase 2	IMDH2_HUMAN	IMPDH2
5-Hydroxyisovanillic acid	0.5629	Phenylalanine-4-hydroxylase	PH4H_HUMAN	PAH

5-Hydroxyisovanillic acid	0.5602	Adenosylhomocysteinase	SAHH_HUMAN	AHCY
5-Hydroxyisovanillic acid	0.5575	Mitogen-activated protein kinase 14	Q16539	MAPK14
5-Hydroxyisovanillic acid	0.5567	Prothrombin	THRB_HUMAN	F2
5-Hydroxyisovanillic acid	0.5559	Carbonyl reductase [NADPH] 1	CBR1_HUMAN	CBR1
5-Hydroxyisovanillic acid	0.5548	Activated CDC42 kinase 1	ACK1_HUMAN	TNK2
5-Hydroxyisovanillic acid	0.5532	Cytidine deaminase	CDD_HUMAN	CDA
5-Hydroxyisovanillic acid	0.5522	Superoxide dismutase [Mn], mitochondrial	P04179	SOD2
5-Hydroxyisovanillic acid	0.5517	Ornithine carbamoyltransferase, mitochondrial	P00480	OTC
5-Hydroxyisovanillic acid	0.5484	Galactosylgalactosylxylosylprotein 3-beta-glucuronosyltransferase 1	B3GA1_HUMAN	B3GAT1
5-Hydroxyisovanillic acid	0.5466	Complement component C8 gamma chain	P07360	C8G
5-Hydroxyisovanillic acid	0.5436	Glycogen phosphorylase, liver form	P06737	PYGL
5-Hydroxyisovanillic acid	0.5426	Tyrosine-protein phosphatase non-receptor type 1	PTN1_HUMAN	PTPN1
5-Hydroxyisovanillic acid	0.5403	Glycogen synthase kinase-3 beta	GSK3B_HUMAN	GSK3B
5-Hydroxyisovanillic acid	0.5367	Ephrin type-B receptor 4	EPHB4_HUMAN	EPHB4
5-Hydroxyisovanillic acid	0.5307	Aldose reductase	ALDR_HUMAN	AKR1B1
5-Hydroxyisovanillic acid	0.5256	Glutathione S-transferase theta-2	GSTT2_HUMAN	GSTT2BGSTT2
5-Hydroxyisovanillic acid	0.5204	Cell division protein kinase 6	CDK6_HUMAN	CDK6
5-Hydroxyisovanillic acid	0.515	Phosphoenolpyruvate carboxykinase, cytosolic [GTP]	P35558	PCK1
5-Hydroxyisovanillic acid	0.5091	Tyrosine-protein kinase JAK2	JAK2_HUMAN	JAK2
5-Hydroxyisovanillic acid	0.5085	Galactokinase	GALK1_HUMAN	GALK1
5-Hydroxyisovanillic acid	0.5057	Uridine-cytidine kinase 2	Q9BZX2	UCK2
Paeoniflorin	1	Kinesin-like protein KIF11	KIF11_HUMAN	KIF11
Paeoniflorin	1	Nuclear receptor ROR-alpha	RORA_HUMAN	RORA
Paeoniflorin	1	Bone morphogenetic protein 2	BMP2_HUMAN	BMP2
Paeoniflorin	1	Aldo-keto reductase family 1 member C2	AK1C2_HUMAN	AKR1C2

Paeoniflorin	1	Amine oxidase [flavin-containing] B	AOFB_HUMAN	MAOB
Paeoniflorin	0.9994	Annexin A5	ANXA5_HUMAN	ANXA5
Paeoniflorin	0.9993	Mitogen-activated protein kinase 1	MK01_HUMAN	MAPK1
Paeoniflorin	0.9992	Transthyretin	TTHY_HUMAN	TTR
Paeoniflorin	0.9991	Ephrin type-B receptor 4	EPHB4_HUMAN	EPHB4
Paeoniflorin	0.9986	Peptidyl-prolyl cis-trans isomerase A	P62937	PPIA
Paeoniflorin	0.9984	Carbonic anhydrase 2	CAH2_HUMAN	CA2
Paeoniflorin	0.9965	Proto-oncogene tyrosine-protein kinase Src	SRC_HUMAN	SRC
Paeoniflorin	0.9964	Glutathione S-transferase P	GSTP1_HUMAN	GSTP1
Paeoniflorin	0.9963	cAMP-specific 3,5-cyclic phosphodiesterase 4B	PDE4B_HUMAN	PDE4B
Paeoniflorin	0.9961	Beta-secretase 1	BACE1_HUMAN	BACE1
Paeoniflorin	0.9961	Stromelysin-1	MMP3_HUMAN	MMP3
Paeoniflorin	0.996	Epidermal growth factor receptor	EGFR_HUMAN	EGFR
Paeoniflorin	0.9959	Proto-oncogene serine/threonine-protein kinase Pim-1	PIM1_HUMAN	PIM1
Paeoniflorin	0.9953	Peroxisome proliferator-activated receptor delta	Q03181	PPARD
Paeoniflorin	0.9949	Purine nucleoside phosphorylase	PNPH_HUMAN	PNP
Paeoniflorin	0.9948	TGF-beta receptor type-1	TGFR1_HUMAN	TGFBR1
Paeoniflorin	0.9944	Aldose reductase	ALDR_HUMAN	AKR1B1
Paeoniflorin	0.9943	Mitogen-activated protein kinase 14	Q16539	MAPK14
Paeoniflorin	0.9935	Cathepsin L2	CATL2_HUMAN	CTSV
Paeoniflorin	0.993	Cathepsin S	CATS_HUMAN	CTSS
Paeoniflorin	0.9925	Estrogen receptor	ESR1_HUMAN	ESR1
Paeoniflorin	0.9924	Macrophage migration inhibitory factor	MIF_HUMAN	MIF
Paeoniflorin	0.9923	cAMP-specific 3,5-cyclic phosphodiesterase 4D	PDE4D_HUMAN	PDE4D
Paeoniflorin	0.9917	Estrogen-related receptor gamma	P62508	ESRRG
Paeoniflorin	0.991	Cyclin-A2	CCNA2_HUMAN	CCNA2
Paeoniflorin	0.9907	Progesterone receptor	PRGR_HUMAN	PGR
Paeoniflorin	0.9888	Mitogen-activated protein kinase 10	MK10_HUMAN	MAPK10
Paeoniflorin	0.9883	Peroxisome proliferator-activated receptor gamma	PPARG_HUMAN	PPARG
Paeoniflorin	0.9872	Bile salt sulfotransferase	Q06520	SULT2A1
Paeoniflorin	0.9869	Complement factor D	CFAD_HUMAN	CFD
Paeoniflorin	0.9861	ADP-ribose pyrophosphatase, mitochondrial	Q9BW91	NUDT9
Paeoniflorin	0.9858	Prothrombin	THRB_HUMAN	F2
Paeoniflorin	0.9848	Carbonic anhydrase 12	CAH12_HUMAN	CA12
Paeoniflorin	0.9835	Caspase-3	CASP3_HUMAN	CASP3
Paeoniflorin	0.9833	Caspase-7	CASP7_HUMAN	CASP7
Paeoniflorin	0.9827	Insulin-like growth factor 1 receptor	P08069	IGF1R
Paeoniflorin	0.9815	Complement factor B	CFAB_HUMAN	CFB
Paeoniflorin	0.9815	Mitogen-activated protein kinase 8	MK08_HUMAN	MAPK8
Paeoniflorin	0.9808	MAP kinase-activated protein kinase 2	P49137	MAPKAPK2
Paeoniflorin	0.9782	Androgen receptor	ANDR_HUMAN	AR
Paeoniflorin	0.9781	SEC14-like protein 2	O76054	SEC14L2

Paeoniflorin	0.9755	Cholinesterase	CHLE_HUMAN	BCHE
Paeoniflorin	0.9749	Liver carboxylesterase 1	EST1_HUMAN	CES1
Paeoniflorin	0.9746	Triggering receptor expressed on myeloid cells 1	Q9NP99	TREM1
Paeoniflorin	0.9732	Collagenase 3	MMP13_HUMAN	MMP13
Paeoniflorin	0.972	Cell division protein kinase 2	P24941	CDK2
Paeoniflorin	0.9717	Wiskott-Aldrich syndrome protein	WASP_HUMAN	WAS
Paeoniflorin	0.9712	Aldo-keto reductase family 1 member C3	AK1C3_HUMAN	AKR1C3
Paeoniflorin	0.9708	Heat shock protein HSP 90-alpha	HS90A_HUMAN	HSP90AA1
Paeoniflorin	0.9694	Tyrosine-protein kinase SYK	KSYK_HUMAN	SYK
Paeoniflorin	0.9688	Seprase	SEPR_HUMAN	FAP
Paeoniflorin	0.9685	Serine/threonine-protein kinase Chk1	CHK1_HUMAN	CHEK1
Paeoniflorin	0.9673	Probable ATP-dependent RNA helicase DDX6	DDX6_HUMAN	DDX6
Paeoniflorin	0.9671	Dihydrofolate reductase	DYR_HUMAN	DHFR
Paeoniflorin	0.9639	NAD(P)H dehydrogenase [quinone] 1	NQO1_HUMAN	NQO1
Paeoniflorin	0.9613	Corticosteroid 11-beta-dehydrogenase isozyme 1	P28845	HSD11B1
Paeoniflorin	0.96	ADAM 17	ADA17_HUMAN	ADAM17
Paeoniflorin	0.9588	TGF-beta receptor type-2	TGFR2_HUMAN	TGFBR2
Paeoniflorin	0.9586	Superoxide dismutase [Mn], mitochondrial	P04179	SOD2
Paeoniflorin	0.9558	Vascular endothelial growth factor receptor 2	VGFR2_HUMAN	KDR
Paeoniflorin	0.9549	Cathepsin D	CATD_HUMAN	CTSD
Paeoniflorin	0.9547	Serum albumin	ALBU_HUMAN	ALB
Paeoniflorin	0.9547	Sex hormone-binding globulin	SHBG_HUMAN	SHBG
Paeoniflorin	0.9502	Estradiol 17-beta-dehydrogenase 1	P14061	HSD17B1
Paeoniflorin	0.95	Methionine aminopeptidase 1	AMPM1_HUMAN	METAP1
Paeoniflorin	0.9469	Carbonic anhydrase 1	CAH1_HUMAN	CA1
Paeoniflorin	0.9449	Peptidyl-prolyl cis-trans isomerase FKBP1A	FKB1A_HUMAN	FKBP1A
Paeoniflorin	0.9447	RAC-alpha serine/threonine-protein kinase	AKT1_HUMAN	AKT1
Paeoniflorin	0.9399	S-methyl-5-thioadenosine phosphorylase	Q13126	MTAP
Paeoniflorin	0.9354	Lanosterol synthase	ERG7_HUMAN	LSS
Paeoniflorin	0.9342	Coagulation factor VII	FA7_HUMAN	F7
Paeoniflorin	0.934	Retinoic acid receptor RXR-alpha	RXRA_HUMAN	RXRA
Paeoniflorin	0.9339	Oxysterols receptor LXR-beta	NR1H2_HUMAN	NR1H2
Paeoniflorin	0.928	Phosphatidylinositol-4,5-bisphosphate 3-kinase catalytic subunit gamma isoform	PK3CG_HUMAN	PIK3CG
Paeoniflorin	0.9279	Troponin C, slow skeletal and cardiac muscles	P63316	TNNC1
Paeoniflorin	0.9257	Mineralocorticoid receptor	MCR_HUMAN	NR3C2
Paeoniflorin	0.925	Sorbitol dehydrogenase	DHSO_HUMAN	SORD
Paeoniflorin	0.9248	3-phosphoinositide-dependent protein kinase 1	PDPK1_HUMAN	PDPK1
Paeoniflorin	0.9151	Estradiol 17-beta-dehydrogenase 11	DHB11_HUMAN	HSD17B11
Paeoniflorin	0.9118	Inositol monophosphatase	IMPA1_HUMAN	IMPA1
Paeoniflorin	0.9116	Reticulon-4 receptor	RTN4R_HUMAN	RTN4R
Paeoniflorin	0.9106	Angiogenin	ANGI_HUMAN	ANG
Paeoniflorin	0.9084	Coagulation factor X	P00742	F10

Paeoniflorin	0.9042	Heat shock cognate 71 kDa protein	P11142	HSPA8
Paeoniflorin	0.8978	Glycogen synthase kinase-3 beta	P49841	GSK3B
Paeoniflorin	0.8958	Proto-oncogene tyrosine-protein kinase LCK	LCK_HUMAN	LCK
Paeoniflorin	0.8947	Tyrosine-protein kinase HCK	HCK_HUMAN	HCK
Paeoniflorin	0.8901	Cyclin-dependent kinase 5 activator 1	CD5R1_HUMAN	CDK5R1
Paeoniflorin	0.8848	Catenin alpha-1	CTNA1_HUMAN	CTNNA1
Paeoniflorin	0.8797	E3 ubiquitin-protein ligase Mdm2	MDM2_HUMAN	MDM2
Paeoniflorin	0.8771	Epoxide hydrolase 2	HYES_HUMAN	EPHX2
Paeoniflorin	0.8767	Cytochrome P450 2C9	CP2C9_HUMAN	CYP2C9
Paeoniflorin	0.8753	Renin	RENI_HUMAN	REN
Paeoniflorin	0.8745	Bile acid receptor	NR1H4_HUMAN	NR1H4
Paeoniflorin	0.8709	Aldo-keto reductase family 1 member C1	Q04828	AKR1C1
Paeoniflorin	0.8389	Ribosyldihydronicotinamide dehydrogenase [quinone]	P16083	NQO2
Paeoniflorin	0.8242	Trafficking protein particle complex subunit 3	O43617	TRAPPC3
Paeoniflorin	0.8238	Nuclear receptor subfamily 1 group I member 3	NR1I3_HUMAN	NR1I3
Paeoniflorin	0.8232	Basic fibroblast growth factor receptor 1	FGFR1_HUMAN	FGFR1
Paeoniflorin	0.8195	Leukocyte elastase	ELNE_HUMAN	ELANE
Paeoniflorin	0.8141	Methionine aminopeptidase 2	AMPM2_HUMAN	METAP2
Paeoniflorin	0.8073	Glutathione S-transferase A1	P08263	GSTA1
Paeoniflorin	0.8057	cGMP-specific 3,5-cyclic phosphodiesterase	PDE5A_HUMAN	PDE5A
Paeoniflorin	0.8028	Peroxisome proliferator-activated receptor alpha	PPARA_HUMAN	PPARA
Paeoniflorin	0.8016	Glutathione reductase, mitochondrial	P00390	GSR
Paeoniflorin	0.8014	Insulin-like growth factor IA	P01343	IGF1
Paeoniflorin	0.8007	Oxysterols receptor LXR-alpha	NR1H3_HUMAN	NR1H3
Paeoniflorin	0.8	Tyrosine-protein phosphatase non-receptor type 11	PTN11_HUMAN	PTPN11
Paeoniflorin	0.8	Alpha-tocopherol transfer protein	P49638	TTPA
Paeoniflorin	0.8	Histone deacetylase 8	Q9BY41	HDAC8
Paeoniflorin	0.8	Neutrophil collagenase	MMP8_HUMAN	MMP8
Paeoniflorin	0.7998	Alcohol dehydrogenase 1C	P00326	ADH1C
Paeoniflorin	0.7993	3-hydroxy-3-methylglutaryl-coenzyme A reductase	HMDH_HUMAN	HMGCR
Paeoniflorin	0.7987	Tyrosine-protein kinase ITK/TSK	Q08881	ITK
Paeoniflorin	0.7985	Phospholipase A2, membrane associated	P14555	PLA2G2A
Paeoniflorin	0.7982	T-cell surface glycoprotein CD1a	CD1A_HUMAN	CD1A
Paeoniflorin	0.7982	Adenosine kinase	P55263	ADK
Paeoniflorin	0.7974	Atrial natriuretic peptide clearance receptor	ANPRC_HUMAN	NPR3
Paeoniflorin	0.7965	Poly [ADP-ribose] polymerase 1	P09874	PARP1
Paeoniflorin	0.795	Serine/threonine-protein kinase PLK1	P53350	PLK1
Paeoniflorin	0.7931	cAMP-dependent protein kinase catalytic subunit alpha	P00517	PRKACA
Paeoniflorin	0.7919	Galectin-7	LEG7_HUMAN	LGALS7
Paeoniflorin	0.7916	Serine/threonine-protein kinase PAK 7	PAK7_HUMAN	PAK7
Paeoniflorin	0.7896	Tyrosine-protein kinase JAK3	JAK3_HUMAN	JAK3
Paeoniflorin	0.787	cGMP-inhibited 3,5-cyclic phosphodiesterase B	PDE3B_HUMAN	PDE3B

Paeoniflorin	0.7863	Aldehyde dehydrogenase, mitochondrial	ALDH2_HUMAN	ALDH2
Paeoniflorin	0.7854	Hexokinase-1	HXK1_HUMAN	HK1
Paeoniflorin	0.7836	Estrogen receptor beta	ESR2_HUMAN	ESR2
Paeoniflorin	0.7835	Hepatocyte growth factor receptor	P08581	MET
Paeoniflorin	0.7825	Cell division protein kinase 6	CDK6_HUMAN	CDK6
Paeoniflorin	0.7824	Tyrosine-protein phosphatase non-receptor type 1	PTN1_HUMAN	PTPN1
Paeoniflorin	0.7802	Chymase	CMA1_HUMAN	CMA1
Paeoniflorin	0.78	Cyclin-T1	CCNT1_HUMAN	CCNT1
Paeoniflorin	0.7781	Placenta growth factor	PLGF_HUMAN	PGF
Paeoniflorin	0.7766	Receptor tyrosine-protein kinase erbB-4	ERBB4_HUMAN	ERBB4
Paeoniflorin	0.7758	Deoxycytidine kinase	DCK_HUMAN	DCK
Paeoniflorin	0.7742	Dual specificity protein phosphatase 6	DUS6_HUMAN	DUSP6
Paeoniflorin	0.7731	Thyroid hormone receptor beta-2	P37243	THRB
Paeoniflorin	0.7726	Phenylalanine-4-hydroxylase	PH4H_HUMAN	PAH
Paeoniflorin	0.7722	Carbonyl reductase [NADPH] 1	CBR1_HUMAN	CBR1
Paeoniflorin	0.7705	Tyrosyl-tRNA synthetase, cytoplasmic	P54577	YARS1
Paeoniflorin	0.7694	Macrophage metalloelastase	MMP12_HUMAN	MMP12
Paeoniflorin	0.7673	Dipeptidyl peptidase 4	DPP4_HUMAN	DPP4
Paeoniflorin	0.7659	Histo-blood group ABO system transferase	P16442	ABO
Paeoniflorin	0.7616	Cathepsin K	CATK_HUMAN	CTSK
Paeoniflorin	0.7603	Flavin reductase	P30043	BLVRB
Paeoniflorin	0.75	Serine/threonine-protein phosphatase 5	P53041	PPP5C
Paeoniflorin	0.7451	[Pyruvate dehydrogenase [lipoamide]] kinase isozyme 2, mitochondrial	PDK2_HUMAN	PDK2
Paeoniflorin	0.7444	Casein kinase II subunit alpha	P68400	CSNK2A1
Paeoniflorin	0.7432	Alcohol dehydrogenase 1B	ADH1B_HUMAN	ADH1B
Paeoniflorin	0.7411	Calmodulin	CALM_HUMAN	CALM
Paeoniflorin	0.7395	Tyrosine-protein kinase CSK	CSK_HUMAN	CSK
Paeoniflorin	0.7382	Bone morphogenetic protein 7	BMP7_HUMAN	BMP7
Paeoniflorin	0.7381	Pyridoxine-5-phosphate oxidase	Q9NVS9	PNPO
Paeoniflorin	0.738	Group 10 secretory phospholipase A2	O15496	PLA2G10
Paeoniflorin	0.7365	Tyrosine-protein kinase ZAP-70	ZAP70_HUMAN	ZAP70
Paeoniflorin	0.7357	C-1-tetrahydrofolate synthase, cytoplasmic	P11586	MTHFD1
Paeoniflorin	0.7354	Dihydroorotate dehydrogenase, mitochondrial	PYRD_HUMAN	DHODH
Paeoniflorin	0.7336	Disintegrin and metalloproteinase domain-containing protein 17	P78536	ADAM17
Paeoniflorin	0.7333	Cathepsin B	CATB_HUMAN	CTSB
Paeoniflorin	0.7332	Gastrotropin	FABP6_HUMAN	FABP6
Paeoniflorin	0.7331	Death-associated protein kinase 1	DAPK1_HUMAN	DAPK1
Paeoniflorin	0.7282	Casein kinase I isoform gamma-2	KC1G2_HUMAN	CSNK1G2
Paeoniflorin	0.728	Ig lambda chain V-II region MGC	P01709	IGLV2-8
Paeoniflorin	0.728	Branched-chain-amino-acid aminotransferase, mitochondrial	O15382	BCAT2
Paeoniflorin	0.7265	Chitotriosidase-1	Q13231	CHIT1
Paeoniflorin	0.7259	Nitric oxide synthase, endothelial	NOS3_HUMAN	NOS3

Paeoniflorin	0.7228	S-adenosylmethionine decarboxylase proenzyme	DCAM_HUMAN	AMD1
Paeoniflorin	0.7223	Vitamin D-binding protein	VTDB_HUMAN	GC
Paeoniflorin	0.7215	Activated CDC42 kinase 1	ACK1_HUMAN	TNK2
Paeoniflorin	0.7212	Glycogen phosphorylase, liver form	P06737	PYGL
Paeoniflorin	0.721	BAG family molecular chaperone regulator 1	BAG1_HUMAN	BAG1
Paeoniflorin	0.7189	Fibroblast growth factor receptor 2	P21802	FGFR2
Paeoniflorin	0.7121	Cytidine deaminase	CDD_HUMAN	CDA
Paeoniflorin	0.7104	Hepatocyte nuclear factor 4-gamma	Q14541	HNF4G
Paeoniflorin	0.7098	Galactokinase	GALK1_HUMAN	GALK1
Paeoniflorin	0.7077	Alcohol dehydrogenase class-3	ADHX_HUMAN	ADH5
Paeoniflorin	0.7072	Dipeptidase 1	DPEP1_HUMAN	DPEP1
Paeoniflorin	0.7058	Protein farnesyltransferase/geranylgeranyltransferase type-1 subunit alpha	FNTA_HUMAN	FNTA
Paeoniflorin	0.7057	Serum amyloid P-component	P02743	APCS
Paeoniflorin	0.7043	GTP-binding protein Rheb	Q15382	RHEB
Paeoniflorin	0.6966	Carnitine O-acetyltransferase	CACP_HUMAN	CRAT
Paeoniflorin	0.6966	Thymidine phosphorylase	TYPH_HUMAN	TYMP
Paeoniflorin	0.6965	Cathepsin F	Q9UBX1	CTSF
Paeoniflorin	0.6963	Interferon-stimulated gene 20 kDa protein	ISG20_HUMAN	ISG20
Paeoniflorin	0.6961	Neutrophil gelatinase-associated lipocalin	NGAL_HUMAN	LCN2
Paeoniflorin	0.6959	Fatty acid-binding protein, brain	FABP7_HUMAN	FABP7
Paeoniflorin	0.6953	Cytochrome P450 2C8	CP2C8_HUMAN	CYP2C8
Paeoniflorin	0.6945	Tyrosine-protein kinase JAK2	JAK2_HUMAN	JAK2
Paeoniflorin	0.6945	Urokinase-type plasminogen activator	UROK_HUMAN	PLAU
Paeoniflorin	0.6913	Fatty acid-binding protein, heart	FABPH_HUMAN	FABP3
Paeoniflorin	0.6907	Pancreatic alpha-amylase	AMYP_HUMAN	AMY2A
Paeoniflorin	0.69	Thyroid hormone receptor beta	P10828	THRB
Paeoniflorin	0.6891	Thymidylate synthase	TYSY_HUMAN	TYMS
Paeoniflorin	0.689	Leukotriene A-4 hydrolase	LKHA4_HUMAN	LTA4H
Paeoniflorin	0.6882	Retinoic acid receptor gamma	RARG_HUMAN	RARG
Paeoniflorin	0.6881	Integrin alpha-L	ITAL_HUMAN	ITGAL
Paeoniflorin	0.6868	72 kDa type IV collagenase	MMP2_HUMAN	MMP2
Paeoniflorin	0.6803	Adenosylhomocysteinase	SAHH_HUMAN	AHCY
Paeoniflorin	0.6797	Fatty acid-binding protein, epidermal	FABP5_HUMAN	FABP5
Paeoniflorin	0.6781	Nuclear receptor subfamily 1 group I member 2	NR1I2_HUMAN	NR1I2
Paeoniflorin	0.6781	Thyroid hormone receptor alpha	P10827	THRA
Paeoniflorin	0.6767	Estrogen sulfotransferase	ST1E1_HUMAN	SULT1E1
Paeoniflorin	0.6764	Protein kinase C theta type	KPCT_HUMAN	PRKCQ
Paeoniflorin	0.6757	Sulfotransferase family cytosolic 2B member 1	O00204	SULT2B1
Paeoniflorin	0.6727	Zinc-alpha-2-glycoprotein	ZA2G_HUMAN	AZGP1
Paeoniflorin	0.6692	Protein S100-A9	S10A9_HUMAN	S100A9
Paeoniflorin	0.6685	Protein-glutamine gamma-glutamyltransferase E	TGM3_HUMAN	TGM3
Paeoniflorin	0.6667	Putative ATP-dependent Clp protease proteolytic subunit, mitochondrial	CLPP_HUMAN	CLPP

Paeoniflorin	0.6644	Interleukin-2	IL2_HUMAN	IL2
Paeoniflorin	0.6628	cAMP-dependent protein kinase, alpha-catalytic subunit	P00517	PRKACA
Paeoniflorin	0.6618	B transferase	Q9NY01	ABO
Paeoniflorin	0.6618	Alpha-1-antitrypsin	A1AT_HUMAN	SERPINA1
Paeoniflorin	0.6597	Transforming protein RhoA	P61586	RHOA
Paeoniflorin	0.6595	Serine hydroxymethyltransferase, cytosolic	GLYC_HUMAN	SHMT1
Paeoniflorin	0.6574	Complement C1r subcomponent	P00736	C1R
Paeoniflorin	0.6548	Fatty acid-binding protein, adipocyte	FABP4_HUMAN	FABP4
Paeoniflorin	0.6544	Uridine-cytidine kinase 2	Q9BZX2	UCK2
Paeoniflorin	0.6502	Ferrochelatase, mitochondrial	P22830	FECH
Paeoniflorin	0.6483	Cathepsin G	CATG_HUMAN	CTSG
Paeoniflorin	0.645	Ganglioside GM2 activator	P17900	GM2A
Paeoniflorin	0.6441	Prostatic acid phosphatase	PPAP_HUMAN	ACP3
Paeoniflorin	0.6439	Baculoviral IAP repeat-containing protein 4	P98170	XIAP
Paeoniflorin	0.6416	Lactoylglutathione lyase	LGUL_HUMAN	GLO1
Paeoniflorin	0.6412	Protein-arginine deiminase type-4	PADI4_HUMAN	PADI4
Paeoniflorin	0.6409	Coagulation factor XI	FA11_HUMAN	F11
Paeoniflorin	0.6384	Lithostathine-1-alpha	P05451	REG1A
Paeoniflorin	0.6347	Galactosylgalactosylxylosylprotein 3-beta-glucuronosyltransferase 1	B3GA1_HUMAN	B3GAT1
Paeoniflorin	0.6321	Ras-related C3 botulinum toxin substrate 2	P15153	RAC2
Paeoniflorin	0.6271	Phosphoenolpyruvate carboxykinase, cytosolic [GTP]	P35558	PCK1
Paeoniflorin	0.6243	Glucokinase	HXK4_HUMAN	GCK
Paeoniflorin	0.6239	Triosephosphate isomerase	TPIS_HUMAN	TPI1
Paeoniflorin	0.6192	C-C motif chemokine 5	CCL5_HUMAN	CCL5
Paeoniflorin	0.6146	Histamine N-methyltransferase	P50135	HNMT
Paeoniflorin	0.6122	Proto-oncogene tyrosine-protein kinase ABL1	P00519	ABL1
Paeoniflorin	0.6115	Vitamin D3 receptor	VDR_HUMAN	VDR
Paeoniflorin	0.6101	Glucocorticoid receptor	GCR_HUMAN	NR3C1
Paeoniflorin	0.6091	Neprilysin	NEP_HUMAN	MME
Paeoniflorin	0.6088	Heme oxygenase 1	HMOX1_HUMAN	HMOX1
Paeoniflorin	0.6084	ADP-ribosyl cyclase 2	BST1_HUMAN	BST1
Paeoniflorin	0.6048	Transforming growth factor beta-2	TGFB2_HUMAN	TGFB2
Paeoniflorin	0.6042	Angiopoietin-1 receptor	TIE2_HUMAN	TEK
Paeoniflorin	0.6037	Rho GTPase-activating protein 1	Q07960	ARHGAP1
Paeoniflorin	0.6029	Serine/threonine-protein phosphatase PP1-gamma catalytic subunit	P36873	PPP1CC
Paeoniflorin	0.6	Phenylethanolamine N-methyltransferase	PNMT_HUMAN	PNMT
Paeoniflorin	0.5979	Retinol-binding protein 4	RET4_HUMAN	RBP4
Paeoniflorin	0.5955	Endoplasmic reticulum mannosyl-oligosaccharide 1,2-alpha-mannosidase	Q9UKM7	MAN1B1
Paeoniflorin	0.5926	Galectin-2	LEG2_HUMAN	LGALS2
Paeoniflorin	0.5921	Eukaryotic translation initiation factor 4E	P06730	EIF4E
Paeoniflorin	0.5909	Ribonuclease 4	P34096	RNASE4
Paeoniflorin	0.5908	Cellular retinoic acid-binding protein 2	P29373	CRABP2

Paeoniflorin	0.5894	Histone-lysine N-methyltransferase SETD7	SETD7_HUMAN	SETD7
Paeoniflorin	0.589	Tryptophan 5-hydroxylase 1	TPH1_HUMAN	TPH1
Paeoniflorin	0.5883	Serine protease hepsin	P05981	HPN
Paeoniflorin	0.5875	Matrilysin	MMP7_HUMAN	MMP7
Paeoniflorin	0.5849	Copper transport protein ATOX1	O00244	ATOX1
Paeoniflorin	0.5848	Ras-related protein Rab-5A	RAB5A_HUMAN	RAB5A
Paeoniflorin	0.5837	Angiotensin-converting enzyme	ACE_HUMAN	ACE
Paeoniflorin	0.5833	Retinoic acid receptor alpha	RARA_HUMAN	RARA
Paeoniflorin	0.583	Inosine-5-monophosphate dehydrogenase 2	IMDH2_HUMAN	IMPDH2
Paeoniflorin	0.5785	Baculoviral IAP repeat-containing protein 7	Q96CA5	BIRC7
Paeoniflorin	0.5772	Tissue-type plasminogen activator	TPA_HUMAN	PLAT
Paeoniflorin	0.577	Beta-hexosaminidase beta chain	P07686	HEXB
Paeoniflorin	0.5765	Ornithine carbamoyltransferase, mitochondrial	P00480	OTC
Paeoniflorin	0.5759	Medium-chain specific acyl-CoA dehydrogenase, mitochondrial	ACADM_HUMAN	ACADM
Paeoniflorin	0.5746	Proactivator polypeptide	P07602	PSAP
Paeoniflorin	0.5734	L-lactate dehydrogenase B chain	P07195	LDHB
Paeoniflorin	0.5686	Arginase-1	ARGI1_HUMAN	ARG1
Paeoniflorin	0.5681	Heat shock protein HSP 90-beta	P08238	HSP90AB1
Paeoniflorin	0.5672	Mast/stem cell growth factor receptor	KIT_HUMAN	KIT
Paeoniflorin	0.5669	Neuronal calcium sensor 1	NCS1_HUMAN	NCS1
Paeoniflorin	0.5668	Steroid hormone receptor ERR1	P11474	ESRRA
Paeoniflorin	0.5648	L-serine dehydratase	P20132	SDS
Paeoniflorin	0.5556	Galectin-3	LEG3_HUMAN	LGALS3
Paeoniflorin	0.553	Heat shock protein homolog SSE1	P32589	SSE1
Paeoniflorin	0.5529	Eosinophil cationic protein	P12724	RNASE3
Paeoniflorin	0.55	Growth factor receptor-bound protein 2	GRB2_HUMAN	GRB2
Paeoniflorin	0.5496	Pyruvate dehydrogenase E1 component subunit beta, mitochondrial	P11177	PDHB
Paeoniflorin	0.548	Ras-related protein Rab-11A	RB11A_HUMAN	RAB11A
Paeoniflorin	0.5422	Complement component C8 gamma chain	P07360	C8G
Paeoniflorin	0.5419	Adenine phosphoribosyltransferase	P07741	APRT
Paeoniflorin	0.5414	Glucosamine-6-phosphate isomerase	P46926	GNPDA1
Paeoniflorin	0.5411	Caspase-1	CASP1_HUMAN	CASP1
Paeoniflorin	0.5383	Dual specificity mitogen-activated protein kinase kinase 1	MP2K1_HUMAN	MAP2K1
Guaiphenesin	0.9877	Carbonic anhydrase 2	CAH2_HUMAN	CA2
Guaiphenesin	0.9705	Glutathione S-transferase P	GSTP1_HUMAN	GSTP1
Guaiphenesin	0.9571	Triggering receptor expressed on myeloid cells 1	Q9NP99	TREM1
Guaiphenesin	0.9185	Cholinesterase	CHLE_HUMAN	BCHE
Guaiphenesin	0.9163	Purine nucleoside phosphorylase	PNPH_HUMAN	PNP
Guaiphenesin	0.9149	Peptidyl-prolyl cis-trans isomerase A	P62937	PPIA
Guaiphenesin	0.9132	Heat shock protein HSP 90-alpha	P07900	HSP90AA1
Guaiphenesin	0.9023	Carbonic anhydrase 12	CAH12_HUMAN	CA12

Guaiphenesin	0.8957	Cathepsin S	CATS_HUMAN	CTSS
Guaiphenesin	0.8891	cAMP-specific 3,5-cyclic phosphodiesterase 4B	PDE4B_HUMAN	PDE4B
Guaiphenesin	0.8543	Oxysterols receptor LXR-beta	NR1H2_HUMAN	NR1H2
Guaiphenesin	0.8215	Peptidyl-prolyl cis-trans isomerase FKBP1A	FKB1A_HUMAN	FKBP1A
Guaiphenesin	0.8124	Cathepsin K	CATK_HUMAN	CTSK
Guaiphenesin	0.7986	Carbonic anhydrase 1	CAH1_HUMAN	CA1
Guaiphenesin	0.79	Chymase	CMA1_HUMAN	CMA1
Guaiphenesin	0.7735	Death-associated protein kinase 1	DAPK1_HUMAN	DAPK1
Guaiphenesin	0.7597	Cathepsin D	CATD_HUMAN	CTSD
Guaiphenesin	0.7596	Cell division protein kinase 2	P24941	CDK2
Guaiphenesin	0.7477	cAMP-specific 3,5-cyclic phosphodiesterase 4D	PDE4D_HUMAN	PDE4D
Guaiphenesin	0.7446	Complement C1r subcomponent	P00736	C1R
Guaiphenesin	0.7438	Prothrombin	THRB_HUMAN	F2
Guaiphenesin	0.742	Galectin-7	LEG7_HUMAN	LGALS7
Guaiphenesin	0.7368	Beta-secretase 1	BACE1_HUMAN	BACE1
Guaiphenesin	0.7315	Methionine aminopeptidase 1	AMPM1_HUMAN	METAP1
Guaiphenesin	0.7263	Aldose reductase	ALDR_HUMAN	AKR1B1
Guaiphenesin	0.7256	Macrophage migration inhibitory factor	MIF_HUMAN	MIF
Guaiphenesin	0.721	Glycogen phosphorylase, liver form	P06737	PYGL
Guaiphenesin	0.7188	Proto-oncogene serine/threonine-protein kinase Pim-1	PIM1_HUMAN	PIM1
Guaiphenesin	0.7184	Phenylalanine-4-hydroxylase	PH4H_HUMAN	PAH
Guaiphenesin	0.7131	Serine/threonine-protein kinase Chk1	CHK1_HUMAN	CHEK1
Guaiphenesin	0.7028	Ig lambda chain V-II region MGC	P01709	IGLV2-8
Guaiphenesin	0.7006	C-1-tetrahydrofolate synthase, cytoplasmic	P11586	MTHFD1
Guaiphenesin	0.6962	3-phosphoinositide-dependent protein kinase 1	PDPK1_HUMAN	PDPK1
Guaiphenesin	0.6934	Vascular endothelial growth factor receptor 2	VGFR2_HUMAN	KDR
Guaiphenesin	0.6887	Cathepsin B	CATB_HUMAN	CTSB
Guaiphenesin	0.6879	Carnitine O-acetyltransferase	CACP_HUMAN	CRAT
Guaiphenesin	0.6829	Zinc-alpha-2-glycoprotein	ZA2G_HUMAN	AZGP1
Guaiphenesin	0.6829	Serum amyloid P-component	P02743	APCS
Guaiphenesin	0.68	Serine/threonine-protein kinase 6	O14965	AURKA
Guaiphenesin	0.6788	cGMP-specific 3,5-cyclic phosphodiesterase	PDE5A_HUMAN	PDE5A
Guaiphenesin	0.6766	Proto-oncogene tyrosine-protein kinase Src	SRC_HUMAN	SRC
Guaiphenesin	0.6732	Angiogenin	ANGI_HUMAN	ANG
Guaiphenesin	0.6726	Epidermal growth factor receptor	EGFR_HUMAN	EGFR
Guaiphenesin	0.668	Dipeptidyl peptidase 4	DPP4_HUMAN	DPP4
Guaiphenesin	0.6625	cGMP-inhibited 3,5-cyclic phosphodiesterase B	PDE3B_HUMAN	PDE3B
Guaiphenesin	0.6421	Dihydrofolate reductase	DYR_HUMAN	DHFR
Guaiphenesin	0.6288	Inositol monophosphatase	IMPA1_HUMAN	IMPA1
Guaiphenesin	0.6186	Nitric oxide synthase, endothelial	NOS3_HUMAN	NOS3
Guaiphenesin	0.6109	Basic fibroblast growth factor receptor 1	FGFR1_HUMAN	FGFR1
Guaiphenesin	0.6063	Neutrophil collagenase	MMP8_HUMAN	MMP8

Guaiphenesin	0.5992	Proto-oncogene tyrosine-protein kinase LCK	LCK_HUMAN	LCK
Guaiphenesin	0.599	S-methyl-5-thioadenosine phosphorylase	Q13126	MTAP
Guaiphenesin	0.5967	NAD-dependent malic enzyme, mitochondrial	MAOM_HUMAN	ME2
Guaiphenesin	0.5952	Liver carboxylesterase 1	EST1_HUMAN	CES1
Guaiphenesin	0.5925	Lithostathine-1-alpha	P05451	REG1A
Guaiphenesin	0.5904	Lanosterol synthase	ERG7_HUMAN	LSS
Guaiphenesin	0.5889	Activated CDC42 kinase 1	ACK1_HUMAN	TNK2
Guaiphenesin	0.5861	Cytidine deaminase	CDD_HUMAN	CDA
Guaiphenesin	0.586	Carbonyl reductase [NADPH] 1	CBR1_HUMAN	CBR1
Guaiphenesin	0.5847	Urokinase-type plasminogen activator	UROK_HUMAN	PLAU
Guaiphenesin	0.5845	Carboxypeptidase B	P15086	CPB1
Guaiphenesin	0.5842	cAMP-dependent protein kinase catalytic subunit alpha	P00517	PRKACA
Guaiphenesin	0.5832	Neuronal calcium sensor 1	NCS1_HUMAN	NCS1
Guaiphenesin	0.5816	Ephrin type-B receptor 4	EPHB4_HUMAN	EPHB4
Guaiphenesin	0.58	Insulin-like growth factor 1 receptor	IGF1R_HUMAN	IGF1R
Guaiphenesin	0.578	Endoplasmic reticulum mannosyl-oligosaccharide 1,2-alpha-mannosidase	Q9UKM7	MAN1B1
Guaiphenesin	0.5779	Heat shock cognate 71 kDa protein	P11142	HSPA8
Guaiphenesin	0.5776	Hexokinase-1	HXK1_HUMAN	HK1
Guaiphenesin	0.5766	Matrilysin	MMP7_HUMAN	MMP7
Guaiphenesin	0.576	Thymidylate synthase	TYSY_HUMAN	TYMS
Guaiphenesin	0.5732	Methionine aminopeptidase 2	AMPM2_HUMAN	METAP2
Guaiphenesin	0.5709	Peroxisome proliferator-activated receptor gamma	PPARG_HUMAN	PPARG
Guaiphenesin	0.5695	Complement component C8 gamma chain	P07360	C8G
Guaiphenesin	0.5692	Deoxycytidine kinase	DCK_HUMAN	DCK
Guaiphenesin	0.5658	Group 10 secretory phospholipase A2	O15496	PLA2G10
Guaiphenesin	0.5654	S-adenosylmethionine decarboxylase proenzyme	P17707	AMD1
Guaiphenesin	0.5651	Adenosylhomocysteinase	SAHH_HUMAN	AHCY
Guaiphenesin	0.5643	Interferon-stimulated gene 20 kDa protein	ISG20_HUMAN	ISG20
Guaiphenesin	0.5633	Superoxide dismutase [Mn], mitochondrial	P04179	SOD2
Guaiphenesin	0.563	Corticosteroid 11-beta-dehydrogenase isozyme 1	DHI1_HUMAN	HSD11B1
Guaiphenesin	0.5606	GTP-binding protein Rheb	Q15382	RHEB
Guaiphenesin	0.5569	Baculoviral IAP repeat-containing protein 4	XIAP_HUMAN	XIAP
Guaiphenesin	0.5561	Serine/threonine-protein kinase PAK 7	PAK7_HUMAN	PAK7
Guaiphenesin	0.5555	Mitogen-activated protein kinase 14	Q16539	MAPK14
Guaiphenesin	0.5541	Transforming protein RhoA	P61586	RHOA
Guaiphenesin	0.5539	Inosine-5-monophosphate dehydrogenase 2	IMDH2_HUMAN	IMPDH2
Guaiphenesin	0.5511	Caspase-7	CASP7_HUMAN	CASP7
Guaiphenesin	0.5489	Tyrosine-protein phosphatase non-receptor type 1	PTN1_HUMAN	PTPN1
Guaiphenesin	0.5486	Ornithine carbamoyltransferase, mitochondrial	P00480	OTC
Guaiphenesin	0.5472	Alcohol dehydrogenase class-3	ADHX_HUMAN	ADH5
Guaiphenesin	0.5465	Coagulation factor X	P00742	F10
Guaiphenesin	0.545	Pancreatic alpha-amylase	AMYP_HUMAN	AMY2A

Guaiphenesin	0.5406	Glycogen synthase kinase-3 beta	P49841	GSK3B
Guaiphenesin	0.5394	Tyrosine-protein kinase ITK/TSK	Q08881	ITK
Guaiphenesin	0.5393	Androgen receptor	ANDR_HUMAN	AR
Guaiphenesin	0.5375	L-lactate dehydrogenase B chain	P07195	LDHB
Guaiphenesin	0.5303	Stromelysin-1	MMP3_HUMAN	MMP3
Guaiphenesin	0.5259	Adenosine kinase	ADK_HUMAN	ADK
Guaiphenesin	0.5245	Tyrosine-protein kinase HCK	HCK_HUMAN	HCK
Guaiphenesin	0.5162	Aldo-keto reductase family 1 member C3	AK1C3_HUMAN	AKR1C3
Guaiphenesin	0.5124	Caspase-3	CASP3_HUMAN	CASP3
Guaiphenesin	0.5086	Tryptophan 5-hydroxylase 1	TPH1_HUMAN	TPH1
Guaiphenesin	0.5061	Galectin-3	LEG3_HUMAN	LGALS3
Guaiphenesin	0.5019	Cyclin-A2	CCNA2_HUMAN	CCNA2
Naringenin-7-O-glucoside	0.9861	ADP-ribose pyrophosphatase, mitochondrial	Q9BW91	NUDT9
Naringenin-7-O-glucoside	0.9794	Tyrosine-protein kinase HCK	HCK_HUMAN	HCK
Naringenin-7-O-glucoside	0.9765	Seprase	SEPR_HUMAN	FAP
Naringenin-7-O-glucoside	0.9764	Peptidyl-prolyl cis-trans isomerase A	P62937	PPIA
Naringenin-7-O-glucoside	0.975	Carbonic anhydrase 2	CAH2_HUMAN	CA2
Naringenin-7-O-glucoside	0.9725	Estrogen receptor	ESR1_HUMAN	ESR1
Naringenin-7-O-glucoside	0.9718	Progesterone receptor	PRGR_HUMAN	PGR
Naringenin-7-O-glucoside	0.9708	TGF-beta receptor type-2	TGFR2_HUMAN	TGFBR2
Naringenin-7-O-glucoside	0.9691	Methionine aminopeptidase 1	AMPM1_HUMAN	METAP1
Naringenin-7-O-glucoside	0.967	Carbonic anhydrase 1	CAH1_HUMAN	CA1
Naringenin-7-O-glucoside	0.9664	Serine/threonine-protein kinase Chk1	CHK1_HUMAN	CHEK1
Naringenin-7-O-glucoside	0.9652	Transthyretin	TTHY_HUMAN	TTR
Naringenin-7-O-glucoside	0.9648	Epidermal growth factor receptor	EGFR_HUMAN	EGFR
Naringenin-7-O-glucoside	0.9636	Cholinesterase	CHLE_HUMAN	BCHE
Naringenin-7-O-glucoside	0.961	S-methyl-5-thioadenosine phosphorylase	Q13126	MTAP
Naringenin-7-O-glucoside	0.96	Glutathione S-transferase P	GSTP1_HUMAN	GSTP1

Naringenin-7-O-glucoside	0.9582	Carbonic anhydrase 12	CAH12_HUMAN	CA12
Naringenin-7-O-glucoside	0.9578	Cyclin-A2	CCNA2_HUMAN	CCNA2
Naringenin-7-O-glucoside	0.9564	Cathepsin S	CATS_HUMAN	CTSS
Naringenin-7-O-glucoside	0.954	Prothrombin	THRB_HUMAN	F2
Naringenin-7-O-glucoside	0.9503	Chymase	CMA1_HUMAN	CMA1
Naringenin-7-O-glucoside	0.9498	Triggering receptor expressed on myeloid cells 1	Q9NP99	TREM1
Naringenin-7-O-glucoside	0.9481	Purine nucleoside phosphorylase	PNPH_HUMAN	PNP
Naringenin-7-O-glucoside	0.9463	Oxysterols receptor LXR-beta	NR1H2_HUMAN	NR1H2
Naringenin-7-O-glucoside	0.9422	Proto-oncogene serine/threonine-protein kinase Pim-1	PIM1_HUMAN	PIM1
Naringenin-7-O-glucoside	0.9339	Cell division protein kinase 2	P24941	CDK2
Naringenin-7-O-glucoside	0.9313	Cathepsin D	CATD_HUMAN	CTSD
Naringenin-7-O-glucoside	0.9304	Cytochrome P450 19A1	P11511	CYP19A1
Naringenin-7-O-glucoside	0.9284	Heat shock protein HSP 90-alpha	P07900	HSP90AA1
Naringenin-7-O-glucoside	0.9244	Inositol monophosphatase	IMPA1_HUMAN	IMPA1
Naringenin-7-O-glucoside	0.9195	Androgen receptor	ANDR_HUMAN	AR
Naringenin-7-O-glucoside	0.9194	cAMP-specific 3,5-cyclic phosphodiesterase 4B	PDE4B_HUMAN	PDE4B
Naringenin-7-O-glucoside	0.9184	Heat shock cognate 71 kDa protein	P11142	HSPA8
Naringenin-7-O-glucoside	0.9114	Urokinase-type plasminogen activator	UROK_HUMAN	PLAU
Naringenin-7-O-glucoside	0.9063	Immunoglobulin alpha Fc receptor	FCAR_HUMAN	FCAR
Naringenin-7-O-glucoside	0.9053	Atrial natriuretic peptide clearance receptor	ANPRC_HUMAN	NPR3
Naringenin-7-O-glucoside	0.9043	Aldo-keto reductase family 1 member C3	AK1C3_HUMAN	AKR1C3
Naringenin-7-O-glucoside	0.8997	Reticulon-4 receptor	RTN4R_HUMAN	RTN4R
Naringenin-7-O-glucoside	0.8995	Cell division protein kinase 6	CDK6_HUMAN	CDK6

Naringenin-7-O-glucoside	0.896	Cathepsin L2	CATL2_HUMAN	CTSV
Naringenin-7-O-glucoside	0.892	Probable ATP-dependent RNA helicase DDX6	DDX6_HUMAN	DDX6
Naringenin-7-O-glucoside	0.8844	Serum albumin	ALBU_HUMAN	ALB
Naringenin-7-O-glucoside	0.8822	Steryl-sulfatase	STS_HUMAN	STS
Naringenin-7-O-glucoside	0.8821	Apolipoprotein A-II	APOA2_HUMAN	APOA2
Naringenin-7-O-glucoside	0.8654	Estrogen receptor beta	ESR2_HUMAN	ESR2
Naringenin-7-O-glucoside	0.863	Peptidyl-prolyl cis-trans isomerase FKBP1A	FKB1A_HUMAN	FKBP1A
Naringenin-7-O-glucoside	0.8454	Mitogen-activated protein kinase 10	MK10_HUMAN	MAPK10
Naringenin-7-O-glucoside	0.8361	Caspase-3	CASP3_HUMAN	CASP3
Naringenin-7-O-glucoside	0.8311	Glutathione reductase, mitochondrial	P00390	GSR
Naringenin-7-O-glucoside	0.8293	cGMP-inhibited 3,5-cyclic phosphodiesterase B	PDE3B_HUMAN	PDE3B
Naringenin-7-O-glucoside	0.8214	Hexokinase-1	HXK1_HUMAN	HK1
Naringenin-7-O-glucoside	0.8088	Zinc-alpha-2-glycoprotein	ZA2G_HUMAN	AZGP1
Naringenin-7-O-glucoside	0.8055	Complement factor B	CFAB_HUMAN	CFB
Naringenin-7-O-glucoside	0.8021	Beta-secretase 1	BACE1_HUMAN	BACE1
Naringenin-7-O-glucoside	0.7852	Estradiol 17-beta-dehydrogenase 11	DHB11_HUMAN	HSD17B11
Naringenin-7-O-glucoside	0.778	Intercellular adhesion molecule 2	ICAM2_HUMAN	ICAM2
Naringenin-7-O-glucoside	0.7777	Lanosterol synthase	ERG7_HUMAN	LSS
Naringenin-7-O-glucoside	0.7696	Mitogen-activated protein kinase 8	MK08_HUMAN	MAPK8
Naringenin-7-O-glucoside	0.7661	Galactokinase	GALK1_HUMAN	GALK1
Naringenin-7-O-glucoside	0.7571	Vascular endothelial growth factor receptor 2	VGFR2_HUMAN	KDR
Naringenin-7-O-glucoside	0.7533	Amine oxidase [flavin-containing] B	AOFB_HUMAN	MAOB
Naringenin-7-O-glucoside	0.75	Superoxide dismutase [Mn], mitochondrial	P04179	SOD2

Naringenin-7-O-glucoside	0.7433	Angiogenin	ANGI_HUMAN	ANG
Naringenin-7-O-glucoside	0.7428	Bone morphogenetic protein 7	BMP7_HUMAN	BMP7
Naringenin-7-O-glucoside	0.7416	Nitric oxide synthase, endothelial	NOS3_HUMAN	NOS3
Naringenin-7-O-glucoside	0.7416	Branched-chain-amino-acid aminotransferase, mitochondrial	O15382	BCAT2
Naringenin-7-O-glucoside	0.7409	Aldose reductase	ALDR_HUMAN	AKR1B1
Naringenin-7-O-glucoside	0.7402	Sex hormone-binding globulin	SHBG_HUMAN	SHBG
Naringenin-7-O-glucoside	0.7355	Dihydrofolate reductase	DYR_HUMAN	DHFR
Naringenin-7-O-glucoside	0.7352	Ephrin type-B receptor 4	EPHB4_HUMAN	EPHB4
Naringenin-7-O-glucoside	0.7314	Macrophage migration inhibitory factor	MIF_HUMAN	MIF
Naringenin-7-O-glucoside	0.7306	ADAM 17	ADA17_HUMAN	ADAM17
Naringenin-7-O-glucoside	0.7301	GTP-binding protein Rheb	Q15382	RHEB
Naringenin-7-O-glucoside	0.7301	Glycogen phosphorylase, liver form	P06737	PYGL
Naringenin-7-O-glucoside	0.7271	cAMP-specific 3,5-cyclic phosphodiesterase 4D	PDE4D_HUMAN	PDE4D
Naringenin-7-O-glucoside	0.7253	Cathepsin B	CATB_HUMAN	CTSB
Naringenin-7-O-glucoside	0.7236	Activated CDC42 kinase 1	ACK1_HUMAN	TNK2
Naringenin-7-O-glucoside	0.723	Proto-oncogene tyrosine-protein kinase Src	SRC_HUMAN	SRC
Naringenin-7-O-glucoside	0.7221	Ig lambda chain V-II region MGC	P01709	IGLV2-8
Naringenin-7-O-glucoside	0.7213	Carbonyl reductase [NADPH] 1	CBR1_HUMAN	CBR1
Naringenin-7-O-glucoside	0.7211	Estrogen-related receptor gamma	P62508	ESRRG
Naringenin-7-O-glucoside	0.7209	Neuronal calcium sensor 1	NCS1_HUMAN	NCS1
Naringenin-7-O-glucoside	0.7206	Mitogen-activated protein kinase 14	Q16539	MAPK14
Naringenin-7-O-glucoside	0.7195	cGMP-specific 3,5-cyclic phosphodiesterase	PDE5A_HUMAN	PDE5A
Naringenin-7-O-glucoside	0.7176	S-adenosylmethionine decarboxylase proenzyme	DCAM_HUMAN	AMD1

Naringenin-7-O-glucoside	0.7157	3-phosphoinositide-dependent protein kinase 1	PDPK1_HUMAN	PDPK1
Naringenin-7-O-glucoside	0.7148	Thymidine phosphorylase	TYPH_HUMAN	TYMP
Naringenin-7-O-glucoside	0.7148	Death-associated protein kinase 1	DAPK1_HUMAN	DAPK1
Naringenin-7-O-glucoside	0.7144	Pyridoxine-5-phosphate oxidase	Q9NVS9	PNPO
Naringenin-7-O-glucoside	0.7137	Kinesin-like protein KIF11	KIF11_HUMAN	KIF11
Naringenin-7-O-glucoside	0.7121	C-1-tetrahydrofolate synthase, cytoplasmic	P11586	MTHFD1
Naringenin-7-O-glucoside	0.7086	Bis(5-adenosyl)-triphosphatase	P49789	FHIT
Naringenin-7-O-glucoside	0.7086	Bile salt sulfotransferase	Q06520	SULT2A1
Naringenin-7-O-glucoside	0.7082	Sorbitol dehydrogenase	DHSO_HUMAN	SORD
Naringenin-7-O-glucoside	0.7071	Annexin A5	ANXA5_HUMAN	ANXA5
Naringenin-7-O-glucoside	0.7063	Dihydroorotate dehydrogenase, mitochondrial	PYRD_HUMAN	DHODH
Naringenin-7-O-glucoside	0.7056	Galactosylgalactosylxylosylprotein 3-beta-glucuronosyltransferase 1	B3GA1_HUMAN	B3GAT1
Naringenin-7-O-glucoside	0.7054	Peroxisome proliferator-activated receptor gamma	PPARG_HUMAN	PPARG
Naringenin-7-O-glucoside	0.7053	Troponin C, slow skeletal and cardiac muscles	P63316	TNNC1
Naringenin-7-O-glucoside	0.7027	Casein kinase I isoform gamma-2	KC1G2_HUMAN	CSNK1G2
Naringenin-7-O-glucoside	0.7016	Collagenase 3	MMP13_HUMAN	MMP13
Naringenin-7-O-glucoside	0.7008	Chitotriosidase-1	Q13231	CHIT1
Naringenin-7-O-glucoside	0.6995	Carnitine O-acetyltransferase	CACP_HUMAN	CRAT
Naringenin-7-O-glucoside	0.6986	Beta-hexosaminidase beta chain	P07686	HEXB
Naringenin-7-O-glucoside	0.698	Estradiol 17-beta-dehydrogenase 1	P14061	HSD17B1
Naringenin-7-O-glucoside	0.6947	Peroxisome proliferator-activated receptor delta	Q03181	PPARD
Naringenin-7-O-glucoside	0.6923	Dipeptidyl peptidase 4	DPP4_HUMAN	DPP4
Naringenin-7-O-glucoside	0.6921	Protein farnesyltransferase/geranylgeranyltransferase type-1 subunit alpha	FNTA_HUMAN	FNTA

Naringenin-7-O-glucoside	0.6909	Aldehyde dehydrogenase, mitochondrial	ALDH2_HUMAN	ALDH2
Naringenin-7-O-glucoside	0.6855	RAC-alpha serine/threonine-protein kinase	AKT1_HUMAN	AKT1
Naringenin-7-O-glucoside	0.6834	Glutaminyl-peptide cyclotransferase	QPCT_HUMAN	QPCT
Naringenin-7-O-glucoside	0.6788	Insulin-like growth factor 1 receptor	P08069	IGF1R
Naringenin-7-O-glucoside	0.6682	Corticosteroid 11-beta-dehydrogenase isozyme 1	P28845	HSD11B1
Naringenin-7-O-glucoside	0.6666	Thymidylate synthase	TYSY_HUMAN	TYMS
Naringenin-7-O-glucoside	0.6661	Caspase-7	CASP7_HUMAN	CASP7
Naringenin-7-O-glucoside	0.666	Ribosyldihydronicotinamide dehydrogenase [quinone]	NQO2_HUMAN	NQO2
Naringenin-7-O-glucoside	0.6657	Cathepsin K	CATK_HUMAN	CTSK
Naringenin-7-O-glucoside	0.6651	3-hydroxy-3-methylglutaryl-coenzyme A reductase	HMDH_HUMAN	HMGCR
Naringenin-7-O-glucoside	0.6645	Glycogen synthase kinase-3 beta	GSK3B_HUMAN	GSK3B
Naringenin-7-O-glucoside	0.6638	BAG family molecular chaperone regulator 1	BAG1_HUMAN	BAG1
Naringenin-7-O-glucoside	0.6635	Coagulation factor X	FA10_HUMAN	F10
Naringenin-7-O-glucoside	0.6632	Complement C1r subcomponent	P00736	C1R
Naringenin-7-O-glucoside	0.6607	Galectin-7	LEG7_HUMAN	LGALS7
Naringenin-7-O-glucoside	0.6597	Tyrosine-protein phosphatase non-receptor type 1	PTN1_HUMAN	PTPN1
Naringenin-7-O-glucoside	0.6591	Stromelysin-1	MMP3_HUMAN	MMP3
Naringenin-7-O-glucoside	0.659	Alcohol dehydrogenase 1B	ADH1B_HUMAN	ADH1B
Naringenin-7-O-glucoside	0.6588	Dual specificity protein phosphatase 6	DUS6_HUMAN	DUSP6
Naringenin-7-O-glucoside	0.6569	Liver carboxylesterase 1	EST1_HUMAN	CES1
Naringenin-7-O-glucoside	0.6564	Mineralocorticoid receptor	MCR_HUMAN	NR3C2
Naringenin-7-O-glucoside	0.6545	Lactoylglutathione lyase	LGUL_HUMAN	GLO1
Naringenin-7-O-glucoside	0.6538	NAD(P)H dehydrogenase [quinone] 1	NQO1_HUMAN	NQO1

Naringenin-7-O-glucoside	0.653	cAMP-dependent protein kinase catalytic subunit alpha	P00517	PRKACA
Naringenin-7-O-glucoside	0.6505	Leukotriene A-4 hydrolase	LKHA4_HUMAN	LTA4H
Naringenin-7-O-glucoside	0.6478	Phosphoenolpyruvate carboxykinase, cytosolic [GTP]	P35558	PCK1
Naringenin-7-O-glucoside	0.647	Vitamin D-binding protein	VTDB_HUMAN	GC
Naringenin-7-O-glucoside	0.6451	Phenylalanine-4-hydroxylase	PH4H_HUMAN	PAH
Naringenin-7-O-glucoside	0.6434	Hepatocyte growth factor receptor	MET_HUMAN	MET
Naringenin-7-O-glucoside	0.6393	Adenosylhomocysteinase	SAHH_HUMAN	AHCY
Naringenin-7-O-glucoside	0.6389	Ras-related C3 botulinum toxin substrate 2	P15153	RAC2
Naringenin-7-O-glucoside	0.6387	Renin	RENI_HUMAN	REN
Naringenin-7-O-glucoside	0.637	Histo-blood group ABO system transferase	P16442	ABO
Naringenin-7-O-glucoside	0.637	Complement factor D	CFAD_HUMAN	CFD
Naringenin-7-O-glucoside	0.632	Triosephosphate isomerase	TPIS_HUMAN	TPI1
Naringenin-7-O-glucoside	0.6319	Transforming protein RhoA	P61586	RHOA
Naringenin-7-O-glucoside	0.631	Galectin-3	LEG3_HUMAN	LGALS3
Naringenin-7-O-glucoside	0.63	Alpha-1-antitrypsin	A1AT_HUMAN	SERPINA1
Naringenin-7-O-glucoside	0.6289	Lithostathine-1-alpha	P05451	REG1A
Naringenin-7-O-glucoside	0.6269	Insulin-like growth factor IA	P01343	IGF1
Naringenin-7-O-glucoside	0.6267	Interferon-stimulated gene 20 kDa protein	ISG20_HUMAN	ISG20
Naringenin-7-O-glucoside	0.6248	Neutrophil collagenase	MMP8_HUMAN	MMP8
Naringenin-7-O-glucoside	0.6223	Deoxycytidine kinase	DCK_HUMAN	DCK
Naringenin-7-O-glucoside	0.6205	Nicotinamide mononucleotide adenylyltransferase 3	Q96T66	NMNAT3
Naringenin-7-O-glucoside	0.6157	Galectin-2	LEG2_HUMAN	LGALS2
Naringenin-7-O-glucoside	0.6141	Serine/threonine-protein kinase PLK1	P53350	PLK1

Naringenin-7-O-glucoside	0.606	ADP-ribosyl cyclase 2	BST1_HUMAN	BST1
Naringenin-7-O-glucoside	0.6042	Uridine 5-monophosphate synthase	P11172	UMPS
Naringenin-7-O-glucoside	0.5991	Leukocyte elastase	ELNE_HUMAN	ELANE
Naringenin-7-O-glucoside	0.5989	Macrophage metalloelastase	MMP12_HUMAN	MMP12
Naringenin-7-O-glucoside	0.5961	C-C motif chemokine 5	CCL5_HUMAN	CCL5
Naringenin-7-O-glucoside	0.5958	Endoplasmic reticulum mannosyl-oligosaccharide 1,2-alpha-mannosidase	Q9UKM7	MAN1B1
Naringenin-7-O-glucoside	0.5947	Pancreatic alpha-amylase	AMYP_HUMAN	AMY2A
Naringenin-7-O-glucoside	0.5941	Ornithine carbamoyltransferase, mitochondrial	P00480	OTC
Naringenin-7-O-glucoside	0.5936	Beta-hexosaminidase subunit beta	P07686	HEXB
Naringenin-7-O-glucoside	0.5928	Uridine-cytidine kinase 2	Q9BZX2	UCK2
Naringenin-7-O-glucoside	0.5901	Tyrosine-protein kinase SYK	KSYK_HUMAN	SYK
Naringenin-7-O-glucoside	0.59	TGF-beta receptor type-1	TGFR1_HUMAN	TGFBR1
Naringenin-7-O-glucoside	0.5895	Tyrosyl-tRNA synthetase, cytoplasmic	P54577	YARS1
Naringenin-7-O-glucoside	0.5891	Proto-oncogene tyrosine-protein kinase LCK	LCK_HUMAN	LCK
Naringenin-7-O-glucoside	0.5882	Cyclin-T1	CCNT1_HUMAN	CCNT1
Naringenin-7-O-glucoside	0.587	Protein-glutamine gamma-glutamyltransferase 2	TGM2_HUMAN	TGM2
Naringenin-7-O-glucoside	0.5866	Prostatic acid phosphatase	PPAP_HUMAN	ACP3
Naringenin-7-O-glucoside	0.5862	Alcohol dehydrogenase 1C	P00326	ADH1C
Naringenin-7-O-glucoside	0.5854	Copper transport protein ATOX1	O00244	ATOX1
Naringenin-7-O-glucoside	0.5835	NAD-dependent malic enzyme, mitochondrial	MAOM_HUMAN	ME2
Naringenin-7-O-glucoside	0.5826	Phospholipase A2, membrane associated	P14555	PLA2G2A
Naringenin-7-O-glucoside	0.5826	Fatty acid-binding protein, epidermal	FABP5_HUMAN	FABP5
Naringenin-7-O-glucoside	0.5814	Pyruvate dehydrogenase E1 component subunit beta, mitochondrial	P11177	PDHB

Naringenin-7-O-glucoside	0.5785	Cytidine deaminase	CDD_HUMAN	CDA
Naringenin-7-O-glucoside	0.5773	Tyrosine-protein phosphatase non-receptor type 11	PTN11_HUMAN	PTPN11
Naringenin-7-O-glucoside	0.5769	Cathepsin G	CATG_HUMAN	CTSG
Naringenin-7-O-glucoside	0.5764	Rho GTPase-activating protein 1	Q07960	ARHGAP1
Naringenin-7-O-glucoside	0.5764	Aldo-keto reductase family 1 member C1	Q04828	AKR1C1
Naringenin-7-O-glucoside	0.5752	Coagulation factor VII	FA7_HUMAN	F7
Naringenin-7-O-glucoside	0.575	Disintegrin and metalloproteinase domain-containing protein 17	P78536	ADAM17
Naringenin-7-O-glucoside	0.5738	Histone deacetylase 8	Q9BY41	HDAC8
Naringenin-7-O-glucoside	0.5736	Catenin alpha-1	CTNA1_HUMAN	CTNNA1
Naringenin-7-O-glucoside	0.5713	Phosphatidylinositol-4,5-bisphosphate 3-kinase catalytic subunit gamma isoform	PK3CG_HUMAN	PIK3CG
Naringenin-7-O-glucoside	0.5704	Retinoic acid receptor RXR-alpha	RXRA_HUMAN	RXRA
Naringenin-7-O-glucoside	0.5696	Ribonuclease 4	P34096	RNASE4
Naringenin-7-O-glucoside	0.5689	Tyrosine-protein kinase JAK3	JAK3_HUMAN	JAK3
Naringenin-7-O-glucoside	0.5679	Casein kinase II subunit alpha	P68400	CSNK2A1
Naringenin-7-O-glucoside	0.5661	Inosine-5-monophosphate dehydrogenase 2	IMDH2_HUMAN	IMPDH2
Naringenin-7-O-glucoside	0.5649	Tyrosine-protein kinase ZAP-70	ZAP70_HUMAN	ZAP70
Naringenin-7-O-glucoside	0.5641	Protein-glutamine gamma-glutamyltransferase E	TGM3_HUMAN	TGM3
Naringenin-7-O-glucoside	0.5633	Heat shock protein homolog SSE1	P32589	SSE1
Naringenin-7-O-glucoside	0.5625	Basic fibroblast growth factor receptor 1	FGFR1_HUMAN	FGFR1
Naringenin-7-O-glucoside	0.5617	Epoxide hydrolase 2	HYES_HUMAN	EPHX2
Naringenin-7-O-glucoside	0.56	Serine protease hepsin	P05981	HPN
Naringenin-7-O-glucoside	0.5589	Methionine aminopeptidase 2	AMPM2_HUMAN	METAP2
Naringenin-7-O-glucoside	0.5579	Tyrosine-protein kinase ITK/TSK	Q08881	ITK

Naringenin-7-O-glucoside	0.5578	Matrilysin	MMP7_HUMAN	MMP7
Naringenin-7-O-glucoside	0.5563	Eosinophil cationic protein	P12724	RNASE3
Naringenin-7-O-glucoside	0.553	Group 10 secretory phospholipase A2	O15496	PLA2G10
Naringenin-7-O-glucoside	0.5458	UDP-glucose 4-epimerase	GALE_HUMAN	GALE
Naringenin-7-O-glucoside	0.5455	Serum amyloid P-component	P02743	APCS
Naringenin-7-O-glucoside	0.5454	Alcohol dehydrogenase class-3	ADHX_HUMAN	ADH5
Naringenin-7-O-glucoside	0.5443	Peroxisome proliferator-activated receptor alpha	PPARA_HUMAN	PPARA
Naringenin-7-O-glucoside	0.5435	L-lactate dehydrogenase B chain	P07195	LDHB
Naringenin-7-O-glucoside	0.5429	Nitric oxide synthase, inducible	NOS2_HUMAN	NOS2
Naringenin-7-O-glucoside	0.5426	Glucosamine-6-phosphate isomerase	P46926	GNPDA1
Naringenin-7-O-glucoside	0.5416	Poly [ADP-ribose] polymerase 1	P09874	PARP1
Naringenin-7-O-glucoside	0.5406	Complement component C8 gamma chain	P07360	C8G
Naringenin-7-O-glucoside	0.5378	Kynurenine--oxoglutarate transaminase 1	KAT1_HUMAN	KYAT1
Naringenin-7-O-glucoside	0.5323	Serine/threonine-protein kinase PAK 7	PAK7_HUMAN	PAK7
Naringenin-7-O-glucoside	0.5304	Glutathione S-transferase A1	P08263	GSTA1
Naringenin-7-O-glucoside	0.5297	Glucose-6-phosphate isomerase	G6PI_HUMAN	GPI
Naringenin-7-O-glucoside	0.5296	Tyrosine-protein kinase JAK2	JAK2_HUMAN	JAK2
Naringenin-7-O-glucoside	0.527	MAP kinase-activated protein kinase 2	P49137	MAPKAPK2
Naringenin-7-O-glucoside	0.5268	Dual specificity protein kinase CLK1	CLK1_HUMAN	CLK1
Naringenin-7-O-glucoside	0.5266	E3 ubiquitin-protein ligase Mdm2	MDM2_HUMAN	MDM2
Naringenin-7-O-glucoside	0.52	Glutathione S-transferase theta-2	GSTT2_HUMAN	GSTT2BGSTT2
Naringenin-7-O-glucoside	0.5155	Tryptophan 5-hydroxylase 1	TPH1_HUMAN	TPH1
Naringenin-7-O-glucoside	0.5117	Trafficking protein particle complex subunit 3	O43617	TRAPPC3

Naringenin-7-O-glucoside	0.5096	Adenosine kinase	ADK_HUMAN	ADK
Naringenin-7-O-glucoside	0.509	SEC14-like protein 2	O76054	SEC14L2
Naringenin-7-O-glucoside	0.5084	Spermidine synthase	P19623	SRM
Naringenin-7-O-glucoside	0.5052	Bifunctional 3-phosphoadenosine 5-phosphosulfate synthetase 1	O43252	PAPSS1
Naringenin-7-O-glucoside	0.5046	B transferase	Q9NY01	ABO
(+)-Paeonilactone C	0.9852	Estrogen receptor	ESR1_HUMAN	ESR1
(+)-Paeonilactone C	0.9778	Chymase	CMA1_HUMAN	CMA1
(+)-Paeonilactone C	0.9759	Glutathione S-transferase P	GSTP1_HUMAN	GSTP1
(+)-Paeonilactone C	0.9735	Triggering receptor expressed on myeloid cells 1	Q9NP99	TREM1
(+)-Paeonilactone C	0.9711	Cholinesterase	CHLE_HUMAN	BCHE
(+)-Paeonilactone C	0.966	Purine nucleoside phosphorylase	PNPH_HUMAN	PNP
(+)-Paeonilactone C	0.9648	Oxysterols receptor LXR-beta	NR1H2_HUMAN	NR1H2
(+)-Paeonilactone C	0.9631	Epidermal growth factor receptor	EGFR_HUMAN	EGFR
(+)-Paeonilactone C	0.959	Probable ATP-dependent RNA helicase DDX6	DDX6_HUMAN	DDX6
(+)-Paeonilactone C	0.9553	Stromelysin-1	MMP3_HUMAN	MMP3
(+)-Paeonilactone C	0.9549	Peptidyl-prolyl cis-trans isomerase A	P62937	PPIA
(+)-Paeonilactone C	0.954	Mitogen-activated protein kinase 10	MK10_HUMAN	MAPK10
(+)-Paeonilactone C	0.9441	Cyclin-dependent kinase 5 activator 1	CD5R1_HUMAN	CDK5R1
(+)-Paeonilactone C	0.9359	Mitogen-activated protein kinase 8	MK08_HUMAN	MAPK8
(+)-Paeonilactone C	0.9342	Proto-oncogene serine/threonine-protein kinase Pim-1	PIM1_HUMAN	PIM1
(+)-Paeonilactone C	0.931	Peptidyl-prolyl cis-trans isomerase FKBP1A	FKB1A_HUMAN	FKBP1A
(+)-Paeonilactone C	0.9254	cAMP-specific 3,5-cyclic phosphodiesterase 4B	PDE4B_HUMAN	PDE4B
(+)-Paeonilactone C	0.9129	Liver carboxylesterase 1	EST1_HUMAN	CES1
(+)-Paeonilactone C	0.9119	Complement factor B	CFAB_HUMAN	CFB
(+)-Paeonilactone C	0.8993	Prothrombin	THRB_HUMAN	F2
(+)-Paeonilactone C	0.8878	Transthyretin	TTHY_HUMAN	TTR
(+)-Paeonilactone C	0.8635	Heat shock cognate 71 kDa protein	P11142	HSPA8
(+)-Paeonilactone C	0.8552	Cathepsin B	CATB_HUMAN	CTSB
(+)-Paeonilactone C	0.8288	Carbonic anhydrase 2	CAH2_HUMAN	CA2
(+)-Paeonilactone C	0.7567	Cathepsin D	CATD_HUMAN	CTSD
(+)-Paeonilactone C	0.7521	Cell division protein kinase 2	P24941	CDK2
(+)-Paeonilactone C	0.7488	Heat shock protein HSP 90-alpha	HS90A_HUMAN	HSP90AA1
(+)-Paeonilactone C	0.7479	Basic fibroblast growth factor receptor 1	FGFR1_HUMAN	FGFR1
(+)-Paeonilactone C	0.746	Kinesin-like protein KIF11	KIF11_HUMAN	KIF11
(+)-Paeonilactone C	0.7454	Angiogenin	ANGI_HUMAN	ANG
(+)-Paeonilactone C	0.7434	Protein farnesyltransferase/geranylgeranyltransferase type-1 subunit alpha	FNTA_HUMAN	FNTA
(+)-Paeonilactone C	0.7426	Peroxisome proliferator-activated receptor delta	Q03181	PPARD
(+)-Paeonilactone C	0.7421	Coagulation factor X	FA10_HUMAN	F10

(+)-Paeonilactone C	0.7418	Aldose reductase	ALDR_HUMAN	AKR1B1
(+)-Paeonilactone C	0.7413	Mitogen-activated protein kinase 14	Q16539	MAPK14
(+)-Paeonilactone C	0.7411	cGMP-inhibited 3,5-cyclic phosphodiesterase B	PDE3B_HUMAN	PDE3B
(+)-Paeonilactone C	0.7405	Macrophage migration inhibitory factor	MIF_HUMAN	MIF
(+)-Paeonilactone C	0.7401	Urokinase-type plasminogen activator	UROK_HUMAN	PLAU
(+)-Paeonilactone C	0.7395	Collagenase 3	MMP13_HUMAN	MMP13
(+)-Paeonilactone C	0.7384	Hexokinase-1	HXK1_HUMAN	HK1
(+)-Paeonilactone C	0.7379	S-methyl-5-thioadenosine phosphorylase	Q13126	MTAP
(+)-Paeonilactone C	0.7369	3-phosphoinositide-dependent protein kinase 1	PDPK1_HUMAN	PDPK1
(+)-Paeonilactone C	0.7353	C-1-tetrahydrofolate synthase, cytoplasmic	P11586	MTHFD1
(+)-Paeonilactone C	0.7345	Pyridoxine-5-phosphate oxidase	Q9NVS9	PNPO
(+)-Paeonilactone C	0.7338	Serine/threonine-protein kinase Chk1	CHK1_HUMAN	CHEK1
(+)-Paeonilactone C	0.7322	Neutrophil collagenase	MMP8_HUMAN	MMP8
(+)-Paeonilactone C	0.7309	Galectin-7	LEG7_HUMAN	LGALS7
(+)-Paeonilactone C	0.7306	cGMP-specific 3,5-cyclic phosphodiesterase	PDE5A_HUMAN	PDE5A
(+)-Paeonilactone C	0.7285	Serum amyloid P-component	P02743	APCS
(+)-Paeonilactone C	0.726	S-adenosylmethionine decarboxylase proenzyme	DCAM_HUMAN	AMD1
(+)-Paeonilactone C	0.7237	Vascular endothelial growth factor receptor 2	VGFR2_HUMAN	KDR
(+)-Paeonilactone C	0.7219	Complement factor D	CFAD_HUMAN	CFD
(+)-Paeonilactone C	0.7195	Estrogen receptor beta	Q62986	ESR2
(+)-Paeonilactone C	0.7101	Proto-oncogene tyrosine-protein kinase Src	SRC_HUMAN	SRC
(+)-Paeonilactone C	0.7071	Bone morphogenetic protein 7	BMP7_HUMAN	BMP7
(+)-Paeonilactone C	0.7063	Cyclin-A2	CCNA2_HUMAN	CCNA2
(+)-Paeonilactone C	0.7043	Carbonyl reductase [NADPH] 1	CBR1_HUMAN	CBR1
(+)-Paeonilactone C	0.6943	Interferon-stimulated gene 20 kDa protein	ISG20_HUMAN	ISG20
(+)-Paeonilactone C	0.6873	Cathepsin K	CATK_HUMAN	CTSK
(+)-Paeonilactone C	0.6871	Estrogen-related receptor gamma	P62508	ESRRG
(+)-Paeonilactone C	0.6862	NAD(P)H dehydrogenase [quinone] 1	NQO1_HUMAN	NQO1
(+)-Paeonilactone C	0.6811	Androgen receptor	ANDR_HUMAN	AR
(+)-Paeonilactone C	0.681	Glycogen phosphorylase, liver form	P06737	PYGL
(+)-Paeonilactone C	0.6804	Tyrosine-protein phosphatase non-receptor type 1	PTN1_HUMAN	PTPN1
(+)-Paeonilactone C	0.68	Peroxisome proliferator-activated receptor gamma	PPARG_HUMAN	PPARG
(+)-Paeonilactone C	0.6775	Amine oxidase [flavin-containing] B	AOFB_HUMAN	MAOB
(+)-Paeonilactone C	0.6758	Inosine-5-monophosphate dehydrogenase 2	IMDH2_HUMAN	IMPDH2
(+)-Paeonilactone C	0.6653	Progesterone receptor	PRGR_HUMAN	PGR
(+)-Paeonilactone C	0.6648	Chitotriosidase-1	Q13231	CHIT1
(+)-Paeonilactone C	0.6603	Cell division protein kinase 6	CDK6_HUMAN	CDK6
(+)-Paeonilactone C	0.6482	Serine/threonine-protein kinase PLK1	P53350	PLK1
(+)-Paeonilactone C	0.6428	Branched-chain-amino-acid aminotransferase, mitochondrial	O15382	BCAT2
(+)-Paeonilactone C	0.6377	Phenylalanine-4-hydroxylase	PH4H_HUMAN	PAH
(+)-Paeonilactone C	0.6364	Serine hydroxymethyltransferase, cytosolic	GLYC_HUMAN	SHMT1
(+)-Paeonilactone C	0.6329	Casein kinase I isoform gamma-2	KC1G2_HUMAN	CSNK1G2

(+)-Paeonilactone C	0.631	Ig lambda chain V-II region MGC	P01709	IGLV2-8
(+)-Paeonilactone C	0.6216	Phosphoenolpyruvate carboxykinase, cytosolic [GTP]	P35558	PCK1
(+)-Paeonilactone C	0.6216	Inositol monophosphatase	IMPA1_HUMAN	IMPA1
(+)-Paeonilactone C	0.6216	Annexin A5	ANXA5_HUMAN	ANXA5
(+)-Paeonilactone C	0.6112	Zinc-alpha-2-glycoprotein	ZA2G_HUMAN	AZGP1
(+)-Paeonilactone C	0.5992	Fibroblast growth factor receptor 2	P21802	FGFR2
(+)-Paeonilactone C	0.5971	cAMP-specific 3,5-cyclic phosphodiesterase 4D	PDE4D_HUMAN	PDE4D
(+)-Paeonilactone C	0.5951	Methionine aminopeptidase 2	AMPM2_HUMAN	METAP2
(+)-Paeonilactone C	0.5935	Ephrin type-B receptor 4	EPHB4_HUMAN	EPHB4
(+)-Paeonilactone C	0.5917	Pancreatic alpha-amylase	AMYP_HUMAN	AMY2A
(+)-Paeonilactone C	0.5913	Ribonuclease 4	P34096	RNASE4
(+)-Paeonilactone C	0.5906	Bile salt sulfotransferase	Q06520	SULT2A1
(+)-Paeonilactone C	0.5903	Dipeptidyl peptidase 4	DPP4_HUMAN	DPP4
(+)-Paeonilactone C	0.5898	Superoxide dismutase [Mn], mitochondrial	P04179	SOD2
(+)-Paeonilactone C	0.5895	Cytidine deaminase	CDD_HUMAN	CDA
(+)-Paeonilactone C	0.5891	Lanosterol synthase	ERG7_HUMAN	LSS
(+)-Paeonilactone C	0.589	Glycogen synthase kinase-3 beta	GSK3B_HUMAN	GSK3B
(+)-Paeonilactone C	0.5877	Tyrosine-protein kinase ITK/TSK	Q08881	ITK
(+)-Paeonilactone C	0.5871	Death-associated protein kinase 1	DAPK1_HUMAN	DAPK1
(+)-Paeonilactone C	0.5851	Tyrosine-protein kinase SYK	KSYK_HUMAN	SYK
(+)-Paeonilactone C	0.5847	cAMP-dependent protein kinase catalytic subunit alpha	P00517	PRKACA
(+)-Paeonilactone C	0.5847	Macrophage metalloelastase	MMP12_HUMAN	MMP12
(+)-Paeonilactone C	0.5843	Serine/threonine-protein kinase 6	O14965	AURKA
(+)-Paeonilactone C	0.5841	Poly [ADP-ribose] polymerase 1	P09874	PARP1
(+)-Paeonilactone C	0.5838	Serum albumin	ALBU_HUMAN	ALB
(+)-Paeonilactone C	0.5828	Cyclin-T1	CCNT1_HUMAN	CCNT1
(+)-Paeonilactone C	0.5822	Corticosteroid 11-beta-dehydrogenase isozyme 1	DHI1_HUMAN	HSD11B1
(+)-Paeonilactone C	0.5799	Phosphatidylinositol-4,5-bisphosphate 3-kinase catalytic subunit gamma isoform	PK3CG_HUMAN	PIK3CG
(+)-Paeonilactone C	0.5778	Eosinophil cationic protein	P12724	RNASE3
(+)-Paeonilactone C	0.5776	ADP-ribosyl cyclase 2	BST1_HUMAN	BST1
(+)-Paeonilactone C	0.5773	NAD-dependent malic enzyme, mitochondrial	MAOM_HUMAN	ME2
(+)-Paeonilactone C	0.5771	Matrilysin	MMP7_HUMAN	MMP7
(+)-Paeonilactone C	0.5765	3-hydroxy-3-methylglutaryl-coenzyme A reductase	HMDH_HUMAN	HMGCR
(+)-Paeonilactone C	0.5761	Uridine-cytidine kinase 2	Q9BZX2	UCK2
(+)-Paeonilactone C	0.5729	Sorbitol dehydrogenase	DHSO_HUMAN	SORD
(+)-Paeonilactone C	0.5727	Aldo-keto reductase family 1 member C1	Q04828	AKR1C1
(+)-Paeonilactone C	0.5708	Estradiol 17-beta-dehydrogenase 1	P14061	HSD17B1
(+)-Paeonilactone C	0.5686	Cathepsin G	CATG_HUMAN	CTSG
(+)-Paeonilactone C	0.5682	Dihydrofolate reductase	DYR_HUMAN	DHFR
(+)-Paeonilactone C	0.5675	Leukotriene A-4 hydrolase	LKHA4_HUMAN	LTA4H
(+)-Paeonilactone C	0.5673	Tyrosyl-tRNA synthetase, cytoplasmic	P54577	YARS1
(+)-Paeonilactone C	0.5672	Adenosylhomocysteinase	SAHH_HUMAN	AHCY

(+)-Paeonilactone C	0.5665	Histone deacetylase 8	HDAC8_HUMAN	HDAC8
(+)-Paeonilactone C	0.5617	Retinoic acid receptor RXR-alpha	RXRA_HUMAN	RXRA
(+)-Paeonilactone C	0.5606	Baculoviral IAP repeat-containing protein 4	XIAP_HUMAN	XIAP
(+)-Paeonilactone C	0.5601	Serine/threonine-protein kinase PAK 7	PAK7_HUMAN	PAK7
(+)-Paeonilactone C	0.5601	Hepatocyte growth factor receptor	P08581	MET
(+)-Paeonilactone C	0.5591	Ras-related C3 botulinum toxin substrate 2	P15153	RAC2
(+)-Paeonilactone C	0.5585	Caspase-3	CASP3_HUMAN	CASP3
(+)-Paeonilactone C	0.5577	Complement component C8 gamma chain	P07360	C8G
(+)-Paeonilactone C	0.5539	Aldo-keto reductase family 1 member C3	AK1C3_HUMAN	AKR1C3
(+)-Paeonilactone C	0.5535	Activated CDC42 kinase 1	ACK1_HUMAN	TNK2
(+)-Paeonilactone C	0.553	Copper transport protein ATOX1	O00244	ATOX1
(+)-Paeonilactone C	0.5515	Tissue-type plasminogen activator	TPA_HUMAN	PLAT
(+)-Paeonilactone C	0.5513	Aldehyde dehydrogenase, mitochondrial	ALDH2_HUMAN	ALDH2
(+)-Paeonilactone C	0.5506	Cytochrome P450 2C9	CP2C9_HUMAN	CYP2C9
(+)-Paeonilactone C	0.5456	Alcohol dehydrogenase class-3	ADHX_HUMAN	ADH5
(+)-Paeonilactone C	0.5424	C-C motif chemokine 5	CCL5_HUMAN	CCL5
(+)-Paeonilactone C	0.5422	Endoplasmic reticulum mannosyl-oligosaccharide 1,2-alpha-mannosidase	Q9UKM7	MAN1B1
(+)-Paeonilactone C	0.5318	Lithostathine-1-alpha	P05451	REG1A
(+)-Paeonilactone C	0.5281	Dihydroorotate dehydrogenase, mitochondrial	PYRD_HUMAN	DHODH
(+)-Paeonilactone C	0.5228	Renin	RENI_HUMAN	REN
(+)-Paeonilactone C	0.5196	Uridine 5-monophosphate synthase	P11172	UMPS
(+)-Paeonilactone C	0.513	Alcohol dehydrogenase 1C	P00326	ADH1C
(+)-Paeonilactone C	0.5111	Epoxide hydrolase 2	HYES_HUMAN	EPHX2
(+)-Paeonilactone C	0.5053	Heat shock protein homolog SSE1	P32589	SSE1
(+)-Paeonilactone C	0.5	Bifunctional 3-phosphoadenosine 5-phosphosulfate synthetase 1	O43252	PAPSS1
