# Supplementary material for: Integrating UPLC-Q-TOF-MS and Network Pharmacology to Explore the Potential Mechanisms of Paeonia lactiflora Pall. in the Treatment of Blood Stasis Syndrome
Source: Molecules. 2024 Jun 26;29(13):3019. doi: 10.3390/molecules29133019 (PMC11243510; doi:10.3390/molecules29133019)
Supplement: Supplementary file 1 [file molecules-29-03019-s001.zip › Table S5 The disease targets obtained from Genecards and OMIM databases.pdf]

Table S5 The disease targets obtained from Genecards and OMIM databases

Genecards Gene Symbol		OMIM Gene Symbol		Intersection
blood stasis	blood stasis syndrome	blood stasis	blood stasis syndrome	
ABCB11	ABCB11	A3GALT2	A3GALT2	AKR1B1
ABCB4	ABCB4	ACADM	ACAP4	NOS3
ABCC1	ABCC1	ADORA3	ACF7	F2
ABCC2	ABCC2	AGL	ADPRHL2	ACE
ABCC3	ABCC3	AHCYL1	AFAR2	MAPK14
ABCC4	ABCC4	AK2	AGGRUS	PLAU
ABCC8	ABCC8	AKR7A3	AGRN	HSP90AA1
ABCC9	ABCC9	AMY2A	AHDC1	MMP7
ABCG5	ABCG5	ANGPTL3	AITR	NR3C2
ABCG8	ABCG8	ANGPTL7	AK2	EPHB4
ACE	ACE	ARID1A	AKR7A3	SRC
ACE2	ACE2	ARTN	ALGAZ	CDK6
ACOX2	ACOX2	ASAP3	ALPL	TTR
ACVRL1	ACVRL1	ATAD3A	ALS10	PGR
ADAM17	ADAM17	ATP13A2	ANGPTL7	PPARG
ADAMTS13	ADAMTS13	ATP1A1	ANP	EGFR
ADAMTSL1	ADAMTSL1	ATP1F1	AOF2	ESR1
ADGRG2	ADGRG2	B6QTL1	AOMS4	TGFBR1
ADIPOQ	ADIPOQ	BCL10	APDS	KDR
ADM	ADM	BCL9	APITD1	PPARD
ADORA1	ADORA1	C1orf86	APNH	FGFR1
ADORA2B	ADORA2B	C8A	APP1	IGF1R
ADRB1	ADRB1	CAMK2N1	ARCC2	NR1H2
ADRB2	ADRB2	CASZ1	ARH3	MMP13
AGT	AGT	CD101	ARID1A	RXRA
AGTR1	AGTR1	CD160	ARTN	PIK3CG

AKAP9	AKAP9	CD2	ASAP3	AR
AKR1B1	AKR1B1	CDA	ASML3B	F10
AKT1	AKT1	CDC14A	ASP3	ALB
ALB	ALB	CDC42	ATAD3A	MDM2
ALOX12	ALOX12	CDK11A	ATFB6	PDE5A
ALOX5	ALOX5	CDK11B	ATP13A2	REN
ALPP	ALPP	CELA2A	ATP1F1	ESR2
AMPD1	AMPD1	CHD5	ATRST2	BMP7
ANGPT1	ANGPT1	CLCNKB	B120	MET
ANGPT2	ANGPT2	CLIC4	B3GALT6	F7
ANGPT4	ANGPT4	CMM	B6QTL1	PLK1
ANGPTL1	ANGPTL1	COA7	BCC1	TYMS
ANK2	ANK2	CPT2	BDPLT22	AKT1
ANO6	ANO6	CSF1	BHC110	NR1I2
AOC3	AOC3	CSF3R	BNAH2	IL2
APC	APC	CTH	BNP	ADAM17
APLN	APLN	CTPS1	C1DELp35	ERBB4
APLNR	APLNR	CTRCT8	C1DELp36	LGALS3
APOB	APOB	CYP4A11	C1DUPp36.33	FABP3
APOE	APOE	CYP4X1	C1orf4	MMP2
APOH	APOH	DAB1	C1orf86	PLAT
AQP2	AQP2	DBT	C1QA	MMP9
AR	AR	DDAH1	C1QB	NR1I3
ATP8B1	ATP8B1	DHCR24	C1QC	ACE2
AURKA	AURKA	DHDDS	C1QG	NR1H4
BAX	BAX	DIO1	CAMK2N1	AURKA
BCL2	BCL2	DPYD	CAMTA1	SERPINA1
BCL2L1	BCL2L1	E2F2	CAPB	ITGAL
BDKRB1	BDKRB1	ECE1	CASZ1	RBP4
BLVRA	BLVRA	EDN2	CB2	MME

BMP7	BMP7	ENO1	CCDC28B	TPH1
BPIFA1	BPIFA1	EPB41	CCV	NOS2
BRAF	BRAF	EPHA2	CD137	HMOX1
CALCA	CALCA	EPHB2	CDA	TEK
CAV3	CAV3	ERMAP	CDC2L1	KIT
CCK	CCK	ERVK-7	CDC2L2	
CCKAR	CCKAR	ESPN	CDC42	
CCL2	CCL2	F3	CDK11	
CCM2	CCM2	FAF1	CDK11A	
CCR7	CCR7	FGR	CDK11B	
CD36	CD36	FNDC5	CDT6	
CD40LG	CD40LG	FUCA1	CECBA	
CD44	CD44	FYB2	CELA2A	
CD63	CD63	GALE	CENPS	
CD79A	CD79A	GBP5	CENTB6	
CDC25C	CDC25C	GCLM	CEP104	
CDC7	CDC7	GFI1	CFAP57	
CDH5	CDH5	GJA4	CFTD	
CDK1	CDK1	GJA5	CHC1	
CDK4	CDK4	GJA8	CHD5	
CDK6	CDK6	GLMN	CLCNKA	
CDKL5	CDKL5	GNB1	CLCNKB	
CDKN2A	CDKN2A	GSTM1	CLCR	
CDKN2B-AS1	CDKN2B-AS1	GSTM2	CLDN19	
CDKN3	CDKN3	GUCA2A	CLIC4	
CEBPA	CEBPA	GUCA2B	CLN1	
CFTR	CFTR	H6PD	CMD1LL	
CLCA1	CLCA1	HJV	CMM	
CNTF	CNTF	HMGCS2	CMS8	
COL3A1	COL3A1	HSD3B1	CMT2A	

COL4A1	COL4A1	ID3	CMT2A1
CP	CP	IL22RA1	CNK
CPSF3	CPSF3	IL23R	CNR2
CRP	CRP	ITGB3BP	COL9A2
CSF2	CSF2	JAK1	CONDSIAS
CSN1S1	CSN1S1	KCNA3	CORTRD1
CXCL12	CXCL12	KIF1B	CPRF
CXCL8	CXCL8	KIF2C	CSF3R
CYP11B2	CYP11B2	LAPTM5	CSS2
CYP1B1	CYP1B1	LCK	CTPA
CYP27A1	CYP27A1	LEPR	CTPP1
CYP2A6	CYP2A6	LRIF1	CTPS
CYP2C19	CYP2C19	LRRC8B	CTPS1
CYP2F1	CYP3A4	LRRC8C	CTRC
CYP3A4	CYP7A1	LRRC8D	CTRCT49
CYP7A1	CYP8B1	MASP2	CTRCT6
CYP8B1	DBH	MDS2	CTRCT8
DBH	DEFB104A	MFSD2A	CX31
DEFB104A	DEFB104B	MIB2	CX37
DEFB104B	DEFB105A	MIR34A	CX5
DEFB105A	DEFB105B	MMACHC	CYP4A11
DEFB105B	DEPDC5	MPL	CYP4X1
DEPDC5	DLX6-AS1	MTHFR	CYPIVX1
DLX6-AS1	DNMT1	MTOR	DBA7
DNMT1	EDN1	MUTYH	DDEFL1
EDN1	EDNRA	MXRA8	DEDSM
EDNRA	EGF	MYSM1	DEDSSH2
EGF	EGFR	NMNAT1	DEE15
EGFR	EGR1	NPPA	DEE18
EGR1	EIF4EBP1	NPPB	DEL1p35

EIF4EBP1	EPHB4	NRAS	DEL1p36
EPHB4	EPO	OFC13	DFNA2B
EPO	ERBB2	OLFML3	DFNB96
ERBB2	ERBB4	OPRD1	DHDDS
ERBB4	ESR1	P3H1	DNB5
ESR1	ESR2	PABPC4	DNS
ESR2	EZR	PADI1	DPH2
EZR	F10	PADI6	DPH2L2
F10	F2	PALMD	DR3
F2	F3	PCSK9	DRS2
F3	F5	PDE4DIP	DRT
F5	F7	PDPN	DSCR1L2
F7	F8	PGD	DUP1p36.33
F8	F9	PGM1	DVL1
F8A1	FABP3	PHGDH	DYT18
F9	FCGRT	PIGK	DYT9
FABP3	FGA	PIK3R3	DYTOABG
FCGRT	FGF10	PINK1	E2F2
FGA	FGF19	PLA2G2A	EAP
FGF10	FGF2	PLK3	ECE1
FGF19	FGFR1	PLOD1	ECK
FGF2	FGFR4	POLR3C	EDM2
FGFR1	FLG	POLR3GL	EDN2
FGFR4	FLT1	POROK5	EDSKCL1
FLG	FLT4	PPT1	EDSSPD2
FLT1	FN1	PRDM16	EIF2B3
FLT4	FRMD4A	PRDX1	EIG10
FN1	FUT2	PRKAA2	EIG12
FRMD4A	FUT3	PRKCZ	EJM7
FUT2	G6PC3	PTCH2	EKVP1

FUT3	GAS5	PTPN22	EL1
G6PC3	GAS6	RAD54L	ELA2A
GAS5	GATA4	RBM15	ELOVL1
GAS6	GATA6	RBM8A	ENO1
GATA4	GFER	RCAN3	EPB41
GATA6	GGT1	RHCE	EPHA2
GFER	GH1	RHD	EPHB2
GGT1	GJA1	RPL11	EPHT3
GH1	GJA5	RPL22	ERK
GJA1	GLMN	RPL5	ERMAP
GJA5	GLP2R	S1PR1	ESPN
GLMN	GLUD1	SCZD12	EXOSC10
GLP2R	GNA11	SDHB	EYA3
GLUD1	GNA14	SEC22B	FAAP20
GNA11	GNA15	SKI	FACE1
GNA14	GNAQ	SLC16A1	FASPS3
GNA15	GNB2	SLC22A15	FCN3
GNAQ	GP1BA	SLC25A33	FGR
GNB2	GP6	SLC2A1	FLJ40906
GP1BA	GPT	SLC45A1	FNDC5
GP6	GSN	SLC6A9	FRAP1
GPT	HBA2	SMIM1	FRCP2
GSN	HCRT	SMPDL3B	FUCA1
HBA2	HDAC3	SNIP1	FUSIP1
HCRT	HFE	SORT1	FWS
HDAC3	HIF1A	SRSF10	G6PDH
HFE	HLA-DPB1	ST6GALNAC5	GABRD
HIF1A	HLA-DQB1	STIL	GALE
HLA-DPB1	HLA-DRB1	STX12	GAMOS10
HLA-DQB1	HMGB1	STXBP3	GBD2

HLA-DRB1	HMOX1	SYTL1	GCENSG
HMGB1	HNF1B	TAL1	GCSFR
HMOX1	HNF4A	TIE	GDH
HNF1B	HOTAIR	TMEM50A	GEFSP5
HNF4A	HP	TMEM59	GITR
HOTAIR	HRH1	TNFRSF18	GJA4
HP	HRH2	TNFRSF1B	GJB3
HRH1	HSD3B7	TNFRSF25	GLUT1
HRH2	HSP90AA1	TNFRSF9	GLUT1DS
HSD3B7	HTR3A	TRIM33	GLYBP
HSP90AA1	HTR4	TSHB	GLYT1
HTR3A	ICAM1	TXNIP	GNB1
HTR4	IFNG	UBIAD1	GP36
ICAM1	IGF1R	UROD	GRHL3
IFNG	IL10	UTS2	GROS1
IGF1R	IL10RA	VAMP3	GUCA2
IL10	IL18	VAV3	GUCA2A
IL10RA	IL1B	VCAM1	GUCA2B
IL18	IL2	VCTN1	H6PD
IL1B	IL6	WASF2	HAKA1
IL2	IL9	WLS	HAYOS
IL6	INS	ZC3H12A	HDFNRH
IL9	ITGA2	ZFP69	HDS
INS	ITGA2B	ZMPSTE24	HIAA0929
ITGA2	ITGA4	2ADUB	HLD23
ITGA2B	ITGAL	AFAR2	HMGCL
ITGA4	ITGAM	AIIDE	HOMG5
ITGAL	ITGAX	AITR	HOPS
ITGAM	ITGB2	ALPS4	HPDL
ITGAX	ITGB3	AMPK	HPMRS1

ITGB2	JAG1	ANGPT5	HPPA
ITGB3	KCNA5	ARAP	HPPC
JAG1	KCNE1	ASML3B	HPPI
KCNA5	KCNE2	ASP3	HPPO
KCNE1	KCNJ2	B7H4	HR54
KCNE2	KCNJ5	BCATE2	HRAD54
KCNJ2	KCNQ1	BNP	HSPG2
KCNJ5	KDR	BY55	HTLVR
KCNQ1	KIF12	C1orf4	ICMT
KDR	KIT	CCV	ID3
KIF12	KLB	CDC2L1	IDDNPF
KIT	KLF11	CDC2L2	IF1
KLB	KNG1	CDT6	IGB3S
KLF11	KRAS	CHNG4	IKSHD
KLHDC8A	LALBA	CLN1	IL22R1
KNG1	LAP3	CMT2A	IL22RA1
KRAS	LCTL	CMT2DD	ILA
LALBA	LDLR	CNK	IMD14A
LAP3	LEP	CTPS	IMD14B
LCTL	LEPQTL1	CX37	IMD22
LDLR	LGALS1	CX40	IMD24
LEP	LGALS3	CX50	IPABP
LEPQTL1	LIF	CYPIVX1	IRIP
LGALS1	LMNA	DBA6	JBTS25
LGALS3	LOC110121269	DBA7	JFC1
LIF	LPA	DCAL	JTK14
LMNA	LPO	DCF1	KDM1A
LOC110121269	LTF	DFNB32	KHDRBS1
LOC122128420	MALT1	DNB5	KIAA0467
LPA	MAPK14	DPD	KIAA0562

LPO	MBL2	DSAP3	KIAA0601
LTF	MBP	DSCR1L2	KIAA0833
MALT1	MCM2	EAP	KIAA1251
MAPK14	MDM2	ECK	KIAA1693
MBL2	MET	EDG1	KIF1B
MBP	MFN2	EL1	KIF2C
MCM2	MLH1	ELA2A	KNSL6
MDM2	MLN	EPHT3	KONDS
MET	MME	FAAP20	KRPPD
MFN2	MMP1	FACE1	LAPTM5
MLH1	MMP13	FAD158	LAR
MLN	MMP2	FLIPT1	LARD
MME	MMP7	FRAP1	LCA9
MMP1	MMP9	FRCP2	LCK
MMP13	MPO	FUSIP1	LEPRE1
MMP2	MPZ	GCSFR	LGMDR15
MMP7	MTHFR	GDE	LH1
MMP9	MTOR	GDH	LIKNS
MPO	MUC2	GLCLR	LIN28A
MPZ	MUC5AC	GLUT1	LIS9
MTHFR	MYBPC3	GLYT1	LLH
MTOR	MYC	GPI8	LMPHM11
MUC2	MYH6	GPR177	LSD1
MUC5AC	MYH7	GSD14	LVNC8
MYBPC3	MYL4	GST4	MACF1
MYC	MYLK	GUCA2	MACST
MYH6	MYLK3	GVM	MADB
MYH7	MYO5B	HAYOS	MASP2
MYL4	NAA10	HDFNRH	MC2DN4
MYLK	NAGLU	HDS	MCAK

MYLK3	NANOG	HERV-KIII	MCIP3
MYO5B	NF1	HFAF1	MCPH7
NAA10	NF2	HFE2A	MCPIP
NAGLU	NFKBIA	HR54	MCPIP1
NANOG	NME4	IBD17	MDDGA3
NF1	NODAL	IF1	MDDGB3
NF2	NOS1	IGB3S	MDDGC3
NFKBIA	NOS2	IGSF2	MDS
NME4	NOS3	IIAE4	MDS2
NODAL	NOTCH2	IL22R1	MEB
NOS1	NPPA	ILA	MECR
NOS2	NPPA-AS1	IMD22	MEL1
NOS3	NPPB	IMD37	MFSD2A
NOTCH2	NPPC	JTK14	MGC1203
NPPA	NPR1	KIAA0018	MHF1
NPPA-AS1	NPY	KNSL6	MIB2
NPPB	NR0B2	LEPRE1	MINT
NPPC	NR1H2	LH1	MIR34A
NPR1	NR1H4	LRRC5	MIRN34A
NPY	NR1I2	MCAD	MIZ1
NR0B2	NR1I3	MCPIP	MLM
NR1H2	NR3C2	MCSF	MMACHC
NR1H4	NUP155	MCT1	MOM1
NR1I2	ODC1	MEL1	MPB1
NR1I3	OSM	MIRN34A	MPL
NR3C2	PAFAH1B2	MLM	MPLV
NUP155	PCNA	MMGL	MRD14
ODC1	PDE5A	MRD42	MRD25
OSM	PEBP1	MTCLIC	MRD42
PAFAH1B2	PECAM1	MUNC18C	MRT12

PCNA	PF4	MYH	MRT77
PDE5A	PGR	NARC1	MTCLIC
PEBP1	PIK3CA	NEDHCS	MTHFR
PECAM1	PIK3CG	NEDMISBA	MTOR
PF4	PLAT	NLS1	MUTYH
PGR	PLAU	NMNAT	MXRA8
PIK3CA	PLAUR	NRIF3	MYH
PIK3CG	PLK1	NT3	MYOSCO
PLAT	PLN	OBR	NALD
PLAU	PLP1	OLF44	NBLST1
PLAUR	PPARD	PABP4	NBPF1
PLK1	PPARG	PALML	NEDBEH
PLN	PPARGC1A	PARK6	NEDHCS
PLP1	PPAT	PARK9	NEDMISBA
PPARD	PPBP	PEP	NEDSWMA
PPARG	PPT1	PKC2	NHE1
PPARGC1A	PRIM1	PLA2B	NKEFA
PPAT	PROC	PMNDS	NMNAT
PPBP	PRSS8	PND	NMNAT1
PPT1	PTAFR	PPH	NPHP4
PRIM1	PTEN	PREMBL2	NPPA
PROC	PTGS1	PRXI	NPPB
PRSS8	PTGS2	RBM8B	NR0B2
PTAFR	PYCARD	RESA1	NRBF1
PTEN	PYY	RHNA	OFC13
PTGS1	RAF1	RIF1	OI8
PTGS2	RASA1	RPC62	OPRD1
PYCARD	RASL12	SC	OTS8
PYY	RB1	SCA37	P3H1
RAF1	RBP4	SDH2	P58

RASA1	REN	SEC22L1	PABP4
RASL12	RHOB	SGS	PABPC4
RB1	RICTOR	SIAT7E	PADI1
RBP4	RPTOR	SIL	PADI3
REN	RRAD	SLP1	PADI6
RHOB	RUNX1	SMP1	PAGA
RICTOR	RUNX1T1	SOFM	PANK4
RPTOR	RXRA	SPEN	PARK6
RRAD	SCN1A	SRC2	PARK9
RUNX1	SCN1B	SRG	PAX7
RUNX1T1	SCN2B	STX13	PBD13A
RXRA	SCN3B	TALRRP	PBD6A
SCN1A	SCN4B	TCL5	PBD6B
SCN1B	SCN5A	TERE1	PCBC
SCN2B	SCNN1A	TFA	PCH7
SCN3B	SCNN1B	TI1A	PDPN
SCN4B	SCNN1G	TIF1G	PED
SCN5A	SDHB	TKS	PER3
SCNN1A	SELE	TNFR2	PEX10
SCNN1B	SELL	TNFRSF12	PEX14
SCNN1G	SELP	TPOR	PGD
SDHB	SELPLG	TXDI1	PGL4
SELE	SERPINA1	UGN	PHRINL
SELL	SERPINA3	UPLC1	PIGV
SELP	SERPINC1	USH1M	PIK3CD
SELPLG	SERPINE1	VDUP1	PIK3R3
SEMG1	SERPINF1	VEL	PINK1
SEMG2	SERPINF2	WAVE2	PKC2
SERPINA1	SLC10A1	ZNF163	PLA2B
SERPINA3	SLC10A2	ZNF642	PLA2G2A

SERPINC1	SLC17A5	AD158	PLA2L
SERPINE1	SLC22A1	ANP	PLC
SERPINF1	SLC22A2	AOMS4	PLK3
SERPINF2	SLC25A13	ATFB11	PLOD1
SLC10A1	SLC4A2	B120	PMNDS
SLC10A2	SLC51A	B7X	PMSCL2
SLC17A5	SLC51B	C1orf168	PNAT1
SLC22A1	SLC6A2	CD137	PND
SLC22A2	SLCO1A2	CDG1T	POMGNT1
SLC25A13	SLCO1B1	CENTB6	PPH
SLC4A2	SMPD2	CMT2A1	PPT1
SLC51A	SMPD3	CTPP1	PRDM16
SLC51B	SNTA1	CTRCT1	PRDX1
SLC6A2	SOAT1	DNS	PREMBL2
SLCO1A2	SOCS3	DR3	PRK
SLCO1B1	SOD1	DRT	PRKCZ
SMPD2	SRC	EVI	PRXI
SMPD3	SRY	FHBL2	PTCH2
SNTA1	SST	FSHD3	PTOS1
SOAT1	STC1	G6PDH	PTPRF
SOCS3	SYNE1	GCENSG	RAD54L
SOD1	SYNGAP1	GITR	RATARS
SRC	TBX5	GROS1	RCAN3
SRY	TBXAS1	HCHOLA3	RCC1
SST	TEK	HERV-K102	RD
STC1	TERT	HHF7	RERE
SYNE1	TF	HOMGSMR2	RHCE
SYNGAP1	TFAM	HRAD54	RHD
TBX5	TGFB1	HTLVR	RHNA
TBXAS1	TGFBR1	IDDNPF	RMS2

TEK	TGM4	IMD24	RNF220
TERT	THBD	IPABP	ROCHIS
TF	THBS1	IRBIT	RP59
TFAM	TIAM1	JFC1	RP76
TGFB1	TIE1	KIAA0231	RPL11
TGFBR1	TIMP1	KIAA0454	RPL22
TGM4	TJP2	KIAA1915	RPS6KA1
THBD	TKT	KRPPD	RSDM1
THBS1	TNC	LDLCQ6	RSK1
TIAM1	TNF	LEPRD	RSMD1
TIE1	TNNI3	LLH	RSPO1
TIMP1	TNNT2	LVNC8	SAM68
TJP2	TNXA	MCAK	SC
TKT	TP53	MCIP3	SCAR2
TNC	TP63	MCPH7	SCAR4
TNF	TPH1	MCPIP1	SCCD
TNNI3	TRIM37	MDS	SCL
TNNT2	TSC2	MPB1	SCN7
TNXA	TTN	MPLV	SCNN1D
TP53	TTR	NEDHCAS	SCZD12
TP63	TULP1	NS6	SDC3
TPH1	TUSC7	OTT	SDCHCN
TRIM37	TYMS	P58	SDCN
TSC2	UCP1	PAGA	SDH2
TTN	UNC45A	PHGDHD	SDHB
TTR	USP53	PHRINL	SDHIP
TULP1	UTP4	PLA2L	SELENON
TUSC7	UTS2	PNAT1	SELN
TYMS	VCAM1	PRK	SEMDJL1
UCP1	VCL	PSP	SEPN1

UNC45A
USP53
UTP4
UTS2
VCAM1
VCL
VEGFA
VIPAS39
VPS33B
VPS50
VTN
VWF
XDH
ZC4H2
ZNF667-AS1

VEGFA
VIPAS39
VPS33B
VPS50
VTN
VWF
XDH
ZC4H2
ZNF667-AS1

PTPN8
RD
RFG7
RP59
RPC3
S1P1
SCAR2
SCCD
SCL
SCN2
SCN7
SDHIP
SELRC1
SKS
STE24
T1A2
TAR
TASR
THMA2
TIE1
TNFBR
VMGLOM
APP1
ATFB6
B7S1
BMFS4
C1DELq21.1
C1orf163
CDK11
CMD1LL

SERKAL
SGS
SHARP
SHILCA
SHP
SIAT6
SIL
SJA
SJS
SJS1
SKI
SKS
SLC25A33
SLC2A1
SLC45A1
SLC6A9
SLC9A1
SLP1
SLSN4
SMARCF1
SMIM1
SMP1
SMPDL3B
SNIP1
SOM
SPEN
SPG78
SPG83
SRC2
SRG

CMNS	SRSF10
CORTRD1	SSC1
CTPA	ST3GAL3
CZP1	ST3GALII
DDEFL1	STE24
DEDSM	STIL
DYT18	STL5
EDSKCL1	STX12
ERK	STX13
FH3	SYND3
GP36	SYTL1
KIAA0477	SZT2
LARD	T1A2
LCA9	TAL1
LMPHM11	TARDBP
LYP	TASR
MADB	TASR1
MCT1D	TASR2
MOM1	TCL5
NBLST1	TDP43
NKEFA	TEKT2
OI8	TEKTB1
PGL4	TERE1
PTC7	TFCP2L4
SMARCF1	THCYT2
SPG78	TI1A
TASR1	TIE
THCYT2	TIE1
ZKS	TKS
ACAP4	TMEM50A

ARCC2
ATRST2
CAE1
DEL1q21.1
LDLCQ1
MC2DN4
MRD14
NCMS
OTS8
PCBC
PED
RSDM1
SCAN3
SHILCA
TASR2
AGGRUS
CAPB
CSS2
CTRCT6
FHCL3
GLUT1DS
BDPLT22
EIG12
DYT9
SDCHCN

TNFBR
TNFR2
TNFRSF12
TNFRSF18
TNFRSF1B
TNFRSF25
TNFRSF9
TNSALP
TOE1
TPOR
UBIAD1
UGN
UHS1
UPLC1
UROD
USH1M
UTS2
VAMP3
VEL
VPS13D
VWS2
WASF2
WAVE2
WDR65
WNT4
XIGIS
YRDC
ZBTB17
ZC3H12A
ZFP69

		ZMPSTE24<br>ZNF151<br>ZNF642
