# Supplementary material for: Integrating UPLC-Q-TOF-MS and Network Pharmacology to Explore the Potential Mechanisms of Paeonia lactiflora Pall. in the Treatment of Blood Stasis Syndrome
Source: Molecules. 2024 Jun 26;29(13):3019. doi: 10.3390/molecules29133019 (PMC11243510; doi:10.3390/molecules29133019)
Supplement: Supplementary file 1 [file molecules-29-03019-s001.zip › Table S6 The functional and pathway enrichment analysis results.pdf]

Table S6 The functional and pathway enrichment analysis results

BPs	MPs	CCs	KEGG
positive regulation of kinase activity	RNA polymerase II transcription factor activity, ligand-activated sequence-specific DNA binding	receptor complex	Pathways in cancer
negative regulation of apoptotic process	zinc ion binding	extracellular region	Proteoglycans in cancer
positive regulation of protein kinase B signaling	transmembrane receptor protein tyrosine kinase activity	extracellular space	PI3K-Akt signaling pathway
signal transduction	protein tyrosine kinase activity	cell surface	Prostate cancer
positive regulation of phosphatidylinositol 3-kinase signaling	sequence-specific DNA binding	membrane raft	Endocrine resistance
protein autophosphorylation	steroid hormone receptor activity	basal plasma membrane	Relaxin signaling pathway
transmembrane receptor protein tyrosine kinase signaling pathway	endopeptidase activity	plasma membrane	Estrogen signaling pathway
positive regulation of cell migration	enzyme binding	extracellular exosome	Fluid shear stress and atherosclerosis
response to estrogen	metallopeptidase activity	chromatin	Rap1 signaling pathway
proteolysis	protein binding	nucleus	Breast cancer
peptidyl-tyrosine phosphorylation	identical protein binding	nucleoplasm	Melanoma
multicellular organism development	serine-type endopeptidase activity	endoplasmic reticulum lumen	Chemical carcinogenesis - receptor activation
intracellular steroid hormone receptor signaling pathway	transcription factor activity, sequence-specific DNA binding	cytosol	MAPK signaling pathway
positive regulation of transcription, DNA-templated	metalloendopeptidase activity	macromolecular complex	Progesterone-mediated oocyte maturation
positive regulation of gene expression	steroid binding	caveola	HIF-1 signaling pathway

positive regulation of transcription from RNA polymerase II promoter	nitric-oxide synthase regulator activity	cytoplasm	Adherens junction
response to hypoxia	protein kinase activity	ficolin-1-rich granule lumen	Pathways in cancer
positive regulation of cell proliferation	ATP binding	perinuclear region of cytoplasm	Proteoglycans in cancer
positive regulation of protein phosphorylation	ATPase binding	focal adhesion	PI3K-Akt signaling pathway
positive regulation of smooth muscle cell proliferation	protein phosphatase binding		Prostate cancer
angiotensin maturation	transcription cofactor binding		Endocrine resistance
intracellular receptor signaling pathway	receptor binding		Relaxin signaling pathway
negative regulation of smooth muscle cell proliferation	transcription coactivator binding		Estrogen signaling pathway
negative regulation of gene expression	RNA polymerase II core promoter proximal region sequence-specific DNA binding		Fluid shear stress and atherosclerosis
positive regulation of MAP kinase activity	retinoid X receptor binding		Rap1 signaling pathway
cellular response to hypoxia	integrin binding		Breast cancer
positive regulation of apoptotic process	protein homodimerization activity		Melanoma
blood coagulation	RNA polymerase II transcription factor activity, sequence-specific DNA binding		Chemical carcinogenesis - receptor activation
cellular response to estradiol stimulus	transcriptional activator activity, RNA polymerase II transcription regulatory region sequence-specific binding		MAPK signaling pathway
positive regulation of cell growth	peptidase activity		Progesterone-mediated oocyte maturation

positive regulation of MAPK cascade	growth factor binding	HIF-1 signaling pathway
angiogenesis	fatty acid binding	Adherens junction
epidermal growth factor receptor signaling pathway	transcription regulatory region sequence-specific DNA binding	Bladder cancer
positive regulation of blood vessel endothelial cell migration	protein serine/threonine kinase activity	EGFR tyrosine kinase inhibitor resistance
mammary gland alveolus development	estrogen receptor binding	FoxO signaling pathway
male gonad development	estrogen receptor activity	Complement and coagulation cascades
aging	epidermal growth factor-activated receptor activity	Transcriptional misregulation in cancer
negative regulation of transcription from RNA polymerase II promoter	peptidyl-dipeptidase activity	Diabetic cardiomyopathy
peptidyl-tyrosine autophosphorylation	nitric-oxide synthase activity	Renin-angiotensin system
negative regulation of proteolysis	ligand-dependent nuclear receptor binding	Lipid and atherosclerosis
heart development		Chagas disease
circadian rhythm		VEGF signaling pathway
response to xenobiotic stimulus		Hepatocellular carcinoma
regulation of blood pressure		Ras signaling pathway
positive regulation of phosphatidylinositol 3-kinase activity		Epithelial cell signaling in Helicobacter pylori infection
positive regulation of protein localization to nucleus		Central carbon metabolism in cancer
negative regulation of extrinsic apoptotic signaling pathway		Prolactin signaling pathway
positive regulation of JAK-STAT cascade		Non-small cell lung cancer

cellular response to reactive oxygen species	Platelet activation
cell differentiation	Glioma
peptidyl-serine phosphorylation	Oocyte meiosis
cellular response to cadmium ion	Apelin signaling pathway
collagen catabolic process	AGE-RAGE signaling pathway in diabetic complications
response to drug	Coronavirus disease - COVID-19
liver regeneration	C-type lectin receptor signaling pathway
ovulation from ovarian follicle	Calcium signaling pathway
epithelial cell maturation	Th17 cell differentiation
phosphatidylinositol 3-kinase signaling	Thyroid hormone signaling pathway
extracellular matrix disassembly	Focal adhesion
positive regulation of phospholipase C activity	PPAR signaling pathway
epithelial cell proliferation	Pancreatic cancer
positive regulation of sequence-specific DNA binding transcription factor activity	Chronic myeloid leukemia
cellular response to UV-A	Chemical carcinogenesis - reactive oxygen species
negative regulation of cholesterol storage	Phospholipase D signaling pathway

ephrin receptor signaling pathway	Human cytomegalovirus infection
hormone-mediated signaling pathway	Gastric cancer
positive regulation of cyclin-dependent protein serine/threonine kinase activity	MicroRNAs in cancer
positive regulation of vascular smooth muscle cell proliferation	ErbB signaling pathway
response to nutrient levels	Cellular senescence
negative regulation of inflammatory response	Hepatitis B
positive regulation of fat cell differentiation	Small cell lung cancer
negative regulation of fibrinolysis	GnRH signaling pathway
beta-amyloid metabolic process	IL-17 signaling pathway
response to beta-amyloid	Parathyroid hormone synthesis, secretion and action
positive regulation of endothelial cell migration	Toxoplasmosis
insulin-like growth factor receptor signaling pathway	Leukocyte transendothelial migration
uterus development	Kaposi sarcoma-associated herpesvirus infection
retinoic acid receptor signaling pathway	Epstein-Barr virus infection
response to lipopolysaccharide	Osteoclast differentiation
cellular response to estrogen stimulus	Regulation of actin cytoskeleton

fibrinolysis	Human T-cell leukemia virus 1 infection
response to vitamin A	Yersinia infection
positive regulation of endothelial cell proliferation	Signaling pathways regulating pluripotency of stem cells
steroid hormone mediated signaling pathway	Acute myeloid leukemia
cell migration involved in sprouting angiogenesis	Oxytocin signaling pathway
positive regulation of proteasomal ubiquitin-dependent protein catabolic process	Shigellosis
intracellular estrogen receptor signaling pathway	Non-alcoholic fatty liver disease
nitric oxide biosynthetic process	Hepatitis C
cellular response to mechanical stimulus	Endocytosis
maternal process involved in female pregnancy	
response to immobilization stress	
lung development	
endothelial cell proliferation	
regulation of protein binding	
cellular response to lipopolysaccharide	
positive regulation of cholesterol efflux	
negative regulation of intrinsic apoptotic signaling pathway	
regulation of blood vessel diameter	
vascular endothelial growth factor receptor signaling pathway	

protein
phosphorylation
cellular response to
vascular endothelial
growth factor stimulus
positive regulation of
reactive oxygen
species metabolic
process
glucose homeostasis
positive regulation of
ERK1 and ERK2
cascade
cell-cell signaling
cellular response to
dexamethasone
stimulus
kidney development
smooth muscle
hyperplasia
positive regulation of
vasoconstriction
blood vessel
remodeling
lipopolysaccharide-
mediated signaling
pathway
placenta development
response to nicotine
lung alveolus
development
transcription from
RNA polymerase II
promoter
vasodilation
embryo implantation
positive regulation of
protein tyrosine kinase
activity
stem cell
differentiation
ovarian follicle
development
positive regulation of
Notch signaling
pathway
animal organ
regeneration
skeletal system
development

response to ethanol
inflammatory response
response to UV-A
tertiary branching involved in mammary gland duct morphogenesis
substance P catabolic process
epithelial to mesenchymal transition
negative regulation of receptor activity
response to cAMP
cell migration
cellular response to epidermal growth factor stimulus
phosphatidylinositol-mediated signaling
negative regulation of transcription, DNA-templated
