# Supplementary material for: Integrating UPLC-Q-TOF-MS and Network Pharmacology to Explore the Potential Mechanisms of Paeonia lactiflora Pall. in the Treatment of Blood Stasis Syndrome
Source: Molecules. 2024 Jun 26;29(13):3019. doi: 10.3390/molecules29133019 (PMC11243510; doi:10.3390/molecules29133019)
Supplement: Supplementary file 1 [file molecules-29-03019-s001.zip › TableS1-S3.pdf]

Table S1 Bleeding and clotting times on day 13 and day 23($\bar{x} \pm s$, n=6)

	Day 13		Day 23	
	Bleeding time(s)	Clotting time(s)	Bleeding time(s)	Clotting time(s)
Control	103.00±9.89	213.30±12.29	113.30±6.67	210.00±3.65
Model	53.33±3.33**	86.67±9.55**	40.00±4.47**	80.00±5.16**
Aspirin	51.67±4.77**	93.33±4.94**	66.67±4.22##	138.30±6.01##
FDT	51.67±4.77**	93.33±4.94**	73.33±4.22##	146.70±4.94##
PRA	60.00±5.77**	101.70±4.77**	81.67±4.77##	165.00±4.28##
PRR	56.67±4.22**	86.67±4.27**	71.67±4.77##	148.30±4.77##

Note: compared to the control group, **P<0.01; compared to the model group, ##P<0.01.

Table S2 Whole blood viscosity (WBV) ($\bar{x} \pm s$, n=6)

	WBV (mPa·s)				
	1/S	5/S	30/S	50/S	200/S
Control	20.66±0.55	9.05±0.16	5.16±0.06	4.66±0.06	3.85±0.05
Model	26.51±0.78**	10.94±0.27**	5.88±0.12**	5.24±0.10**	4.22±0.08**
Aspirin	24.33±0.36#	10.26±0.14	5.64±0.08	5.05±0.07	4.11±0.05
FDT	22.02±0.26##	9.43±0.07##	5.26±0.027##	4.73±0.02##	3.87±0.02##
PRA	20.44±0.32##	9.02±0.14##	5.18±0.09##	4.68±0.08##	3.87±0.07##
PRR	23.50±0.23##	10.09±0.10##	5.65±0.08	5.07±0.08	4.16±0.08

Note: compared to the control group, *P<0.05, **P<0.01; compared to the model group, #P<0.05, ##P<0.01.

Table S3 Hemorheology and blood coagulation indexes ($\bar{x} \pm s$, n=6)

	EAI	PV(mPa·s)	ESR (mm)	HCT (%)	PT(s)	APTT(s)	TT(s)	FIB (mg/dL)
Control	5.33±0.13	1.38±0.01	1.33±0.21	0.46±0.01	7.48±0.07	25.97±1.45	32.30±0.59	2.37±0.02
Model	6.24±0.12**	1.50±0.01**	10.83±1.92**	0.47±0.01	7.53±0.07	18.47±0.46	26.02±0.24**	5.07±0.20**
Aspirin	5.65±0.06##	1.43±0.02	1.17±0.167##	0.45±0.01	7.65±0.06	21.42±1.09	28.93±0.71##	3.78±0.32#
FDT	5.89±0.05	1.39±0.02##	1.83±0.65##	0.45±0.01	8.17±0.15#	23.17±0.85	28.98±0.36##	3.30±0.23##
PRA	5.28±0.07##	1.39±0.01##	5.00±1.55##	0.43±0.02	8.27±0.18##	27.47±1.68	30.70±0.60##	3.45±0.31##
PRR	5.66±0.11##	1.42±0.04#	2.17±0.48##	0.47±0.01	8.33±0.17##	35.72±4.41##	31.50±0.59##	3.53±0.44##

Note: compared to the control group, *P<0.05, **P<0.01; compared to the model group, #P<0.05, ##P<0.01

Erythrocyte aggregation index (EAI); Plasma viscosity (PV); Erythrocyte sedimentation rate (ESR); Hematocrit (HCT); Prothrombin time (PT); Activated partial thromboplastin time (APTT); Fibrinogen (FIB); Thrombin time (TT)
